# Supplementary material for: Digital solutions to follow up on discharged new parents—A systematic literature review
Source: PLOS Digit Health. 2023 Aug 23;2(8):e0000317. doi: 10.1371/journal.pdig.0000317 (PMC10446181; doi:10.1371/journal.pdig.0000317)
Supplement: Attachment — Submitted filename: PDIG-D-23-00068_R1_reviewer.pdf [file pdig.0000317.s004.pdf]

PLOS Digital Health

Digital solutions to follow up on discharged new parentsa systematic literature review

--Manuscript Draft--

**Manuscript Number:**	PDIG-D-23-00068R1
**Article Type:**	Review
**Full Title:**	Digital solutions to follow up on discharged new parentsa systematic literature review
**Short Title:**	Digital solutions to follow up on discharged new parents
**Corresponding Author:**	Zada Pajalic, Professor PhD<br>VID Specialized University: VID Vitenskapelige Hogskole<br>Oslo, Norway NORWAY
**Order of Authors:**	Zada Pajalic, Professor PhD
	Alona Rauckiene
	Grethe Savosnick
	Irena Bartels
	Jean Calleja Agius
	Diana Saplacan
	Sigridur Sia Jonsdottir
	Babak Asadi-Azarbaijani
**Keywords:**	
**Abstract:**	New parents and their newborns are followed up after discharge either through home visits from midwives/nurses or using information and communication technology. This follow-up focuses on individual needs related to breastfeeding and infant feeding, practical advice on caring for babies, supporting and strengthening the new mother's knowledge and self-confidence concerning child development and parenting skills, and supporting the relationship between mother and baby. This systematic review aims to integrate available research results that describe new parents' perceived sense of security when health and care providers used telemedicine as a platform for follow-up. This literature review was conducted following the PRISMA statement and was prospectively registered in PROSPERO CRD42021236912. The studies were identified through the following databases: AMED, Academic, EMBASE, Google Scholar, Ovid MEDLINE via PubMed, Cochrane database and CINAHL. Results from these studies were compiled using thematic analysis. A total of 886 studies were identified. Screening resulted in eight studies that met the inclusion criteria. Thematic analysis produced the following themes: a) flexible digital support, b) digital literacy, c) safe parenting with digital support, and d) adequate substitute for physical meetings. New parents who live in a home environment with a relaxed atmosphere and around-the-clock digital support experience a sense of control, security, full attention, and encouragement.<br>Digital follow up at home has proven effective because it can meet the support needs of new parents when necessary.
**Additional Information:**	
**Question**	**Response**
**Government Employee**<br><br>Are you or any of the contributing authors an employee of the United States government?	No - No authors are employees of the U.S. government.

Manuscripts authored by one or more US Government employees are not copyrighted, but are licensed under a CC0 Public Domain Dedication, which allows unlimited distribution and reuse of the article for any lawful purpose. This is a legal requirement for US Government employees.<br><br>This will be typeset if the manuscript is accepted for publication.	
**Financial Disclosure**<br><br>Enter a financial disclosure statement that describes the sources of funding for the work included in this submission and the role the funder(s) played. This includes grants and any commercial funding of the work or authors.<br><br>This statement will be typeset if the manuscript is accepted for publication.<br><br>Review the submission guidelines and the instructions link below for detailed requirements and guidance.	The authors received no specific funding for this work.
**Competing Interests**<br><br>On behalf of all authors, disclose any competing interests that could be perceived to bias this work.<br><br>This statement will be typeset if the manuscript is accepted for publication.<br><br>Review the instructions link below and PLOS Digital Health's competing interests policy to determine what information must be disclosed at submission.	The authors have declared that no competing interests exist

Response to reviewers

[PDIG-D-23-00068] - [EMID:ea1d41519e19641e] Digital solutions to follow up on discharged new parents a systematic literature review

We are very grateful for your willingness to consider our manuscript for publication in PLOS Digital Health. We would like to thank the editor and reviewers for their constructive and thoughtful feedback and have revised the manuscript according to the reviewers' concerns below. Changes in the manuscript are highlighted in colour in a separate attached file.

Editor Comments	Response to the Editor
Please choose appropriate keywords based on the MeSH terms.	After checking in MeSH NCBI, the keywords are updated as followed:<br>Ambient-Assisted Living, eHealth, telehealth, parents, postpartum period, patient discharge (lines 44-45)
Please merge the aim of the study with the rest of the introduction section. Moreover, please justify why conducting a SLR was necessary	Aim is merged.<br>We added followed text (lines 91-95)<br>With this review, we want to increase our knowledge of new parents' subjective perceptions, evaluations and interpretations regarding digital follow-up from the care provider's side after discharge from the maternity ward. The new understanding will give us insight into what is perceived to be working well and what challenges remain.
Please make sure that the structure of your manuscript (headings and subheadings) has been organized based on the PRISMA checklist. Please upload the completed check list along with your revised manuscript	Thanks for the comment; this is now under control and subheadings are named as in the PRISMA checklist
In the methods section, please justify why only qualitative studies were included	Added lines 111-112
Please explain why databases such as Scopus and Web of Knowledge were not used while these are the main databases for conducting a SLR	We included databases that mainly have research in nursing and health areas, but at your suggestion, we carried out searches in Scopus and have not found any new studies that answer the purpose of the study.<br>Web of Knowledge we do not have access to via our library
Is there any reason for choosing the timeline between 2015 and 2022	Anledningen till att vi valde denna tidsperiod är baserad på att hitta alla de senaste studierna som svarar på syftet.
Please add a list of search strategies for all databases as an Appendix	We attached text from Word documents, and searches generated as PDF are uploaded separately
In Figure 1, excluding papers after reading the full text, the authors excluded 7 papers indicating that "7 had a low CASP assessment". This needs to be expanded in the methods section and please support it with a reference. Moreover, in this box, reasons other than the CASP score need to be reported	Added lines 152- 153 CASP assessment ranges from 0 -10. Ten points mean that the study is of high quality. See the attachment in the manus after section references Table 1. CASP assessment of included studies

Table 3 is a bit difficult to follow. Could you please remove or summarize it	
Please re-check the captions of the Figures and Tables. In my opinion, e.g. Figure 3 is a Table not a Figure	Removed, but attached as an attachment to the request (above) to show all searches/strategies in all databases
Reviewer #1	Response to Reviewer #1
1) Objectives: “This systematic review aims to integrate available research results that describe parents’ perceived sense of security when health and care providers used telemedicine as a platform for follow-up.” The main objective of the study is not parallel to the results and discussion. Sense of security is too vague and does not reflect the themes stated in the introduction.	Thanks for the important comment, we adjusted the purpose more clearly now<br>This systematic review aims to integrate available research results that describe parents’ experiences when health and care providers used telemedicine as a platform for follow-up
2) Methodology: How was “sense of security” assessed/measured? Was it uniform among the studies that were selected?	This cannot be measured in a qualitative study, only described as an experience. The experiences are what is common to included studies
3) Whole Results section - Please put specific in-text citations or supporting references for the sentences in the Results section. The references were only placed as a range “[24-31]” at the start of every section. It would be better to place the specific reference/s after every statement or so. This would help us readers check the references faster in relation to the claims in the results.	All results from selected studies constituted the unit of analysis. The analysis went across the entire analysis unit to find themes that answered the purpose of the study. Therefore, all included articles were referred to under the respective theme.
4) Results - Flexible Digital Support: “Various digital platforms provided a combination of asynchronous and synchronous methods to facilitate communication with parents out of hospital. Asynchronous communication offered an accessible way to obtain help after early discharge from maternity units.” Maybe you can discuss and expound more on the asynchronous and synchronous methods used in the studies. What asynchronous methods were used? What synchronous methods were used? Are all of them effective and useful for follow ups?	Explanation added at lines 175-176
5) Discussion: The discussion was well-written but it would be better to align it to the objective and results. Emphasize the answer to your objective. Emphasize on discussing your results.	thanks for the comment, we've aligned the most important results with aim of the study this on lines 282-283
1) Typographical error: Please add a period at the end of the sentence in the objective “This systematic review aims to integrate available research results that describe parents’ perceived sense of security when health and care providers used telemedicine as a platform for follow-up”	Added
Reviewer #2	Response to Reviewer #2
Abstract, New parents or new mothers? The authors refer to these interchangeably.	thanks for this comment, we have now corrected this throughout the text

Introduction- Sentence is unclear. Suggestion: Parity, birth-related factors, and psychosocial predictors factors indicate when it is appropriate for a new mother to be discharged and involved in the organisation of to postnatal care	Thanks for the generous suggestion, we accept it and put it in text lines 48-50
Define ‘affinity’.	thanks for the comment, it was completely the wrong word, it should say family bond see line 53
Only fathers? Or partners of any gender?	thanks for comment, it should say partners
Repetition: There is an international trend in welfare countries with social welfare policies	we could not find duplicates but we shortened the text a little line 72
Informal tone: ‘…but is about two days or fewer’ (Suggest- approximately)	thanks, accepted proposal line 75
‘…the provision of continuous care’ – define continuous and the context	should it be written Continuity of care is concerned with the quality of care over time line 94
‘perceived sense of security’ – this seems too subjective – suggest: perceptions of support	thanks for the comment. These concepts are gone and replaced with experience
Inclusion criteria- Refers to ALL types of telemedicine but no reference is made to the exclusion of terms including e-health, m-health or social media	thanks for the comment, this is now revised line 129-130
Abstract/introduction refer to follow up including breastfeeding and infant feeding, and to midwives: please justify why these are then excluded from the search terms.	the introduction describes how and who follows up new parents, this has nothing to do with inclusion criteria or search terms
Please justify and discuss implications of your included studies having only four lead authors. This is a very limited insight which means claims are very bold e.g. the wider literature does not support that digital support is adequate without physical postnatal care.	Thanks för the comment, we have clarified that on lines 382-386
Results- Please define and describe each theme clearly in the opening sentence for each. Also identify which study contributed to each theme within each discussion – it is not clear where there are common findings and how the review synthesises these. The content of your Overview of themes and codes is much clearer about the differentiations than your writing. (NB This is listed as a figure when it is a table).	All results from selected studies constituted the unit of analysis. The analysis went across the entire analysis unit to find themes that answered the purpose of the study. Therefore, all included articles were referred to under the respective theme. The overview of themes and codes illustrates how the analysis has been carried out and can be seen as a complement to the method description and stops there. Under results, each theme is described separately without again reproducing how they came about.
Be specific about the platforms identified here: ‘Various digital platforms provided a combination of asynchronous and synchronous methods to facilitate communication with parents out of hospital.’	Explanation added at lines 175-176
All themes need tighter description/names and linking to the supporting studies within the explanations for each. Content overlaps between each theme. E.g.	All results from selected studies constituted the unit of analysis. The analysis went across the entire analysis unit to find themes that answered the purpose of the study. Therefore, all

	included articles were referred to under the respective theme. The overview of themes and codes illustrates how the analysis has been carried out and can be seen as a complement to the method description and stops there. Under results, each theme is described separately without again reproducing how they came about.
Flexibility and convenience of digital support is a clearer name for theme 1<br><br>Digital literacy – please remove the references to security and safety here – there is too much crossover with safety themes. Fully explain the implications of digital literacy on accessing this support if this theme has been identified.	Thanks for the suggestion; we accept it. Line 180<br>As for the references, let's have them here since the themes are the result of included articles. The themes are the result of qualitative analysis of the results of included articles. This means that we describe the interpreted themes, and it does not work if we follow the method of giving a full explanation that is requested. . Thematization means a higher degree of organization and level of abstraction of the material.
Safe parenting – implies parenting IS safe rather than parents feeling safe. Suggest this theme is renamed to reflect this, and the inclusion of the significance of being at home as a finding.	Thanks for the suggestion; we accept it. Line 249
adequate substitute for physical meetings – this finding is not explored adequately or linked to the studies and is not supported by the wider literature on postnatal care/ ehealth. Please justify with the evidence form the included studies.	Thanks for the comment, but we disagree with the reviewer. As explained above, themes result from qualitative analysis and cannot be explained as requested. Thematization means a higher degree of organization and level of abstraction of the material.
Discussion-Our findings are supported by Wilson et al.’s study – formalise sentence structure and place reference within the sentence not at the end throughout. Also expand – how does this evidence support your findings and why is it not included in your review?	Thanks for pointing out. We have revised the text on lines 291-295. Wilson et al. did not meet the inclusion criteria and could not be included in this study's results. But we have chosen to reflect the results of our study with it.
Please clarify the relevance of this: In medical logistics, drones can deliver necessary treatments to remote areas, collect samples, and even provide emergency assistance	We have clarified the meaning of lines 303- 304
Do not start a sentence with ‘And’ : And in maternity care, telemedicine platforms have been shown to be clinically effective and to improve patient satisfaction [35].<br>Specifically, clinically effective how?	Corrected line 306
This sentence is unclear: The present study indicated that new parents were empowered when they had more knowledge of and facility with digital platforms than the health professionals	Corrected line 309
The present study? Do you mean this review?<br>What does more knowledge and facility mean? It implies parents felt empowered if they were better at using digital platforms than professionals?<br>Please link your claims by referencing the studies you reviewed.	Correct<br>This introduction summary of the themes Digital literacy, referenced
This observation increases patient autonomy, which in turn affects the care relationship [40]. – How? In what ways? Please be specific and synthesise these findings with the conclusions you draw from your review.	Thanks for this comment, criticism is on, place and we have removed the text

Parents living in remote areas or lacking the support of relatives or friends are often reliant on telemedicine as a substitute for physical meetings. -Please reference all such points and link to your findings.	corrected
The expertise obtained from existing telemedicine facilities operating in remote and rural areas has helped to shape digital follow-up after postnatal discharge [46]. – This is a paragraph which needs expanding and linking to the specific findings of your review – how does this evidence impact that you have reviewed?	clarified on lines 326-328
Your discussion appears to focus more on other studies than those you have reviewed, and no links are made to these findings.	Discussion is structured in this way: summary of the most important findings in our review and then mirrored with studies that either confirm or say otherwise.
This review has shown that most new parents have digital literacy, likely driven by the internet and the rapid increase in information and communication technology – this is an overly bold statement based on very few studies with even fewer authors. Consider 'many new parents may….'<br>according to the eHealth competence model) – please define and expand on this and its use in exploring your findings.	Thanks for the comment; Reference should be made to Reddy et al. see line 329
how the concept of care changes when, for instance, robots are introduced as part of the care-giving process – define and expand – what concept of care? How does it change it? How does this impact parents/ the potential of online support?	Thanks for this comment, criticism is on, place and we have revised text line 343-344
it should be noted that the care received after discharge may be experienced differently than a home visit from a healthcare professional. – on what ways may it be experienced differently? What impact may it have (other reviews have noted the importance of concurrent support across settings)	Thanks for this comment, criticism is on, place and we have removed the text
Following a recent study describing autonomy when interacting or using digital tools as relational and situated [52], autonomy needs to be seen in context and in relation to the users' situated abilities – define and explain these terms and the relevance of them to parents.	Thanks for this comment, criticism is on, place and we have removed the text
However, one aspect that has not been addressed is whether new parents experience the use of digital solutions differently. Differently to whom?	Thanks for this comment, criticism is on, place and we have removed the text

Upon discharge from hospital, new parents – even those with high digital literacy – may find it challenging or overwhelming to use digital tools whilst also caring for a newborn. Reference your sources.	This is the reviewer's conclusion. We base our conclusion on an analysis of included articles
Indeed, telemedicine has proven to be important for women with low socioeconomic status, particularly in relation to addressing postnatal mental health [55]. How does this relate to the previous point?<br><br>In the future, more advanced platforms or technical solutions will be available, such as social and assistive robots. Robots could be used to assist with practical tasks within the home (e.g.,cleaning, and collecting and transporting items within the home) and for eHealth purposes (e.g., getting in touch with a healthcare professional or giving advice to new parents). Such robots have been tested with older adults – see for instance, the use of the Giraff robot [56-58] or other social and assistive robots (SARs) [59]. However, using SARs to support new parents following discharge remains understudied and represents an area for further research. Newly discharged parents often need help with practical tasks around the home so they can focus on their newborn; some of these can potentially already be allocated to robots [50] as they do not involve direct, intimate parent–robot interaction. On the other hand, tasks that involve more 16 interaction between the robot and the newly discharged parents, like moving and bringing items, giving advice, and helping parents get in touch with healthcare professionals, may be interesting to investigate further. However, privacy, safety, and security issues may arise depending on how advanced the robot is and what equipment is used (e.g., cameras and sensors) [60]. Rigorous pilot testing and proper clinical trials are therefore needed before these digital applications are offered to new parents discharged early from hospital.<br><br>The above seems irrelevant to your findings and the scope of your review. You need to properly define the parameters of 'postnatal care/support'. You have also not explained the terms discussed e.g Giraff robot and what they do. Please remove or clearly explain the links to your findings.	Thanks for this comment, criticism is on, place and we have removed the text
Implications for practice<br><br>After early home discharge, new parents should have reliable, 24-hour access to information related to newborn care, breastfeeding, and parenthood. - You have not clearly identified how and which of your themes have demonstrated this.	Thanks for this comment, criticism is on, place and we have revised the headline lines 388-390
This is especially important when supporting breastfeeding while also ensuring security and cultural appropriateness. - Other than in your abstract you have not referred anywhere to	Thanks for this comment, criticism is on, place and we have revised the headline lines 388-390

breastfeeding support, nor included this in your search terms – this is therefore not a claim supported b your review.	
oday's new parents tend to be digitally literate and are thus very likely to make use of telemedicine. – again there are no figures or references to the literature to justify this claim. I suggest Statista.	Thanks for this comment, criticism is on, place and we have revised the headline lines 388-390
Conclusion<br><br>in contrast, problems can arise if parents lack access to digital resources after early discharge. – You have not demonstrated this to be the case with your findings or links to wider evidence.<br><br>Finally, digital literacy is important for both healthcare professionals and patients receiving care through telemedicine. – You have not provided sources or reference to digital literacy amongst professionals elsewhere	Thanks for this comment, criticism is on, place and we have revised the headline 393-399
This area needs to be developed in the healthcare system, especially among midwives, who mainly provide this support in the context of postnatal follow-up care. Again you need to explore the literature on midwives and digital support to claim this. See Morse & Brown, 2021 and 2022.<br><br>Additionally: you have provided no limitations to your review of which there are several, including the limited number of included papers, the search terms which do not include breastfeeding or social media or midwives. Please add a limitations section.	Thanks for this comment, criticism is on, place and we have revised the headline lines 393-399

Manuscript Click here to access/download;Manuscript;03-05-2023 Revised Manuscript withouth Track Changes.docx

Digital solutions to follow up on discharged new parents - a systematic literature review

Author names:

Zada Pajalic[1], Alona Rauckiene[2] Grethe Savosnick, Irena Bartels[3] , Jean Calleja Agius[4], Diana Saplacan5, Sigríður Sía Jónsdóttir[6] & Babak Asadi-Azarbaijani[1]

Affiliations

[1]Faculty of Health Sciences, VID Specialized University, Norway

[2] Department of Health Research and Innovation Science Centre, Klaipeda University, Lithuania

[3]East Tallinn Central Hospital, Estonia

[4]Department of Anatomy, Faculty of Medicine and Surgery, University of Malta, Malta

[5]Department of Informatics, University of Oslo, Norway

[6]School of Health Science, University of Akureyri, Iceland

Corresponding author

Zada Pajalic

Zada.pajalic@vid.no

Digital solutions to follow up on discharged new parents

# - a systematic literature review

Abstract

New parents and their newborns are followed up after discharge either through home visits from midwives/nurses or using information and communication technology. This follow-up focuses on individual needs related to breastfeeding and infant feeding, practical advice on caring for babies, supporting and strengthening the new mother's knowledge and self-confidence concerning child development and parenting skills, and supporting the relationship between parents and baby. This systematic review aims to integrate available research results that describe new parents' experiences when health and care providers used telemedicine as a platform for follow-up after discharge from the childbirth department. This literature review was conducted following the PRISMA statement and was prospectively registered in PROSPERO CRD42021236912. The studies were identified through the following databases: AMED, Academic, EMBASE, Google Scholar, Ovid MEDLINE via PubMed, Cochrane database and CINAHL. Results from these studies were compiled using thematic analysis. A total of 886 studies were identified. Screening resulted in eight studies that met the inclusion criteria. Thematic analysis produced the following themes: a) *Flexibility and convenience of digital support*, b) *Digital literacy*, c) *Parents feeling safe with digital support* , and d) *Adequate substitute for physical meetings*.

New parents who live in a home environment with a relaxed atmosphere and around-the-clock digital support experience a sense of control, security, full attention, and encouragement.

Digital follow up at home has proven effective because it can meet the support needs of new parents when necessary.

Keywords: Ambient-Assisted Living, eHealth, , telehealth, parents, postpartum period, patient discharge

1.0 Introduction

Parity, birth-related factors, and psychosocial predictors factors indicate when it is appropriate for a new mother to be discharged and involved in the organisation of postnatal care. Discharge is when a healthy mother and baby return home from hospital after a birth [1]. Parity, birth-related factors, and psychosocial predictors indicate when a new mother can be discharged and involved in the organisation of postnatal care [2]. Family bonding, which is vital for the whole family, can be achieved more rapidly if the family is together at home and if partners are involved from birth [3].

Potential advantages of early discharge for new parents and their babies include a peaceful home environment, positive impact on breastfeeding, enhanced emotional well-being, minimised exposure to infection risks, and increased parental relaxation and involvement in child care [4]. Follow-up of new parents after discharge is usually offered by the midwife [5] via home visits [6] or telemedicine [7]. This follow-up often focuses on individual needs related to breastfeeding and infant feeding, practical advice on caring for babies, supporting and strengthening the new mother's knowledge and self-confidence, child development, parenting skills, and supporting the mother–child relationship [8].

Telemedicine is an umbrella term for the use of telecommunications technology to provide medical support at a distance [9]. Telemedicine is divided into different categories based on the interaction between users and the type of information used. The interaction can take place in real-time (synchronous) or be pre-recorded (asynchronous), and the information can be transmitted between users via audio, text, images, and videos [9]. A similar concept to telemedicine is eHealth, a term that comprises health service delivery between healthcare professionals and patients through the internet and related technologies [10]. Telemedicine

offers new possibilities and enables support for many patients remotely in a cost-effective way [11].

There is an international trend in welfare countries to shorten the postpartum length of stay in hospitals, driven by cost containment and hospital bed availability [12]. The average postpartum stay after vaginal delivery varies between countries, approximately [1]. Discharge after childbirth can be challenging for new parents, many of whom have a great need for practical support and expert guidance. As such, early discharge requires integrated care services, 24-hour availability of qualified healthcare professionals for advice and support, home visits and the opportunity for home support from qualified healthcare professionals [13]. Moreover, feeling safe following an early return home depends on whether parents make that decision themselves and whether breastfeeding has commenced [14]. Research shows that parents perceive discharge as positive when they receive adequate follow-up and help from healthcare professionals and know who to contact if needed [13] . Parents experience early discharge after birth as successful if they a) receive follow-up from a midwife and home visits after they return home, with the opportunity to obtain answers to questions when they arise; b) receive sufficient information; and c) are able to form a trusting relationship with the midwife [15].

Postpartum care must be based on a woman's decisions, clinical indications, family preferences, and care context [16]. Planned follow-up care has a significant impact on the health and well-being of parents, children, and families—both in the short and the long term [17, 18]. Research shows that home-based postnatal care is well accepted by new parents who are discharged after childbirth [19]. Moreover, when parents and partners feel empowered by their midwife, this enhances feelings of self-confidence and a sense of security after discharge from a maternity unit. All postpartum interventions related to the provision of continuity of care (quality of care over time) are associated with satisfaction and impact the family's psychological and social outcomes [16]. With this review, we want to increase our knowledge of new parents' subjective

perceptions, evaluations and interpretations regarding digital follow-up from the care provider's side after discharge from the maternity ward. The new understanding will give us insight into what is perceived to be working well and what challenges remain.

"This systematic review aims to integrate available research results that describe parents' experiences when health and care providers used telemedicine as a platform for follow-up after discharge from the childbirth department".

2.0 Methods

The systematic review was performed following the PRISMA statement and was prospectively registered in PROSPERO [20] (no. CRD42021236912).

2.1 Data sources and search strategy

The PROSPERO database was searched to determine whether similar studies had been completed or were still in progress. We could not find any registered studies with the same aim as ours.

2.2 Eligibility criteria

The review aimed to search for peer-reviewed primary qualitative studies that responded to the study's objective. Studies with a qualitative design describe parents' experiences, which answers the purpose of the study. The searches were performed between 030121-311221. A new search was carried out in October 2022. No new studies meeting the inclusion criteria were found.

2.3 Inclusion criteria

- All types of telemedicine used by healthcare professionals to follow-up discharged new parents from the childbirth department
- New parents discharged to their own homes

- Studies focusing on support in private homes
- Various ways that technology may be administrated, such as internet infrastructure implementation and individual provision
- Qualitative studies
- Welfare countries

2.4 Exclusion criteria

- **usual medical follow-up (e.g. return visits to hospital, home visits, follow-up at care centers)**
- Not target population
- Not primary study
- Protocol, review articles
- Books
- Studies in hospitals
- Quantitative studies

2.5 Information sources

At VID Specialised University in Oslo, Norway, a science librarian and first author (ZP) identified primary studies in the following bibliographic electronic databases: AMED, Academic, EMBASE, Ovid MEDLINE via PubMed, Google Scholar, Cochrane database and CINAHL.

2.6 Search strategy

Systematic searches were conducted for studies published in English, Norwegian, and Swedish. The searches were limited to the period between 2015 and 2022 to include the newest studies. The search strategy incorporated the following MeSH keywords singly and in

combination with each other (combined with the Boolean operators OR and AND): patient, birth, early, discharge, postpartum, experience, length of stay, parent, semi-structured interviews, cross-sectional, sense of security, follow-up, health system, primipara, multipara, home-based postnatal care, midwife, home visits, ambulatory care, health service, managed care, aftercare, self-care, fear of infection, telemedicine, expectations.

2.7 Study selection and review process

In total, 886 studies were identified through literature searches and were first imported into the EndNote reference manager [21] and then into Rayyan [22] (a web tool for systematic reviews that aids in the comparison of decisions regarding inclusion or exclusion of studies). All duplicates (n=136) were removed resulting in 750 publications. Titles and abstracts for the 750 publications were then screened according to inclusion and exclusion criteria. Of these, 58 were screened in full text, 15 of which were assessed for quality using the Critical Appraisal Skills Programme (CASP) [23] checklist for qualitative studies. CASP assessment ranges from 0 -10. Ten points mean that the study is of high quality. Of these 15 publications, 8 [24-31] were judged to have the best combination of relevance and quality (see PRISMA Flow chart and Tables 1 and 2).

Screening of titles and abstracts was performed by all co-authors, first independently and then together. In the case of conflicting independent decisions, the authors reviewed the titles again together and discussed them to reach consensus. Full text screening and CASP quality review were performed by all authors. The final decision of which studies to include was made by all reviewers.

2.8 Data synthesis

We chose to compile results from the selected articles using thematic synthesis, following Braun and Clarke [32]. Analysis of selected study results was performed by BAA and ZP and discussed with all participants until consensus was reached.

The first phase of our work was to extract the results from the selected studies in a separate document. After reading the text several times, we identified initial codes, which we then grouped into different themes. All co-authors critically reviewed the potential themes using a process of dialogue and consensus. Finally, we defined and named the following themes: a) flexible digital support, b) digital literacy, c) safe parenting with digital support, and d) adequate substitute for physical meetings (Table 3 and Figure 3).

3.0 Results

3.1 Flexibility and convenience of digital support

This study showed that the sharing of information between parents and healthcare professionals was essential [24-31]. Various digital platforms provided a combination of asynchronous (chat bots) and synchronous (staff respond directly via audio or video) methods to facilitate communication with parents out of hospital. Asynchronous communication offered an accessible way to obtain help after early discharge from maternity units. New parents appreciated having this information available, as well as an online chat option. This was also an excellent way to refresh the knowledge of those who already had children. Most parents found the new communication methods for contacting healthcare professionals easy to use and that they conveyed a range of information effectively, thus saving time. All parents felt safe because they had access to the information they needed. It should be noted, however, that in terms of parents' ability to find and retrieve information, digital literacy was crucial.

This review also identified 24-hour access to information and the ability to access the same information repeatedly as additional positive aspects. Digital platforms provided the

opportunity to distribute information based on current needs so that even new parents could share their knowledge and experiences via chat functions. Written information proved to be easily accessible around the clock, whether through an application (app), chat, or short text messages. Some groups also had access to web pages containing answers to the most frequently asked questions. Digital platforms created and led by midwives were perceived as the most credible sources for parents. Being able to receive rapid answers to questions that arose throughout the day or night made several new parents feel closer to their midwives and that they were being given the midwives' full attention whenever they needed it. Access to information thus gave parents a sense of control, security, and reassurance.

In addition, it was important for most new parents to receive positive feedback and confirmation from healthcare professionals. The flexibility that digital communication with healthcare professionals offered gave the parents a feeling of control and peace. New parents also stated that it was easier and more convenient to communicate digitally, and that sending a message was less disruptive. Some participants preferred asynchronous video instruction rather than face-to-face guidance from healthcare professionals because they could watch the videos whenever they had time and view them repeatedly if desired. In one of the studies, the visual contact established via videoconference facilitated a warm relationship between parents and healthcare professionals. The parents could see the nurses acknowledging their facial expressions and body language during their conversations when parents shared their observations related to their babies with the nurses. In this way, parents felt they were receiving the full attention of the healthcare professionals.

3.2 Digital literacy

Another theme that emerged was digital literacy among parents. Some study participants expressed that they were both surprised by and unprepared for early discharge after delivery; indeed, some had negative experiences because of this and had felt unwelcome [24-31]. Many

of the new parents in the studies would have chosen to stay longer in hospital, with the main reasons being the need for breastfeeding support and help interpreting newborns' signals. However, the ability to use digital technology once they arrived home enhanced parents' feelings of security and gave them a sense of control.

All of the parents in the studies rapidly oriented themselves by using the media offered by the hospital as a support at home. Digital platforms with direct or asynchronous communication provided when needed reduced parents' feelings of insecurity about their new role. Asynchronous provision of information also helped meet the need for more frequent support. In addition, new parents reported using digital platforms (e.g. social media) as an alternative support strategy, seeking information on their own if they felt they were receiving insufficient support, were uncertain about something, or questioned the advice given. Some participants even wanted to compare the information they were receiving.

The most pressing need was to have more accessible personalised information on digital platforms. Every task that the parents accomplished via digital support strengthened their self-confidence and helped create a positive experience. Their self-confidence grew alongside their increased understanding of their baby's behaviour. Another freedom that the parents discovered with digital support was that they could ask any questions they desired, without feeling ashamed, which they found liberating. In step with increased self-confidence, some new parents also chose to share their experiences with other parents through this platform because they felt confident.

One interesting finding is that, in some cases, there was a mismatch in digital competence between healthcare professionals and parents, with healthcare professionals being at a disadvantage. When it became evident that the parents had better digital skills than the midwives, this led to a disparity in knowledge of how to navigate digital platforms between new parents and healthcare professionals. Some parents appreciated that they could help the

midwives improve their digital competence via interactive collaboration; they felt confident regarding their capacity and ability to use the app or improve healthcare professionals' digital skills. By establishing some control, in addition to helping them feel prepared and strong in their new role, this kind of mutual knowledge exchange became a way to further empower parents.

3.3Parents feeling safe with digital support

The results showed that digital follow-up met the parents' need for emotional support and gave them a sense of security [24-31]. Several parents reported that the digital follow-up was better than they expected to be. It was essential for participants to know that they could manage everything themselves and feel safe in their new role as parents.

For the parents, the most positive aspect was being able to communicate directly with the healthcare professionals and feeling comfortable and natural in front of the web camera. This contributed to a sense of control and made it easier to absorb information. Many likened the digital follow-ups at home to a digital lifeline. Indeed, new parents who could obtain the information they needed at home reported improved emotional well-being. They felt safe and relaxed, found it easier to seek help, and had more self-confidence when performing routine infant care tasks. It was important for parents to feel comfortable and supported with clarifying information whenever uncertainty arose.

New parents were positive about several aspects of the home environment. Many of them found it more comfortable to stay in their home environment, as long as it had a relaxed atmosphere. They felt that they had more control and could more easily follow their routines and roles in a quiet home environment compared to when they were in hospital. New parents who felt prepared for early discharge after giving birth expressed that a quiet home environment helped them to relate better to their child. Others reported that being at home enabled them to establish

a circadian rhythm around the care of their newborn baby. They were also able to learn the baby's signals at their own pace. Moreover, at home, the parental roles became more apparent, as did the feeling of being a family, with the opportunity to focus on each other's needs and support each other. Many reported an increase in self-confidence and feelings of competence. In summary, a home environment with access to digital support when needed helped parents feel that they could safely handle their new role.

3.4 Adequate substitute for physical meetings

As noted earlier, several new parents shared their experiences with other parents, and digital meetings were a good substitute for physical meetings [24-31]. In some studies, the new parents had limited access to digital support and most of them wanted more. Conversations via webcam helped participants to communicate more effectively because the camera enabled midwives to see what parents were talking about and thus understand the issues at hand more efficiently than when they were only described orally. After trying out various digital support systems, most new parents concluded that these were better alternatives than staying longer in hospital. As mentioned above, the majority expressed that technology was a lifeline to professional support around the clock. Some felt that even automated answers constituted a good form of support, especially those that suggested practical solutions; these helped parents avoid unnecessary hospital visits. Parents also reported having better access to healthcare professionals through the app than when they were in hospital. Many also found it easier to ask more questions in the app than in face-to-face meetings.

4.0 Discussion

The results from the present study highlight the new parents' experience based on sustainable and flexible digital support for parents discharged early after childbirth [24-31]. Such support is especially important for new parents, who may feel that they lack confidence in their ability to care for a newborn. Our findings are supported by Wilson et al.'s study, which highlights the

healthcare sector’s suitability for digital follow-up of discharged patients. The healthcare sector can improve health outcomes using digital support for patients. Digital solutions make it possible distribution of resources efficiently and fairly. Digital health interventions are context-specific and a constantly changing process. [33]. Digital follow-up makes it possible to reach a greater number of people, provide increased service coverage, and reduce healthcare costs. Indeed, Awad et al. argue that digitised support for patients in the home environment is one of the most promising developments in modern care when the technology is strategic, well thought out, and adapted to individual needs [34]. Moreover, digital devices enable rapid diagnosis, individualised treatment, and interventions such as precision surgery, symptom monitoring counselling, rehabilitation, or targeted drug delivery. An excellent example of a focus on digital support is in medical logistic aredrones that can deliver necessary treatments to remote areas, collect samples, and even provide emergency assistance [11]. In maternity care, telemedicine platforms have been shown to be clinically effective and to improve patient satisfaction [35].

This review indicated that new parents were empowered when they had more knowledge regarding digital platforms than the health professionals [24-31]. This is supported by Alsem et al., who found improved parental satisfaction when parents used digital tools to prepare for consultations with healthcare professionals [36]. Alsem et al. report positive experiences of personal and interactional factors determining empowerment, creating conditions for an equal doctor–parent relationship [36]. Moreover, while they found that digital tools proved helpful for parents to explore their needs and find information, they conclude that more research is needed to support autonomy in consultation. Salonen et al. highlight that the new generation of patients are accustomed to using the latest digital media and technological innovations [37-39]. These patients find that healthcare professionals are not always well-trained in using digital platforms [40]. This finding supports the creation of more technological solutions that meet the needs of parents in their first year postpartum [40-42].

Parents living in remote areas or lacking the support of relatives or friends are often reliant on telemedicine as a substitute for physical meetings [8]. As the COVID-19 pandemic has heightened concerns regarding infection, this has led to early hospital discharge and parental hesitancy towards in-person hospital or health centre visits and being physically seen by a healthcare professional, increasing the need for virtual consultations [15, 43, 44]. These have proven especially vital during lockdowns and in instances where parents have COVID-19 [45]. It was shown that the expertise obtained from existing telemedicine facilities operating in remote and rural areas has helped to shape digital follow-up after postnatal discharge [46].

Reddy et al. review has shown that most new parents have digital literacy, likely driven by the internet and the rapid increase in information and communication technology [47]. Around the world, digital health interventions are increasingly being adopted to address various public health issues. However, for users to understand, process, and act on health-related information, six literacy skills are necessary (according to the eHealth competence model): traditional, health, information, science, media, and computer literacy [48]. In the studies we reviewed, despite parents' willingness to support healthcare professionals in using digital solutions, healthcare professionals nevertheless required training in this new method of patient communication. Results from a study by Kuek and Hakkennes [49] indicate that healthcare professionals who had low digital literacy levels experienced anxiety using digital technology. This situation affected the quality of care the patients were offered. Thus, any new digital solutions should first be tested in properly designed trials to assess the level of training needed both by the healthcare professionals and the beneficiary [49].

The present review also shows that while digital solutions can empower parents newly discharged from the hospital, they can also change how care is provided. A previous study discusses how the concept of care changes when, for instance, digital support is introduced as part of the care-giving process [50, 51]. Although the digital solutions explored in this review

are mainly limited to apps and videoconferencing. It seems that digital solutions may represent a compromise between having a home visit from a healthcare professional and receiving no help whatsoever. However, the parents' autonomy, and whether they are empowered by digital tools, should be contextualised [52].

Our review has shown that digital solutions seems to benefit the follow-up of new parents after postnatal discharge, including by giving them 24-hour support. The digital solutions examined were mainly mobile applications, such as the mobile health app (mHealth) for postpartum care and other telemedicine solutions (e.g., videoconferencing) [53]. In addition, mHealth interventions have been shown to be effective in improving maternal and neonatal service utilisation [41, 54]. These digital solutions appear to serve their purpose when care receivers wish to retrieve information by themselves or need to get in touch with healthcare professionals. In addition, the solutions seem free of potential ethical dilemmas, such as those entailed by videoconferencing solutions which can be experienced as an intrusion of privacy due to the use of cameras.

Upon discharge from hospital, new parents – even those with high digital literacy – may find it challenging or overwhelming to use digital tools whilst also caring for a newborn. Indeed, telemedicine has proven to be important for women with low socioeconomic status, particularly in relation to addressing postnatal mental health [55].

In the future, more advanced platforms or technical solutions will be available, such as social and assistive robots. Robots could be used to assist with practical tasks within the home (e.g., cleaning, and collecting and transporting items within the home) and for eHealth purposes (e.g., getting in touch with a healthcare professional or giving advice to new parents). Such robots have been tested with older adults [56-58] or other Social and Assistive Robots (SARs) [59]. However, using SARs to support new parents following discharge remains understudied and represents an area for further research. Newly discharged parents often need help with practical

tasks around the home so they can focus on their newborn; some of these can potentially already be allocated to robots [50] as they do not involve direct, intimate parent–robot interaction. On the other hand, tasks that involve more interaction between the robot and the newly discharged parents, like moving and bringing items, giving advice, and helping parents get in touch with healthcare professionals, may be interesting to investigate further. However, privacy, safety, and security issues may arise depending on how advanced the robot is and what equipment is used (e.g., cameras and sensors) [60]. Rigorous pilot testing and proper clinical trials are therefore needed before these digital applications are offered to new parents discharged early from hospital.

Implications for practice

This study's results are based on included studies with only four primary authors. This review indicates that telemedicine support for new parents has some potential worth investigating in more extensive RCT studies.

Conclusion

Personal counselling is an important component of care in the postpartum period, when new parents have issues specific to the child or mother (including postpartum mental health). A range of digital solutions are suitable in this situation, but a key feature of all such solutions is 24-hour access to information. In a home environment, digital communication gives parents a sense of security and emotional satisfaction; in contrast, problems can arise if parents lack access to digital resources after early discharge. The digital follow-up may be a great asset for new parents, as it can help to reliably inform them and give them the necessary support.

Declarations

Ethics approval and consent to participate

Not applicable

Consent for publication

Not applicable

Data availability statement

All data generated or analysed during this study are included in this published article and additional files including articles included in the analysis which are cited in the reference list.

Competing interests

The authors have declared that no competing interests exist.

Funding

Not applicable

Author Contributions

Conceptualisation: Zada Pajalic

Registration and final reporting in PROSPERO: Zada Pajalic

Searching: Zada Pajalic and Pinar Ekitz

Methodology: Zada Pajalic.

Data analysis: Zada Pajalic and Babak Asadi-Azarbaijani

Writing – original draft: Zada Pajalic, Babak Asadi-Azarbaijani, Alona Rauckiene, Grethe Savosnick, Irena Bartels, Jean Calleja Agius, Diana Saplacan, Sigríður Sía Jónsdóttir

Writing – review & editing: Zada Pajalic, Babak Asadi-Azarbaijani, Alona Rauckiene, Grethe Savosnick, Irena Bartels, Jean Calleja Agius, Diana Saplacan, Sigríður Sía Jónsdóttir

Corresponding authors: Zada Pajalic and Babak Asadi-Azarbaijani

References

1. Campbell OM, Cegolon L, Macleod D, Benova L. Length of stay after childbirth in 92 countries and associated factors in 30 low-and middle-income countries: compilation of reported data and a cross-sectional analysis from nationally representative surveys. PLoS medicine. 2016;13(3):e1001972.
2. Nilsson I, Danbjørg DB, Aagaard H, Strandberg-Larsen K, Clemensen J, Kronborg H. Parental experiences of early postnatal discharge: A meta-synthesis. Midwifery. 2015;31(10):926-34.
3. Persson EK, Dykes A-K. Parents' experience of early discharge from hospital after birth in Sweden. Midwifery. 2002;18(1):53-60.
4. Jones E, Stewart F, Taylor B, Davis PG, Brown SJ. Early postnatal discharge from hospital for healthy mothers and term infants. Cochrane Database of Systematic Reviews. 2021;(6).
5. Pajalic Z, Pajalic O, Saplacan D. Women's education and profession midwifery in Nordic countries. Journal of Health Sciences. 2019;9(3):127-35.
6. Roisné J, Delattre M, Rousseau S, Bourlet A, Charkaluk M-L. Newborn follow-up after discharge from the maternity unit: Compliance with national guidelines. Archives de Pédiatrie. 2018;25(2):95-9.
7. Filchev R, Pavlova D, Dimova R, Dovramadjiev T, editors. Healthcare System Sustainability by Application of Advanced Technologies in Telemedicine and eHealth. Human Interaction, Emerging Technologies and Future Systems V: Proceedings of the 5th International Virtual Conference on Human Interaction and Emerging Technologies, IHIET 2021, August 27-29, 2021 and the 6th IHIET: Future Systems (IHIET-FS 2021), October 28-30, 2021, France; 2022: Springer.
8. Lemyre B, Jefferies AL, O'Flaherty P. Facilitating discharge from hospital of the healthy term infant. Paediatrics & child health. 2018;23(8):515-22.
9. Wootton R, Craig J, Patterson V. Introduction to telemedicine: CRC Press; 2017.
10. Williatte-Pellitteri L. New Technologies, Telemedicine, eHealth, Data... What Are You Talking About? The Lawyer's Point of View. Digital Medicine. 2019:93-102.
11. Barnard-Kelly K. Utilizing eHealth and telemedicine technologies to enhance access and quality of consultations: it's not what you say, it's the way you say it. Diabetes Technology & Therapeutics. 2019;21(S2):S2-41-S2-7.
12. Benahmed N, San Miguel L, Devos C, Fairon N, Christiaens W. Vaginal delivery: how does early hospital discharge affect mother and child outcomes? A systematic literature review. BMC pregnancy and childbirth. 2017;17(1):1-14.
13. Kurth E, Krähenbühl K, Eicher M, Rodmann S, Fölmli L, Conzelmann C, et al. Safe start at home: what parents of newborns need after early discharge from hospital–a focus group study. BMC health services research. 2016;16(1):1-14.
14. Askelsdottir B, Lam-de Jonge W, Edman G, Wiklund I. Home care after early discharge: Impact on healthy mothers and newborns. Midwifery. 2013;29(8):927-34.
15. Barimani M, Vikström A. Successful early postpartum support linked to management, informational, and relational continuity. Midwifery. 2015;31(8):811-7.
16. Crowther S, MacIver E, Lau A. Policy, evidence and practice for post-birth care plans: a scoping review. BMC Pregnancy and Childbirth. 2019;19(1):1-16.
17. Olds DL, Kitzman H, Anson E, Smith JA, Knudtson MD, Miller T, et al. Prenatal and infancy nurse home visiting effects on mothers: 18-year follow-up of a randomized trial. Pediatrics. 2019;144(6).

18. Van Den Heuvel JF, Groenhof TK, Veerbeek JH, Van Solinge WW, Lely AT, Franx A, et al. eHealth as the next-generation perinatal care: an overview of the literature. Journal of medical Internet research. 2018;20(6):e202.
19. Johansson M, Thies-Lagergren L, Wells MB. Mothers experiences in relation to a new Swedish postnatal home-based model of midwifery care–A cross-sectional study. Midwifery. 2019;78:140-9.
20. Page MJ, McKenzie JE, Bossuyt PM, Boutron I, Hoffmann TC, Mulrow CD, et al. The PRISMA 2020 statement: an updated guideline for reporting systematic reviews. Bmj. 2021;372:n71. Epub 2021/03/31. doi: 10.1136/bmj.n71. PubMed PMID: 33782057.
21. Hupe M. EndNote X9. Journal of Electronic Resources in Medical Libraries. 2019;16(3-4):117-9.
22. Kellermeyer L, Harnke B, Knight S. Covidence and rayyan. Journal of the Medical Library Association: JMLA. 2018;106(4):580.
23. Birks M, Mills J. Quality in qualitative research. Qualitative methodology: A practical guide. 2014:221-36.
24. Danbjørg DB, Wagner L, Clemensen J. Do families after early postnatal discharge need new ways to communicate with the hospital? A feasibilility study. Midwifery. 2014;30(6):725-32.
25. Danbjørg DB, Wagner L, Clemensen J. Designing, developing, and testing an app for parents being discharged early postnatally. The Journal for Nurse Practitioners. 2014;10(10):794-802.
26. Danbjørg DB, Wagner L, Kristensen BR, Clemensen J. Intervention among new parents followed up by an interview study exploring their experiences of telemedicine after early postnatal discharge. Midwifery. 2015;31(6):574-81.
27. Garne Holm K, Brødsgaard A, Zachariassen G, Smith AC, Clemensen J. Parent perspectives of neonatal tele-homecare: A qualitative study. J Telemed Telecare. 2019;25(4):221-9. Epub 2018/05/25. doi: 10.1177/1357633x18765059. PubMed PMID: 29792079.
28. Garne K, Brødsgaard A, Zachariassen G, Clemensen J. Telemedicine in neonatal home care: identifying parental needs through participatory design. JMIR research protocols. 2016;5(3):e100.
29. Lindberg I, Christensson K, Öhrling K. Parents' experiences of using videoconferencing as a support in early discharge after childbirth. Midwifery. 2009;25(4):357-65.
30. Shorey S, Ng ED. Evaluation of mothers' perceptions of a technology-based supportive educational parenting program (part 2): Qualitative study. Journal of medical Internet research. 2019;21(2):e11065.
31. Shorey S, Yang YY, Dennis C-L. A mobile health app–based postnatal educational program (Home-but not alone): descriptive qualitative study. Journal of medical Internet research. 2018;20(4):e119.
32. Braun V, Clarke V. Reflecting on reflexive thematic analysis. Qualitative research in sport, exercise and health. 2019;11(4):589-97.
33. Wilson D, Sheikh A, Görgens M, Ward K. Technology and Universal Health Coverage: Examining the role of digital health. J Glob Health. 2021;11:16006. Epub 2021/12/17. doi: 10.7189/jogh.11.16006. PubMed PMID: 34912559; PubMed Central PMCID: PMCPMC8645240 (available upon request from the corresponding author), and declare no conflicts of interest.

34. Awad A, Trenfield SJ, Pollard TD, Ong JJ, Elbadawi M, McCoubrey LE, et al. Connected healthcare: Improving patient care using digital health technologies. Advanced Drug Delivery Reviews. 2021;178:113958.

35. Kern-Goldberger AR, Srinivas SK. Telemedicine in obstetrics. Clinics in Perinatology. 2020;47(4):743-57.

36. Alsem MW, Verhoef M, Braakman J, van Meeteren KM, Siebes RC, Jongmans MJ, et al. Parental empowerment in paediatric rehabilitation: exploring the role of a digital tool to help parents prepare for consultation with a physician. Child: Care, Health and Development. 2019;45(5):623-36.

37. Salonen AH, Kaunonen M, Åstedt-Kurki P, Järvenpää AL, Tarkka MT. Development of an internet-based intervention for parents of infants. Journal of Advanced Nursing. 2008;64(1):60-72.

38. Salonen AH, Kaunonen M, Åstedt-Kurki P, Järvenpää A-L, Isoaho H, Tarkka M-T. Effectiveness of an internet-based intervention enhancing Finnish parents' parenting satisfaction and parenting self-efficacy during the postpartum period. Midwifery. 2011;27(6):832-41.

39. Salonen AH, Pridham KF, Brown RL, Kaunonen M. Impact of an internet-based intervention on Finnish mothers' perceptions of parenting satisfaction, infant centrality and depressive symptoms during the postpartum year. Midwifery. 2014;30(1):112-22.

40. Slomian J, Emonts P, Vigneron L, Acconcia A, Reginster J-Y, Oumourgh M, et al. Meeting the needs of mothers during the postpartum period: using co-creation workshops to find technological solutions. JMIR Research Protocols. 2017;6(5):e6831.

41. Sondaal SFV, Browne JL, Amoakoh-Coleman M, Borgstein A, Miltenburg AS, Verwijs M, et al. Assessing the effect of mHealth interventions in improving maternal and neonatal care in low-and middle-income countries: a systematic review. PloS one. 2016;11(5):e0154664.

42. Uscher-Pines L, Ghosh-Dastidar B, Bogen DL, Ray KN, Demirci JR, Mehrotra A, et al. Feasibility and effectiveness of telelactation among rural breastfeeding women. Academic pediatrics. 2020;20(5):652-9.

43. Rasmussen SA, Jamieson DJ. Pregnancy, postpartum care, and COVID-19 vaccination in 2021. Jama. 2021;325(11):1099-100.

44. Gutiérrez MM-C, Durán-Vila A, Ruiz-Labarta J, Payá-Martínez P, Pintado Recarte P, Bujan J, et al. A new multiplatform model for outpatient prenatal and postpartum care in a cohort of covid-19-affected obstetric patients. International Journal of Environmental Research and Public Health. 2021;18(10):5144.

45. Rasmussen SA, Jamieson DJ. Caring for women who are planning a pregnancy, pregnant, or postpartum during the COVID-19 pandemic. Jama. 2020;324(2):190-1.

46. Brown HL, DeNicola N. Telehealth in maternity care. Obstetrics and Gynecology Clinics. 2020;47(3):497-502.

47. Reddy P, Sharma B, Chaudhary K. Digital literacy: A review of literature. International Journal of Technoethics (IJT). 2020;11(2):65-94.

48. Norman CD, Skinner HA. eHealth literacy: essential skills for consumer health in a networked world. Journal of medical Internet research. 2006;8(2):e506.

49. Kuek A, Hakkennes S. Healthcare staff digital literacy levels and their attitudes towards information systems. Health informatics journal. 2020;26(1):592-612.

50. Saplacan D. Situated Abilities: Understanding Everyday Use of ICTs. 2020.

51. Saplacan D, Khaksar W, Torresen J, editors. On Ethical Challenges Raised by Care Robots: A Review of the Existing Regulatory-, Theoretical-, and Research Gaps. 2021 IEEE International Conference on Advanced Robotics and Its Social Impacts (ARSO); 2021: IEEE.
52. Bratteteig T, Saplacan D, Soma R, Svanes Oskarsen J, editors. Strengthening human autonomy in the era of autonomous technology. Contemporary perspectives on interaction with 'autonomous things'. Proceedings of the 11th Nordic Conference on Human-Computer Interaction: Shaping Experiences, Shaping Society; 2020.
53. Lim S, Tan A, Madden S, Hill B. Health professionals' and postpartum women's perspectives on digital health interventions for lifestyle management in the postpartum period: A systematic review of qualitative studies. Frontiers in endocrinology. 2019;10:767.
54. Balakrishnan R, Gopichandran V, Chaturvedi S, Chatterjee R, Mahapatra T, Chaudhuri I. Continuum of Care Services for Maternal and Child Health using mobile technology–a health system strengthening strategy in low and middle income countries. BMC medical informatics and decision making. 2016;16:1-8.
55. Hussain-Shamsy N, Shah A, Vigod SN, Zaheer J, Seto E. Mobile health for perinatal depression and anxiety: scoping review. Journal of medical Internet research. 2020;22(4):e17011.
56. Casiddu N, Cesta A, Cortellessa G, Orlandini A, Porfirione C, Divano A, et al., editors. Robot interface design: The giraff telepresence robot for social interaction. Ambient Assisted Living: Italian Forum 2014; 2015: Springer.
57. Coradeschi S, Cesta A, Cortellessa G, Coraci L, Galindo C, Gonzalez J, et al. GiraffPlus: a system for monitoring activities and physiological parameters and promoting social interaction for elderly. Human-Computer Systems Interaction: Backgrounds and Applications 3. 2014:261-71.
58. Orlandini A, Kristoffersson A, Almquist L, Björkman P, Cesta A, Cortellessa G, et al. Excite project: A review of forty-two months of robotic telepresence technology evolution. Presence: Teleoperators and Virtual Environments. 2016;25(3):204-21.
59. Pages J, Marchionni L, Ferro F, editors. Tiago: the modular robot that adapts to different research needs. International workshop on robot modularity, IROS; 2016.
60. Ake-Kob A, Blazeviciene A, Colonna L, Cartolovni A, Dantas C, Fedosov A, et al. State of the art on ethical, legal, and social issues linked to audio-and video-based AAL solutions. 2021.

Table 1. CASP assessment of included studies*

Study	Q1	Q2	Q3	Q4	Q5	Q6	Q7	Q8	Q9	Q10	Sum
Danbjørg et al. 2014 [24]	1	1	1	1	1	1	1	1	1	1	10
Danbjørg et al. 2014 [25]	1	1	1	1	1	1	1	1	1	1	10
Danbjørg et al. 2015	1	1	1	1	1	1	1	1	1	1	10
Garne Holm et al. 2019											10

Garne et al. 2016	1	1	1	1	1	1	1	1	1	1	10
Lindberg et al. 2009	1	1	1	1	1	1	1	1	1	1	10
Shorey et al. 2018	1	1	1	1	1	1	1	1	1	1	10
Shorey et al. 2019	1	1	1	1	1	1	1	1	1	1	10

*CASP Checklist (Yes=1; Can't tell=X; No=0)

10 questions to help you make sense of qualitative research: Q1 Was there a clear statement of the aims of the research? Q2 Is a qualitative methodology appropriate? Q3 Was the research design appropriate to address the aims of the research? Q4 Was the recruitment strategy appropriate to the aims of the research? Q5 Was the data collected in a way that addressed the research issue? Q6 Has the relationship between researcher and participants been adequately considered? Q7 Have ethical issues been taken into consideration? Q8 Was the data analysis sufficiently rigorous? Q9 Is there a clear statement of findings? Q10 How valuable is the research?

Table 2. Overview of included studies

**Author, year, country**	**Aim**	**Welfare technology used**	**Method design**	**Participants**	**Data collection**	**Data analysis**	**Results**
Danbjørg et al. 2015 Denmark	To explore how parents experienced the use of telemedicine following early postnatal discharge	Application	Participatory and qualitative design	27 parents & 11 partners	Interviews	Systematic text condensation	Parents were confident in using it natural to seek information an not experience any barriers i asynchronous communication. (i.e., chat, knowledge base and needs of the new parents and i parental self-efficacy.
Danbjørg et al. 2014 Denmark	To describe the process of the design, development, and testing of an application as a viable information technology solution	Application	Participatory design with in-depth consultation with users	9 parents & 1 father	Participant observation, field notes, individual telephone interviews	Systematic text condensation	The families found it natural to did not feel any barriers. The a for follow-up support. The testin and the nurses found the app refinements and wider testing.
Danbjørg et al. 2014 Denmark	To identify the nursing support needs of new parents and their infants during the first seven days postpartum, by drawing on the experiences of all stakeholders in early postnatal discharge from hospital, and thereby gaining new knowledge to investigate further whether telemedicine is a viable option in providing the required support	Telemedicine	Participatory and qualitative design	19 parents	Participant observation, interviews, workshop	Systematic text condensation	Families that were discharged e from the healthcare system to questions during the postnatal p to communicate that would individualised and timely info results indicate that it may be support needs of these fam communicate, such as online co
Garne et al. 2016 Denmark	To identify parents' needs when seeking to provide neonatal home care supported by telemedicine	Telemedicine	Participatory and qualitative design	19 parents	Observational studies, individual interviews, and focus group interviews	Systematic text condensation	This study supports the use of care because it gives parents th promotes their self-efficacy. Te gives parents the guidance they not require hands-on support. P a technological 'bell cord', such mail communication, and a k regarding infant nutrition and br
Garne Holm et al. 2019 Denmark	To explore parents' experiences with neonatal tele-homecare (NTH)	Application	Qualitative design	49 parents	Interviews	Systematic text condensation	The results from the study iden family-centred care (FCC). Th personal lifeline to clinical expe Through the videoconferenc acknowledged regarding thei observations of their infants. S homecare can be a supportive p
Linberg et al. 2009 Sweden	To describe parents' experiences of using videoconferencing when discharged early from a maternity unit	Videoconferencing	Combination of quantitative and qualitative design	9 couples	Questionnaires and interviews	Thematic content analysis	Study results showed that parent the technology, being face-to-f (VC), having control over thei especially 'when worries and c were received'. Using VC as a s after childbirth can facilitate a m to be guided by the midwife in t
Shorey et al. 2018 Singapore	To explore the views of parents of newborns with regard to the content and delivery of a mobile health (mHealth) app-based postnatal educational programme	Application	Qualitative design	17 participants (5 couples, 4 partners, and 3 parents)	Interviews	Thematic content analysis	Study participants reported that was a good source of informati context. The parents assessed communication platform, a mi advice. Belonging to a virtual gave the parents the feeling tha supported by healthcare professi
Shorey et al. 2019 Singapore	To examine the experiences and perceptions of participants in a supportive education parenting programme intervention study	Application	Qualitative design	16 parents (6 control and 10 intervention)	Interviews	Thematic content analysis	Parents from the intervention experiences with receiving suffi multifeatured, technology-base improving parental outcomes a

PRISMA flowchart

Table 3 Overview of themes and codes

**Theme**	**Flexible digital nursing support**	**Digital literacy**	**Safe parenting with digital support**	**Adequate substitute for physical meetings**
**Codes**	available asynchronous information<br><br>online chat is preferred<br><br>good alternative to refreshing old knowledge<br><br>reassuring to have access to the information one needs<br><br>digital/app literacy essential for successful use<br><br>availability of information<br><br>opportunity to get information repeated<br><br>opportunity to portion out information based on current needs<br><br>interactive communication and tutoring<br><br>new method of communicating with parents<br><br>internet infrastructure essential for adequate remote nursing<br><br>written information is preferred<br><br>greater opportunity for individualized information<br><br>feeling of closeness<br><br>to get full attention<br><br>to get the time they need<br><br>confirmatory<br><br>Timesaving<br><br>support weighs more than fear of private disturbances	Being surprised with early discharge<br><br>equal need for support regardless of primipara or multipara<br><br>technology integrated into everyday life<br><br>accustomed to using technology<br><br>digital supervision increases the sense of manageability<br><br>asymmetry in digital literacy between healthcare professionals and parents<br><br>Healthcare professionals do not have as good digital skills as their parents<br><br>liberating<br><br>interactive collaboration with the profession<br><br>becomes more confident in their own capacity and competence<br><br>have skills to use the app<br><br>teach health professionals digital skills<br><br>to be engaged<br><br>empowered parents<br><br>mutual knowledge exchange<br><br>feeling of being in control<br><br>feeling of being prepared and strong in their new role<br><br>uncertain in his new role<br><br>need for frequent support<br><br>do not want to be discharged early<br><br>do not feel welcome<br><br>Own networks as alternative support Seek information on your own<br><br>feeling of insufficient support	covers the need for emotional support<br><br>feeling of safety<br><br>it turned out better than feared<br><br>nice to know that you can handle yourself<br><br>positive to be able to communicate directly with the midwife<br><br>to be relaxed and natural in front of the camera<br><br>follows own routines and rules at home<br><br>feeling of safety<br><br>relaxed atmosphere<br><br>relaxed home environment<br><br>important with regular access to information<br><br>sense of control<br><br>easier to absorb information in a quiet home environment<br><br>digital lifeline<br><br>emotional support<br><br>direct and personalized communication<br><br>home environment	Shared experiences regarding restriction in the private area<br><br>good substitute for physical meeting<br><br>longer use of the technology desirable<br><br>camera makes it possible to compose oral communication<br><br>very good alternative to hospital stays<br><br>technology is the lifeline to professional support around the clock<br><br>brilliant lifeline with professional support<br><br>efficiently with automated responses<br><br>practical solutions that prevent unnecessary hospital visits<br><br>photos and videos replace hospital visits<br><br>more and more easily accessible health personnel via the app<br><br>feel free to contact if needed<br><br>dare to ask more questions via the app<br><br>modern technology: can use as oral communication, with videos, with pictures, with written questions<br><br>24 hours useful<br><br>good alternative to stay in hospital

	new communication method<br><br>information can be made available via SMS or chat<br><br>a page with frequently asked questions<br><br>useful and easy App<br><br>positive feedback important<br><br>app as advice source most credible<br><br>round-the-clock support<br><br>available support if needed<br><br>easy to absorb information<br><br>available information<br><br>to get positive confirmation<br><br>availability of necessary personal information, up-to-date, asynchronous, reminder many times<br><br>feedback and attention<br><br>available support and timesaving new communication method	questioning of advice given<br><br>comparing information<br><br>need for personalized information<br><br>knowledge asymmetry in ICT between new parents and care staff<br><br>Integration of App in daily life<br><br>Feeling of control and support<br><br>Flexibility<br><br>Less stressful /calm<br><br>Feel insecure<br><br>strengthens self-confidence<br><br>experience of self-confidence<br><br>can better understand the baby's behaviour<br><br>dare easier asked<br><br>share their experiences with other parents<br><br>more confident<br><br>help to find out your own problem-solving strategies<br><br>support with interactive collaboration<br><br>share experiences<br><br>empower self confidence<br><br>bonding and strengthening family members		

Figure 3 Overview themes a sub-theme

**Theme**	**Flexible digital support**	**Digital literacy**	**Safe parenting with digital**	**Adequate substitute for physical meetings**
**Sub-theme**	Availability of necessary personal information<br><br>Up-to-date, asynchronous reminders<br><br>Feedback and attention<br><br>Available support<br><br>Timesaving by the new communication method	Support with interactive collaboration (app etc)<br><br>Sharing experiences<br><br>Empowering self-confidence<br><br>Bonding and strengthening family members	Emotional support<br><br>Direct and personalised communication<br><br>Home environment	Modern communication technology<br><br>Communication complemented with videos, pictures, and text<br><br>24-hour accessible alternative support

Søkehistorikk pubmed

01.03.21

Søk 1: ((((((((((((((("new mother")) OR ("new parents")) OR (birth)) OR (Childbirth)) OR (primiparas)) OR (multiparas)) OR (parent)) OR ("new parent")) OR ("new parents")) OR ("home based postnatal care")) OR (postpartum)) OR ("postnatal care")) OR ("managed care")) OR ("Ambulatory care")) OR ("After care")) OR ("home visit")) OR ("home visits")

Antall: 1 032 717

Søk 2: (((((((("health system") OR (telemedicine)) OR ("Health services")) OR ("mobile health")) OR ("Health, mobile")) OR (mhealth)) OR (telehealth)) OR (ehealth)) OR (telecommunication)) OR (telenursing)

Antall: 687 119

Søk 3: ((((("early postnatal discharge") OR ("early discharge")) OR ("length of stay")) OR ("very early discharge")) OR ("early postpartum discharge")) OR ("early patient discharge")

Antall: 127 601

Søk 4: ((((("semistructured interview") OR ("semistructured interviews")) OR ("cross sectional")) OR ("content analysis")) OR (qualitative)) OR ("grounded theory")

Antall: 833 989

Søk 5: #1 AND #2 AND #3

(((((((((((((((((("new mother")) OR ("new parents")) OR (birth)) OR (Childbirth)) OR (primiparas)) OR (multiparas)) OR (parent)) OR ("new parent")) OR ("new parents")) OR ("home based postnatal care")) OR (postpartum)) OR ("postnatal care")) OR ("managed care")) OR ("Ambulatory care")) OR ("After care")) OR ("home visit")) OR ("home visits")) AND ((((((((("health system") OR (telemedicine)) OR ("Health services")) OR ("mobile health")) OR ("Health, mobile")) OR (mhealth)) OR (telehealth)) OR (ehealth)) OR (telecommunication)) OR (telenursing))) AND (((((("early postnatal discharge") OR ("early discharge")) OR ("length of stay")) OR ("very early discharge")) OR ("early postpartum discharge")) OR ("early patient discharge"))) AND (((((("semistructured interview") OR ("semistructured interviews")) OR ("cross sectional")) OR ("content analysis")) OR (qualitative)) OR ("grounded theory"))

Antall: 98

Søk 6: #5 AND English AND from 2015/1/1 - 2021/12/31

((((((((((((((((((("new mother")) OR ("new parents")) OR (birth)) OR (Childbirth)) OR (primiparas)) OR (multiparas)) OR (parent)) OR ("new parent")) OR ("new parents")) OR ("home based postnatal care")) OR (postpartum)) OR ("postnatal care")) OR ("managed care")) OR ("Ambulatory care")) OR ("After care")) OR ("home visit")) OR ("home visits")) AND ((((((((("health system") OR (telemedicine)) OR ("Health services")) OR ("mobile health")) OR ("Health, mobile")) OR (mhealth)) OR (telehealth)) OR (ehealth)) OR (telecommunication)) OR (telenursing))) AND (((((("early postnatal discharge") OR ("early discharge")) OR ("length of stay")) OR ("very early discharge")) OR ("early postpartum discharge")) OR ("early patient discharge"))) AND (((((("semistructured interview") OR ("semistructured interviews")) OR ("cross sectional")) OR ("content analysis")) OR (qualitative)) OR ("grounded theory")) Filters: English, from 2015/1/1 - 2021/12/31

Antall 47

Konsepttabell:

Nybakte foreldre/barselperiode	Telemedisin	Tidlig utskriving	Design + andre avgrensninger
«New mother»<br><br>«New parents»	«health system*»	«Early postnatal discharge»	«Semistructured interview»<br><br>«Semistructured interviews»
~~Patient*~~	**Telemedicine**	«Early discharge»	**Cross-sectional**
Birth<br><br>**Childbirth**	**health services**	«**Length of stay**»<br><br>Forslag: «~~Reduce length of stay~~»	Qualitative
**Primiparas**	«mobile health»	«very early discharge»	«content analysis»
	Health, mobile		Grounded theory
**Multiparas**	Mhealth	«Early postpartum discharge»	~~Nordic countries~~

**Postpartum**	**Telehealth**	«Early patient discharge»	5 siste årene
**Parent**	ehealth		
«New parent»<br>«New parents»	**Telecommunication**		
«home-based postnatal care»	**Telenursing**		
**«postnatal care»**			
**«managed care»**			
**«ambulatory care»**			
**«After care»**			
«home visit»<br>«home visits»			

AMED 04.03.21

Nybakte foreldre/barselperiode	Telemedisin	Tidlig utskriving	Design + andre avgrensninger
New mother*	~~health system*~~	~~«Early postnatal discharge»~~<br>~~0 treff~~	«Semistructured interview*»
~~Patient*~~	**Telemedicine**	~~«Early discharge»~~	Cross-sectional
Birth<br>Childbirth	~~«health service»~~	~~**«Length of stay»**~~	Qualitative

		~~Forslag: «Reduce length of stay»~~	
Primiparas	«mobile health»	~~«very early discharge»~~	«content analysis»
	«Health, mobile»		«Grounded theory»
Multiparas	Mhealth	~~«Early postpartum discharge»~~<br>~~0 treff~~	~~Nordic countries~~
Postpartum	Telehealth	~~«Early patient discharge»~~	5 siste årene
Parent	ehealth		
New parent*	Telecommunication		
~~home-based postnatal care (0 treff)~~	Telenursing		
postnatal care			
~~managed care~~			
**~~ambulatory care~~**			
**~~After care~~**			
«home visit*»			

Må fjerne generelle søkeord. Får mye treff som omhandler andre temaer. Se de som er strøket over.

Antall treff: 9

SØK 1 EMBASE 04.03.21
Search for: limit 32 to (english language and yr="2015 -Current")
Results: 11

Database: Embase <1980 to 2021 Week 08>
Search Strategy:
----
1 new mother*.mp. (2121)
2 birth.mp. or exp birth/ (454814)
3 exp childbirth/ or childbirth.mp. (69271)
4 primiparas.mp. or exp primipara/ (5300)
5 multiparas.mp. or exp multipara/ (8261)
6 postpartum.mp. (78472)
7 parent*.mp. or exp parent/ (722613)
8 new parent*.mp. (1269)
9 home-based postnatal care.mp. (13)
10 postnatal care.mp. or exp postnatal care/ (115501)
11 1 or 2 or 3 or 4 or 5 or 6 or 7 or 8 or 9 or 10 (1247455)
12 telemedicine.mp. or exp telemedicine/ (51648)
13 mobile health.mp. (6123)
14 health, mobile.mp. (137)
15 mhealth.mp. (5257)
16 telehealth.mp. or exp telehealth/ (55832)
17 ehealth.mp. or exp telehealth/ (56550)
18 telecommunication.mp. or exp telecommunication/ (81421)
19 telenursing.mp. or exp telenursing/ (427)
20 12 or 13 or 14 or 15 or 16 or 17 or 18 or 19 (92227)
21 exp semi structured interview/ or semistructured interview*.mp. (49045)
22 cross sectional.mp. or exp cross-sectional study/ (604192)
23 qualitative.mp. or qualitative research/ (344533)
24 content analysis.mp. or exp content analysis/ (38633)
25 grounded theory.mp. or exp grounded theory/ (15927)
26 21 or 22 or 23 or 24 or 25 (979109)
27 very early discharge.mp. (41)
28 exp hospital discharge/ (134931)
29 postnatal discharge.mp. (74)
30 length of stay.mp. or exp "length of stay"/ (217905)
31 27 or 28 or 29 or 30 (333334)

32 11 and 20 and 26 and 31 (20)
33 limit 32 to (english language and yr="2015 -Current") (11)
34 new mother*.mp. (2121)
35 birth.mp. or exp birth/ (454814)
36 exp childbirth/ or childbirth.mp. (69271)
37 primiparas.mp. or exp primipara/ (5300)
38 multiparas.mp. or exp multipara/ (8261)
39 postpartum.mp. (78472)
40 parent*.mp. or exp parent/ (722613)
41 new parent*.mp. (1269)
42 home-based postnatal care.mp. (13)
43 postnatal care.mp. or exp postnatal care/ (115501)
44 34 or 35 or 36 or 37 or 38 or 39 or 40 or 41 or 42 or 43 (1247455)
45 telemedicine.mp. or exp telemedicine/ (51648)
46 mobile health.mp. (6123)
47 health, mobile.mp. (137)
48 mhealth.mp. (5257)
49 telehealth.mp. or exp telehealth/ (55832)
50 ehealth.mp. or exp telehealth/ (56550)
51 telecommunication.mp. or exp telecommunication/ (81421)
52 telenursing.mp. or exp telenursing/ (427)
53 45 or 46 or 47 or 48 or 49 or 50 or 51 or 52 (92227)
54 exp semi structured interview/ or semistructured interview*.mp. (49045)
55 cross sectional.mp. or exp cross-sectional study/ (604192)
56 qualitative.mp. or qualitative research/ (344533)
57 content analysis.mp. or exp content analysis/ (38633)
58 grounded theory.mp. or exp grounded theory/ (15927)
59 54 or 55 or 56 or 57 or 58 (979109)
60 very early discharge.mp. (41)
61 exp hospital discharge/ (134931)
62 postnatal discharge.mp. (74)
63 length of stay.mp. or exp "length of stay"/ (217905)
64 60 or 61 or 62 or 63 (333334)
65 44 and 53 and 59 and 64 (20)
66 limit 65 to (english language and yr="2015 -Current") (11)

****

SØK 2 i Embase 04.03.21
Search for: limit 24 to (english language and yr="2015 -Current")

Results: 88

Database: Embase <1980 to 2021 Week 08>
Search Strategy:

----

1 new mother*.mp. (2121)
2 primiparas.mp. or exp primipara/ (5300)
3 multiparas.mp. or exp multipara/ (8261)
4 postpartum.mp. (78472)
5 new parent*.mp. (1269)
6 home based postnatal care.mp. (13)
7 postnatal care.mp. or exp postnatal care/ (115501)
8 1 or 2 or 3 or 4 or 5 or 6 or 7 (178349)
9 telemedicine.mp. or exp telemedicine/ (51648)
10 "mobile health".mp. (6123)
11 health, mobile.mp. (137)
12 mhealth.mp. (5257)
13 telehealth.mp. or exp telehealth/ (55832)
14 ehealth.mp. (4778)
15 telecommunication.mp. or exp telecommunication/ (81421)
16 telenursing.mp. or exp telenursing/ (427)
17 9 or 10 or 11 or 12 or 13 or 14 or 15 or 16 (92227)
18 exp semi structured interview/ or semistructured interview*.mp. (49045)
19 cross sectional.mp. or exp cross-sectional study/ (604192)
20 qualitative.mp. or exp qualitative research/ (344815)
21 content analysis.mp. or exp content analysis/ (38633)
22 grounded theory.mp. or exp grounded theory/ (15927)
23 18 or 19 or 20 or 21 or 22 (979373)
24 8 and 17 and 23 (112)
25 limit 24 to (english language and yr="2015 -Current") (88)

#	Query	Limiters/Expanders	Last Run Via	Results
S43	S15 AND S32 AND S38 AND S41	Limiters - Published Date: 20150101-20211231; English Language; Peer Reviewed<br>Expanders - Apply equivalent subjects<br>Search modes - Boolean/Phrase	Interface - EBSCOhost Research Databases<br>Search Screen - Advanced Search<br>Database - CINAHL with Full Text	35
S42	S15 AND S32 AND S38 AND S41	Expanders - Apply equivalent subjects<br>Search modes - Boolean/Phrase	Interface - EBSCOhost Research Databases<br>Search Screen - Advanced Search<br>Database - CINAHL with Full Text	38
S41	S24 OR S39 OR S40	Expanders - Apply equivalent subjects<br>Search modes - Boolean/Phrase	Interface - EBSCOhost Research Databases<br>Search Screen - Advanced Search<br>Database - CINAHL with Full Text	123,018
S40	"health service*"	Limiters - Published Date: 20150101-20211231; English Language; Peer Reviewed<br>Expanders - Apply equivalent subjects<br>Search modes - Boolean/Phrase	Interface - EBSCOhost Research Databases<br>Search Screen - Advanced Search<br>Database - CINAHL with Full Text	79,909

S39	"health system*"	Limiters - Published Date: 20150101-20211231; English Language; Peer Reviewed<br>Expanders - Apply equivalent subjects<br>Search modes - Boolean/Phrase	Interface - EBSCOhost Research Databases<br>Search Screen - Advanced Search<br>Database - CINAHL with Full Text	16,240

S38	S33 OR S34 OR S35 OR S36 OR S37	Expanders - Apply equivalent subjects<br>Search modes - Boolean/Phrase	Interface - EBSCOhost Research Databases<br>Search Screen - Advanced Search<br>Database - CINAHL with Full Text	440,777
S37	"grounded theor*"	Expanders - Apply equivalent subjects<br>Search modes - Boolean/Phrase	Interface - EBSCOhost Research Databases<br>Search Screen - Advanced Search<br>Database - CINAHL with Full Text	19,434
S36	"content analys*"	Expanders - Apply equivalent subjects<br>Search modes - Boolean/Phrase	Interface - EBSCOhost Research Databases<br>Search Screen - Advanced Search<br>Database - CINAHL with Full Text	45,885
S35	qualitative	Expanders - Apply equivalent subjects<br>Search modes - Boolean/Phrase	Interface - EBSCOhost Research Databases<br>Search Screen - Advanced Search<br>Database - CINAHL with Full Text	178,984
S34	"cross sectional"	Expanders - Apply equivalent subjects<br>Search modes - Boolean/Phrase	Interface - EBSCOhost Research Databases<br>Search Screen - Advanced Search<br>Database - CINAHL with Full Text	241,548

S33	semistructured interview*"	Expanders - Apply equivalent subjects<br>Search modes - Boolean/Phrase	Interface - EBSCOhost Research Databases<br>Search Screen - Advanced Search<br>Database - CINAHL with Full Text	7,764
S32	S25 OR S26 OR S27 OR S28 OR S29 OR S30 OR S31	Expanders - Apply equivalent subjects<br>Search modes - Boolean/Phrase	Interface - EBSCOhost Research Databases<br>Search Screen - Advanced Search<br>Database - CINAHL with Full Text	57,677

S31	"Early patient discharge"	Expanders - Apply equivalent subjects<br>Search modes - Boolean/Phrase	Interface - EBSCOhost Research Databases<br>Search Screen - Advanced Search<br>Database - CINAHL with Full Text	1,012
S30	"Early postpartum discharge"	Expanders - Apply equivalent subjects<br>Search modes - Boolean/Phrase	Interface - EBSCOhost Research Databases<br>Search Screen - Advanced Search<br>Database - CINAHL with Full Text	42
S29	"very early discharge"	Expanders - Apply equivalent subjects<br>Search modes - Boolean/Phrase	Interface - EBSCOhost Research Databases<br>Search Screen - Advanced Search<br>Database - CINAHL with Full Text	8
S28	"Reduce length of stay"	Expanders - Apply equivalent subjects<br>Search modes - Boolean/Phrase	Interface - EBSCOhost Research Databases<br>Search Screen - Advanced Search<br>Database - CINAHL with Full Text	217
S27	"length of stay"	Expanders - Apply equivalent subjects<br>Search modes - Boolean/Phrase	Interface - EBSCOhost Research Databases<br>Search Screen - Advanced Search<br>Database - CINAHL with Full Text	56,269

S26	"early discharge"	Expanders - Apply equivalent subjects<br>Search modes - Boolean/Phrase	Interface - EBSCOhost Research Databases<br>Search Screen - Advanced Search<br>Database - CINAHL with Full Text	1,693
S25	"early postnatal discharge"	Expanders - Apply equivalent subjects<br>Search modes - Boolean/Phrase	Interface - EBSCOhost Research Databases<br>Search Screen - Advanced Search<br>Database - CINAHL with Full Text	27

S24	S16 OR S17 OR S18 OR S19 OR S20 OR S21 OR S22 OR S23	Expanders - Apply equivalent subjects<br>Search modes - Boolean/Phrase	Interface - EBSCOhost Research Databases<br>Search Screen - Advanced Search<br>Database - CINAHL with Full Text	34,390
S23	Telenursing	Expanders - Apply equivalent subjects<br>Search modes - Boolean/Phrase	Interface - EBSCOhost Research Databases<br>Search Screen - Advanced Search<br>Database - CINAHL with Full Text	2,188
S22	telecommun*	Expanders - Apply equivalent subjects<br>Search modes - Boolean/Phrase	Interface - EBSCOhost Research Databases<br>Search Screen - Advanced Search<br>Database - CINAHL with Full Text	3,295
S21	ehealth	Expanders - Apply equivalent subjects<br>Search modes - Boolean/Phrase	Interface - EBSCOhost Research Databases<br>Search Screen - Advanced Search<br>Database - CINAHL with Full Text	17,212
S20	telehealth	Expanders - Apply equivalent subjects<br>Search modes - Boolean/Phrase	Interface - EBSCOhost Research Databases<br>Search Screen - Advanced Search<br>Database - CINAHL with Full Text	20,686

S19	mhealth	Expanders - Apply equivalent subjects<br>Search modes - Boolean/Phrase	Interface - EBSCOhost Research Databases<br>Search Screen - Advanced Search<br>Database - CINAHL with Full Text	16,640
S18	"Health, mobile"	Expanders - Apply equivalent subjects<br>Search modes - Boolean/Phrase	Interface - EBSCOhost Research Databases<br>Search Screen - Advanced Search<br>Database - CINAHL with Full Text	67

S17	"mobile health"	Expanders - Apply equivalent subjects<br>Search modes - Boolean/Phrase	Interface - EBSCOhost Research Databases<br>Search Screen - Advanced Search<br>Database - CINAHL with Full Text	18,886
S16	Telemedicine	Expanders - Apply equivalent subjects<br>Search modes - Boolean/Phrase	Interface - EBSCOhost Research Databases<br>Search Screen - Advanced Search<br>Database - CINAHL with Full Text	20,685
S15	S1 OR S2 OR S3 OR S4 OR S5 OR S6 OR S7 OR S8 OR S9 OR S10 OR S11 OR S12 OR S13 OR S14	Expanders - Apply equivalent subjects<br>Search modes - Boolean/Phrase	Interface - EBSCOhost Research Databases<br>Search Screen - Advanced Search<br>Database - CINAHL with Full Text	390,678
S14	"home visit*"	Expanders - Apply equivalent subjects<br>Search modes - Boolean/Phrase	Interface - EBSCOhost Research Databases<br>Search Screen - Advanced Search<br>Database - CINAHL with Full Text	9,884
S13	"after care"	Expanders - Apply equivalent subjects<br>Search modes - Boolean/Phrase	Interface - EBSCOhost Research Databases<br>Search Screen - Advanced Search<br>Database - CINAHL with Full Text	15,681

S12	"ambulatory care"	Expanders - Apply equivalent subjects<br>Search modes - Boolean/Phrase	Interface - EBSCOhost Research Databases<br>Search Screen - Advanced Search<br>Database - CINAHL with Full Text	23,994
S11	"managed care"	Expanders - Apply equivalent subjects<br>Search modes - Boolean/Phrase	Interface - EBSCOhost Research Databases<br>Search Screen - Advanced Search<br>Database - CINAHL with Full Text	16,183

S10	"postnatal care"	Expanders - Apply equivalent subjects<br>Search modes - Boolean/Phrase	Interface - EBSCOhost Research Databases<br>Search Screen - Advanced Search<br>Database - CINAHL with Full Text	6,251
S9	"home based postnatal care"	Expanders - Apply equivalent subjects<br>Search modes - Boolean/Phrase	Interface - EBSCOhost Research Databases<br>Search Screen - Advanced Search<br>Database - CINAHL with Full Text	11
S8	"new parent*"	Expanders - Apply equivalent subjects<br>Search modes - Boolean/Phrase	Interface - EBSCOhost Research Databases<br>Search Screen - Advanced Search<br>Database - CINAHL with Full Text	694
S7	parent*	Expanders - Apply equivalent subjects<br>Search modes - Boolean/Phrase	Interface - EBSCOhost Research Databases<br>Search Screen - Advanced Search<br>Database - CINAHL with Full Text	186,263
S6	postpartum	Expanders - Apply equivalent subjects<br>Search modes - Boolean/Phrase	Interface - EBSCOhost Research Databases<br>Search Screen - Advanced Search<br>Database - CINAHL with Full Text	28,956

S5	multipar*	Expanders - Apply equivalent subjects<br>Search modes - Boolean/Phrase	Interface - EBSCOhost Research Databases<br>Search Screen - Advanced Search<br>Database - CINAHL with Full Text	4,846
S4	primipara*	Expanders - Apply equivalent subjects<br>Search modes - Boolean/Phrase	Interface - EBSCOhost Research Databases<br>Search Screen - Advanced Search<br>Database - CINAHL with Full Text	2,097

S3	Childbirth	Expanders - Apply equivalent subjects Search modes - Boolean/Phrase	Interface - EBSCOhost Research Databases Search Screen - Advanced Search Database - CINAHL with Full	36,233
S2	Birth	Expanders - Apply equivalent subjects Search modes - Boolean/Phrase	Interface - EBSCOhost Research Databases Search Screen - Advanced Search Database - CINAHL with Full	117,861
S1	"new mother*"	Expanders - Apply equivalent subjects Search modes - Boolean/Phrase	Interface - EBSCOhost Research Databases Search Screen - Advanced Search Database - CINAHL with Full	1,705

Wednesday, March 03, 2021 8:02:15 AM

#	Query	Limiters/Expanders	Last Run Via	Results
S42	S15 AND S26 AND S34 AND S40	Limiters - Published Date: 20150101-20211231; Scholarly (Peer Reviewed) Journals; Language: English<br>Expanders - Apply equivalent subjects<br>Search modes - Boolean/Phrase	Interface - EBSCOhost Research Databases<br>Search Screen - Advanced Search<br>Database - Academic Search Elite	59
S41	S15 AND S26 AND S34 AND S40	Expanders - Apply equivalent subjects<br>Search modes - Boolean/Phrase	Interface - EBSCOhost Research Databases<br>Search Screen - Advanced Search<br>Database - Academic Search Elite	91
S40	S35 OR S36 OR S37 OR S38 OR S39	Expanders - Apply equivalent subjects<br>Search modes - Boolean/Phrase	Interface - EBSCOhost Research Databases<br>Search Screen - Advanced Search<br>Database - Academic Search Elite	606,999
S39	"grounded theory"	Expanders - Apply equivalent subjects<br>Search modes - Boolean/Phrase	Interface - EBSCOhost Research Databases<br>Search Screen - Advanced Search<br>Database - Academic Search Elite	15,273

S38	"content analysis"	Expanders - Apply equivalent subjects<br>Search modes - Boolean/Phrase	Interface - EBSCOhost Research Databases<br>Search Screen - Advanced Search<br>Database - Academic Search Elite	46,633
S37	qualitative	Expanders - Apply equivalent subjects	Interface - EBSCOhost Research Databases<br>Search Screen - Advanced	307,491

		Search modes - Boolean/Phrase	Search<br>Database - Academic Search Elite	
S36	"cross sectional"	Expanders - Apply equivalent subjects<br>Search modes - Boolean/Phrase	Interface - EBSCOhost Research Databases<br>Search Screen - Advanced Search<br>Database - Academic Search Elite	267,280
S35	"semistructured interview*"	Expanders - Apply equivalent subjects<br>Search modes - Boolean/Phrase	Interface - EBSCOhost Research Databases<br>Search Screen - Advanced Search<br>Database - Academic Search Elite	10,433
S34	S27 OR S28 OR S29 OR S30 OR S31 OR S32 OR S33	Expanders - Apply equivalent subjects<br>Search modes - Boolean/Phrase	Interface - EBSCOhost Research Databases<br>Search Screen - Advanced Search<br>Database - Academic Search Elite	38,693
S33	"Early patient discharge"	Expanders - Apply equivalent subjects<br>Search modes - Boolean/Phrase	Interface - EBSCOhost Research Databases<br>Search Screen - Advanced Search<br>Database - Academic Search Elite	14

S32	"Early postpartum discharge"	Expanders - Apply equivalent subjects<br>Search modes - Boolean/Phrase	Interface - EBSCOhost Research Databases<br>Search Screen - Advanced Search<br>Database - Academic Search Elite	31
S31	"very early discharge"	Expanders - Apply equivalent subjects<br>Search modes - Boolean/Phrase	Interface - EBSCOhost Research Databases<br>Search Screen - Advanced Search<br>Database - Academic Search Elite	10
S30	"Reduce length of stay"	Expanders - Apply equivalent subjects	Interface - EBSCOhost Research Databases<br>Search Screen - Advanced	156

		Search modes - Boolean/Phrase	Search<br>Database - Academic Search Elite	
S29	"length of stay"	Expanders - Apply equivalent subjects<br>Search modes - Boolean/Phrase	Interface - EBSCOhost Research Databases<br>Search Screen - Advanced Search<br>Database - Academic Search Elite	37,570
S28	"early discharge"	Expanders - Apply equivalent subjects<br>Search modes - Boolean/Phrase	Interface - EBSCOhost Research Databases<br>Search Screen - Advanced Search<br>Database - Academic Search Elite	1,376
S27	"early postnatal discharge"	Expanders - Apply equivalent subjects<br>Search modes - Boolean/Phrase	Interface - EBSCOhost Research Databases<br>Search Screen - Advanced Search<br>Database - Academic Search Elite	18
S26	S16 OR S17 OR S18 OR S19 OR S20 OR S21 OR S22 OR S23 OR S24 OR S25	Expanders - Apply equivalent subjects<br>Search modes - Boolean/Phrase	Interface - EBSCOhost Research Databases<br>Search Screen - Advanced Search<br>Database - Academic Search Elite	1,036,777

S25	telenursing	Expanders - Apply equivalent subjects<br>Search modes - Boolean/Phrase	Interface - EBSCOhost Research Databases<br>Search Screen - Advanced Search<br>Database - Academic Search Elite	337
S24	telecommunication	Expanders - Apply equivalent subjects<br>Search modes - Boolean/Phrase	Interface - EBSCOhost Research Databases<br>Search Screen - Advanced Search<br>Database - Academic Search Elite	370,174
S23	ehealth	Expanders - Apply equivalent subjects	Interface - EBSCOhost Research Databases<br>Search Screen - Advanced	15,739

		Search modes - Boolean/Phrase	Search<br>Database - Academic Search Elite	
S22	telehealth	Expanders - Apply equivalent subjects<br>Search modes - Boolean/Phrase	Interface - EBSCOhost Research Databases<br>Search Screen - Advanced Search<br>Database - Academic Search Elite	15,607
S21	mhealth	Expanders - Apply equivalent subjects<br>Search modes - Boolean/Phrase	Interface - EBSCOhost Research Databases<br>Search Screen - Advanced Search<br>Database - Academic Search Elite	15,801
S20	"health, mobile"	Expanders - Apply equivalent subjects<br>Search modes - Boolean/Phrase	Interface - EBSCOhost Research Databases<br>Search Screen - Advanced Search<br>Database - Academic Search Elite	84
S19	"mobile health"	Expanders - Apply equivalent subjects<br>Search modes - Boolean/Phrase	Interface - EBSCOhost Research Databases<br>Search Screen - Advanced Search<br>Database - Academic Search Elite	15,910

S18	Telemedicine	Expanders - Apply equivalent subjects<br>Search modes - Boolean/Phrase	Interface - EBSCOhost Research Databases<br>Search Screen - Advanced Search<br>Database - Academic Search Elite	20,104
S17	"health service*"	Expanders - Apply equivalent subjects<br>Search modes - Boolean/Phrase	Interface - EBSCOhost Research Databases<br>Search Screen - Advanced Search<br>Database - Academic Search Elite	582,890
S16	"health system*"	Expanders - Apply equivalent subjects	Interface - EBSCOhost Research Databases<br>Search Screen - Advanced	110,251

		Search modes - Boolean/Phrase	Search<br>Database - Academic Search Elite	
S15	S1 OR S2 OR S3 OR S4 OR S5 OR S6 OR S7 OR S8 OR S9 OR S10 OR S11 OR S12 OR S13 OR S14	Expanders - Apply equivalent subjects<br>Search modes - Boolean/Phrase	Interface - EBSCOhost Research Databases<br>Search Screen - Advanced Search<br>Database - Academic Search Elite	847,395
S14	"home visit*"	Expanders - Apply equivalent subjects<br>Search modes - Boolean/Phrase	Interface - EBSCOhost Research Databases<br>Search Screen - Advanced Search<br>Database - Academic Search Elite	15,677
S13	aftercare	Expanders - Apply equivalent subjects<br>Search modes - Boolean/Phrase	Interface - EBSCOhost Research Databases<br>Search Screen - Advanced Search<br>Database - Academic Search Elite	6,914
S12	"ambulatory care"	Expanders - Apply equivalent subjects<br>Search modes - Boolean/Phrase	Interface - EBSCOhost Research Databases<br>Search Screen - Advanced Search<br>Database - Academic Search Elite	20,405

S11	"managed care"	Expanders - Apply equivalent subjects<br>Search modes - Boolean/Phrase	Interface - EBSCOhost Research Databases<br>Search Screen - Advanced Search<br>Database - Academic Search Elite	18,632
S10	"postnatal care"	Expanders - Apply equivalent subjects<br>Search modes - Boolean/Phrase	Interface - EBSCOhost Research Databases<br>Search Screen - Advanced Search<br>Database - Academic Search Elite	5,141
S9	"home based postnatal care"	Expanders - Apply equivalent subjects	Interface - EBSCOhost Research Databases<br>Search Screen - Advanced	8

		Search modes - Boolean/Phrase	Search<br>Database - Academic Search Elite	
S8	"new parent*"	Expanders - Apply equivalent subjects<br>Search modes - Boolean/Phrase	Interface - EBSCOhost Research Databases<br>Search Screen - Advanced Search<br>Database - Academic Search Elite	1,186
S7	Parent*	Expanders - Apply equivalent subjects<br>Search modes - Boolean/Phrase	Interface - EBSCOhost Research Databases<br>Search Screen - Advanced Search<br>Database - Academic Search Elite	482,962
S6	postpartum	Expanders - Apply equivalent subjects<br>Search modes - Boolean/Phrase	Interface - EBSCOhost Research Databases<br>Search Screen - Advanced Search<br>Database - Academic Search Elite	39,044
S5	multiparas	Expanders - Apply equivalent subjects<br>Search modes - Boolean/Phrase	Interface - EBSCOhost Research Databases<br>Search Screen - Advanced Search<br>Database - Academic Search Elite	1,355

S4	primiparas	Expanders - Apply equivalent subjects<br>Search modes - Boolean/Phrase	Interface - EBSCOhost Research Databases<br>Search Screen - Advanced Search<br>Database - Academic Search Elite	1,283
S3	childbirth	Expanders - Apply equivalent subjects<br>Search modes - Boolean/Phrase	Interface - EBSCOhost Research Databases<br>Search Screen - Advanced Search<br>Database - Academic Search Elite	47,721
S2	birth	Expanders - Apply equivalent subjects	Interface - EBSCOhost Research Databases<br>Search Screen - Advanced	294,546

		Search modes - Boolean/Phrase	Search<br>Database - Academic Search Elite	
S1	"new mother*"	Expanders - Apply equivalent subjects<br>Search modes - Boolean/Phrase	Interface - EBSCOhost Research Databases<br>Search Screen - Advanced Search<br>Database - Academic Search	1,956

Figure
Click here to access/download;Figure;PRISMA flowchart of study selection procedure.tif

PRISMA flowchart of study selection procedure

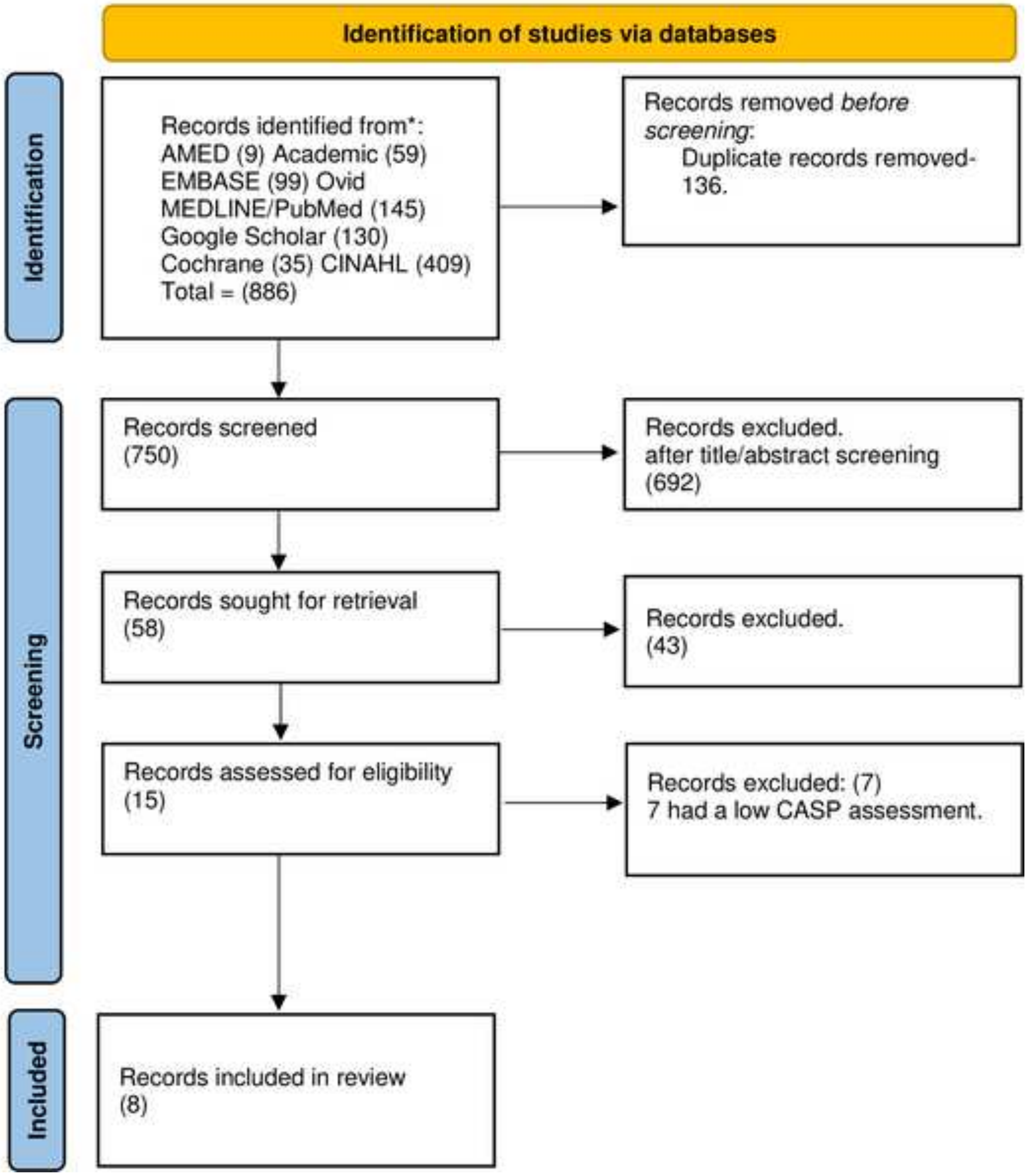

Formatted: Numbering: Continuous

Digital solutions to follow up on discharged new parents - a systematic literature review

Author names:

Zada Pajalic[1], Alona Rauckiene[2] Grethe Savosnick-, Irena Bartels[3] , Jean Calleja Agius[4], Diana Saplacan5, Sigríður Sía Jónsdóttir[6] & Babak Asadi-Azarbaijani[1]

Affiliations

[1]Faculty of Health Sciences, VID Specialized University, Norway

[2] Department of Health Research and Innovation Science Centre, Klaipeda University, Lithuania

[3]East Tallinn Central Hospital, Estonia

[4]Department of Anatomy, Faculty of Medicine and Surgery, University of Malta, Malta

[5]Department of Informatics, University of Oslo, Norway

[6]School of Health Science, University of Akureyri, Iceland

Corresponding author

Zada Pajalic

Zada.pajalic@vid.no

Digital solutions to follow up on discharged new parents

# - a systematic literature review

Abstract

New parents and their newborns are followed up after discharge either through home visits from midwives/nurses or using information and communication technology. This follow-up focuses on individual needs related to breastfeeding and infant feeding, practical advice on caring for babies, supporting and strengthening the new mother's knowledge and self-confidence concerning child development and parenting skills, and supporting the relationship between ~~mother~~ parents and baby. This systematic review aims to integrate available research results that describe new parents' ~~perceived sense of security~~experiences when health and care providers used telemedicine as a platform for follow-up after discharge from the childbirth department. This literature review was conducted following the PRISMA statement and was prospectively registered in PROSPERO CRD42021236912. The studies were identified through the following databases: AMED, Academic, EMBASE, Google Scholar, Ovid MEDLINE via PubMed, Cochrane database and CINAHL. Results from these studies were compiled using thematic analysis. A total of 886 studies were identified. Screening resulted in eight studies that met the inclusion criteria. Thematic analysis produced the following themes: a) ***Flexibility and convenience of digital support~~flexible digital support~~***, b) D~~d~~igital literacy, c) ***Parents feeling safe with digital support*** ~~safe parenting with digital support~~, and d) A~~a~~dequate substitute for physical meetings.

New parents who live in a home environment with a relaxed atmosphere and around-the-clock digital support experience a sense of control, security, full attention, and encouragement. Digital follow up at home has proven effective because it can meet the support needs of new parents when necessary.

Formatted: Font: (Default) Times New Roman, 12 pt, Bold, Italic

Formatted: Font: Bold, Italic

Keywords: Digital support, Ambient-Assisted Living, eHealth, telemedicine, telehealth, new parents, postpartum period, patient discharge

Formatted: Strikethrough

1.0 Introduction

Parity, birth-related factors, and psychosocial predictors factors indicate when it is appropriate for a new mother to be discharged and involved in the organisation of to postnatal care.

Discharge is when a healthy mother and baby return home from hospital after a birth [1]. Parity, birth-related factors, and psychosocial predictors indicate when a new mother can be discharged and involved in the organisation of postnatal care [2]. ~~Affinity~~ Family bonding, which is vital for the whole family, can be achieved more rapidly if the family is together at home and if ~~fathers~~partners are involved from birth [3].

Potential advantages of early discharge for new ~~mothers~~parents and their babies include a peaceful home environment, positive impact on breastfeeding, enhanced emotional well-being, minimised exposure to infection risks, and increased parental relaxation and involvement in child care [4]. Follow-up of new parents after discharge is usually offered by the midwife [5] via home visits [6] or telemedicine [7]. This follow-up often focuses on individual needs related to breastfeeding and infant feeding, practical advice on caring for babies, supporting and strengthening the new mother's knowledge and self-confidence, child development, parenting skills, and supporting the mother–child relationship [8].

Telemedicine is an umbrella term for the use of telecommunications technology to provide medical support at a distance [9]. Telemedicine is divided into different categories based on the interaction between users and the type of information used. The interaction can take place in real-time (synchronous) or be pre-recorded (asynchronous), and the information can be transmitted between users via audio, text, images, and videos [9]. A similar concept to telemedicine is eHealth, a term that comprises health service delivery between healthcare professionals and patients through the internet and related technologies [10]. Telemedicine

Formatted: Font: (Default) Times New Roman, 12 pt

Formatted: Plain Text, Left

Formatted: Font: 12 pt

Formatted: Font: 12 pt

Formatted: English (United Kingdom)

Formatted: English (United Kingdom)

Formatted: Font: 12 pt

offers new possibilities and enables support for many patients remotely in a cost-effective way [11].

There is an international trend in welfare countries ~~with social welfare policies~~ to shorten the postpartum length of stay in hospitals, driven by cost containment and hospital bed availability [12]. The average postpartum stay after vaginal delivery varies between countries, ~~but is about two days or fewer~~approximately [1]. Discharge after childbirth can be challenging for new ~~mothers~~parents, many of whom have a great need for practical support and expert guidance. As such, early discharge requires integrated care services, 24-hour availability of qualified healthcare professionals for advice and support, home visits and the opportunity for home support from qualified healthcare professionals [13]. Moreover, feeling safe following an early return home depends on whether ~~mothers~~parents make that decision themselves and whether breastfeeding has commenced [14]. Research shows that ~~mothers~~parents perceive discharge as positive when they receive adequate follow-up and help from healthcare professionals and know who to contact if needed [13] . Parents experience early discharge after birth as successful if they a) receive follow-up from a midwife and home visits after they return home, with the opportunity to obtain answers to questions when they arise; b) receive sufficient information; and c) are able to form a trusting relationship with the midwife [15].

Postpartum care must be based on a woman's decisions, clinical indications, family preferences, and care context [16]. Planned follow-up care has a significant impact on the health and well-being of ~~mothers~~parents, children, and families—both in the short and the long term [17, 18]. Research shows that home-based postnatal care is well accepted by new ~~mothers~~parents who are discharged after childbirth [19]. Moreover, when ~~mothers~~parents and ~~fathers~~partners feel empowered by their midwife, this enhances feelings of self-confidence and a sense of security after discharge from a maternity unit. All postpartum interventions related to the provision of ~~continuous~~continuity of care (quality of care over time) are associated with satisfaction and

impact the family's psychological and social outcomes [16]. With this review, we want to increase our knowledge of new parents' subjective perceptions, evaluations and interpretations regarding digital follow-up from the care provider's side after discharge from the maternity ward. The new understanding will give us insight into what is perceived to be working well and what challenges remain.

~~Aim~~

"This systematic review aims to integrate available research results that describe parents' ~~perceived sense of security~~experiences when health and care providers used telemedicine as a platform for follow-up after discharge from the childbirth department".

2.0 Methods

The systematic review was performed following the PRISMA statement and was prospectively registered in PROSPERO [20] (no. CRD42021236912).

2.1 Data sources and search strategy

The PROSPERO database was searched to determine whether similar studies had been completed or were still in progress. We could not find any registered studies with the same aim as ours.

Formatted: Font: 12 pt

2.2 Eligibility criteria

The review aimed to search for peer-reviewed primary qualitative studies that responded to the study's objective. Studies with a qualitative design describe parents' experiences, which answers the purpose of the study. The searches were performed between 030121-311221. A new search was carried out in October 2022. No new studies meeting the inclusion criteria were found.

2.3 Inclusion criteria

- All types of telemedicine used by healthcare professionals to follow-up discharged new parents from the childbirth department
- New parents discharged to their own homes
- Studies focusing on support in private homes
- Various ways that technology may be administrated, such as internet infrastructure implementation and individual provision
- Qualitative studies
- Welfare countries

2.4 Exclusion criteria

- **usual medical follow-up (e.g. return visits to hospital, home visits, follow-up at care centers)**
- Not target population
- Not primary study
- Protocol, review articles
- Books
- Studies in hospitals
- Quantitative studies

Formatted: Font: Bold

2.5 Information sources

At VID Specialised University in Oslo, Norway, a science librarian and first author (ZP) identified primary studies in the following bibliographic electronic databases: AMED, Academic, EMBASE, Ovid MEDLINE via PubMed, Google Scholar, Cochrane database and CINAHL.

2.6 Search strategy

Systematic searches were conducted for studies published in English, Norwegian, and Swedish. The searches were limited to the period between 2015 and 2022 to include the newest studies. The search strategy incorporated the following MeSH keywords singly and in combination with each other (combined with the Boolean operators OR and AND): patient, birth, early, discharge, postpartum, experience, length of stay, parent, semi-structured interviews, cross-sectional, sense of security, follow-up, health system, primipara, multipara, home-based postnatal care, midwife, home visits, ambulatory care, health service, managed care, aftercare, self-care, fear of infection, telemedicine, expectations.

2.7 Study selection and review process

In total, 886 studies were identified through literature searches and were first imported into the EndNote reference manager [21] and then into Rayyan [22] (a web tool for systematic reviews that aids in the comparison of decisions regarding inclusion or exclusion of studies). All duplicates (n=136) were removed resulting in 750 publications. Titles and abstracts for the 750 publications were then screened according to inclusion and exclusion criteria. Of these, 58 were screened in full text, 15 of which were assessed for quality using the Critical Appraisal Skills Programme (CASP) [23] checklist for qualitative studies. CASP assessment ranges from 0 -10. Ten points mean that the study is of high quality. Of these 15 publications, 8 [24-31] were judged to have the best combination of relevance and quality (see PRISMA Flow chart and Tables 1 and 2).

Screening of titles and abstracts was performed by all co-authors, first independently and then together. In the case of conflicting independent decisions, the authors reviewed the titles again together and discussed them to reach consensus. Full text screening and CASP quality review

were performed by all authors. The final decision of which studies to include was made by all reviewers.

2.8 Data synthesis

We chose to compile results from the selected articles using thematic synthesis, following Braun and Clarke [32]. Analysis of selected study results was performed by BAA and ZP and discussed with all participants until consensus was reached.

The first phase of our work was to extract the results from the selected studies in a separate document. After reading the text several times, we identified initial codes, which we then grouped into different themes. All co-authors critically reviewed the potential themes using a process of dialogue and consensus. Finally, we defined and named the following themes: a) flexible digital support, b) digital literacy, c) safe parenting with digital support, and d) adequate substitute for physical meetings (Table 3 and Figure 3).

3.0 Results

3.1 Flexibility and convenience of digital support~~Flexible digital support~~

Formatted: Font: (Default) Times New Roman, 12 pt, Bold, Italic

This study showed that the sharing of information between parents and healthcare professionals was essential [24-31]. Various digital platforms provided a combination of asynchronous (chat bots) and synchronous (staff respond directly via audio or video) methods to facilitate communication with parents out of hospital. Asynchronous communication offered an accessible way to obtain help after early discharge from maternity units. New parents appreciated having this information available, as well as an online chat option. This was also an excellent way to refresh the knowledge of those who already had children. Most parents found the new communication methods for contacting healthcare professionals easy to use and that they conveyed a range of information effectively, thus saving time. All parents felt safe

because they had access to the information they needed. It should be noted, however, that in terms of parents' ability to find and retrieve information, digital literacy was crucial.

This review also identified 24-hour access to information and the ability to access the same information repeatedly as additional positive aspects. Digital platforms provided the opportunity to distribute information based on current needs so that even new parents could share their knowledge and experiences via chat functions. Written information proved to be easily accessible around the clock, whether through an application (app), chat, or short text messages. Some groups also had access to web pages containing answers to the most frequently asked questions. Digital platforms created and led by midwives were perceived as the most credible sources for parents. Being able to receive rapid answers to questions that arose throughout the day or night made several new parents feel closer to their midwives and that they were being given the midwives' full attention whenever they needed it. Access to information thus gave parents a sense of control, security, and reassurance.

In addition, it was important for most new parents to receive positive feedback and confirmation from healthcare professionals. The flexibility that digital communication with healthcare professionals offered gave the parents a feeling of control and peace. New parents also stated that it was easier and more convenient to communicate digitally, and that sending a message was less disruptive. Some participants preferred asynchronous video instruction rather than face-to-face guidance from healthcare professionals because they could watch the videos whenever they had time and view them repeatedly if desired. In one of the studies, the visual contact established via videoconference facilitated a warm relationship between parents and healthcare professionals. The parents could see the nurses acknowledging their facial expressions and body language during their conversations when parents shared their observations related to their babies with the nurses. In this way, parents felt they were receiving the full attention of the healthcare professionals.

3.2 Digital literacy

Another theme that emerged was digital literacy among parents. Some study participants expressed that they were both surprised by and unprepared for early discharge after delivery; indeed, some had negative experiences because of this and had felt unwelcome [24-31]. Many of the new parents in the studies would have chosen to stay longer in hospital, with the main reasons being the need for breastfeeding support and help interpreting newborns' signals. However, the ability to use digital technology once they arrived home enhanced parents' feelings of security and gave them a sense of control.

All of the parents in the studies rapidly oriented themselves by using the media offered by the hospital as a support at home. Digital platforms with direct or asynchronous communication provided when needed reduced parents' feelings of insecurity about their new role. Asynchronous provision of information also helped meet the need for more frequent support. In addition, new parents reported using digital platforms (e.g. social media) as an alternative support strategy, seeking information on their own if they felt they were receiving insufficient support, were uncertain about something, or questioned the advice given. Some participants even wanted to compare the information they were receiving.

The most pressing need was to have more accessible personalised information on digital platforms. Every task that the parents accomplished via digital support strengthened their self-confidence and helped create a positive experience. Their self-confidence grew alongside their increased understanding of their baby's behaviour. Another freedom that the parents discovered with digital support was that they could ask any questions they desired, without feeling ashamed, which they found liberating. In step with increased self-confidence, some new parents also chose to share their experiences with other parents through this platform because they felt confident.

One interesting finding is that, in some cases, there was a mismatch in digital competence between healthcare professionals and parents, with healthcare professionals being at a disadvantage. When it became evident that the parents had better digital skills than the midwives, this led to a disparity in knowledge of how to navigate digital platforms between new parents and healthcare professionals. Some parents appreciated that they could help the midwives improve their digital competence via interactive collaboration; they felt confident regarding their capacity and ability to use the app or improve healthcare professionals' digital skills. By establishing some control, in addition to helping them feel prepared and strong in their new role, this kind of mutual knowledge exchange became a way to further empower parents.

3.3 ~~Safe~~ P~~p~~arents~~ing~~feeling safe with digital support

The results showed that digital follow-up met the parents' need for emotional support and gave them a sense of security [24-31]. Several parents reported that the digital follow-up was better than they expected to be. It was essential for participants to know that they could manage everything themselves and feel safe in their new role as parents.

For the parents, the most positive aspect was being able to communicate directly with the healthcare professionals and feeling comfortable and natural in front of the web camera. This contributed to a sense of control and made it easier to absorb information. Many likened the digital follow-ups at home to a digital lifeline. Indeed, new parents who could obtain the information they needed at home reported improved emotional well-being. They felt safe and relaxed, found it easier to seek help, and had more self-confidence when performing routine infant care tasks. It was important for parents to feel comfortable and supported with clarifying information whenever uncertainty arose.

New parents were positive about several aspects of the home environment. Many of them found it more comfortable to stay in their home environment, as long as it had a relaxed atmosphere. They felt that they had more control and could more easily follow their routines and roles in a quiet home environment compared to when they were in hospital. New parents who felt prepared for early discharge after giving birth expressed that a quiet home environment helped them to relate better to their child. Others reported that being at home enabled them to establish a circadian rhythm around the care of their newborn baby. They were also able to learn the baby's signals at their own pace. Moreover, at home, the parental roles became more apparent, as did the feeling of being a family, with the opportunity to focus on each other's needs and support each other. Many reported an increase in self-confidence and feelings of competence. In summary, a home environment with access to digital support when needed helped parents feel that they could safely handle their new role.

3.4 Adequate substitute for physical meetings

As noted earlier, several new parents shared their experiences with other parents, and digital meetings were a good substitute for physical meetings [24-31]. In some studies, the new parents had limited access to digital support and most of them wanted more. Conversations via webcam helped participants to communicate more effectively because the camera enabled midwives to see what parents were talking about and thus understand the issues at hand more efficiently than when they were only described orally. After trying out various digital support systems, most new parents concluded that these were better alternatives than staying longer in hospital. As mentioned above, the majority expressed that technology was a lifeline to professional support around the clock. Some felt that even automated answers constituted a good form of support, especially those that suggested practical solutions; these helped parents avoid unnecessary hospital visits. Parents also reported having better access to healthcare professionals through

the app than when they were in hospital. Many also found it easier to ask more questions in the app than in face-to-face meetings.

4.0 Discussion

The results from the present study highlight the new parents' experience based on ~~importance of~~ sustainable and flexible digital support for parents discharged early after childbirth [24-31]. Such support is especially important for new parents, who may feel that they lack confidence in their ability to care for a newborn. Our findings are supported by Wilson et al.'s study, which highlights the healthcare sector's suitability for digital follow-up of discharged patients. The healthcare sector can improve health outcomes using digital support for patients. Digital solutions make it possible distribution of resources efficiently and fairly. Digital health interventions are context-specific and a constantly changing process. [33]. Digital follow-up makes it possible to reach a greater number of people, provide increased service coverage, and reduce healthcare costs. Indeed, Awad et al. argue that digitised support for patients in the home environment is one of the most promising developments in modern care when the technology is strategic, well thought out, and adapted to individual needs [34]. Moreover, digital devices enable rapid diagnosis, individualised treatment, and interventions such as precision surgery, symptom monitoring counselling, rehabilitation, or targeted drug delivery. An excellent example of a focus on digital support is~~In~~ in medical logistic ares~~,~~ drones that can deliver necessary treatments to remote areas, collect samples, and even provide emergency assistance [11]. I~~ And i~~n maternity care, telemedicine platforms have been shown to be clinically effective and to improve patient satisfaction [35].

Th~~e present study~~ is review indicated that new parents were empowered when they had more knowledge ~~of and facility with~~regarding digital platforms than the health professionals [24-31]. This is supported by Alsem et al., who found improved parental satisfaction when parents used

digital tools to prepare for consultations with healthcare professionals [36]. Alsem et al. report positive experiences of personal and interactional factors determining empowerment, creating conditions for an equal doctor–parent relationship [36]. Moreover, while they found that digital tools proved helpful for parents to explore their needs and find information, they conclude that more research is needed to support autonomy in consultation. Salonen et al. highlight that the new generation of patients are accustomed to using the latest digital media and technological innovations [37-39]. These patients find that healthcare professionals are not always well-trained in using digital platforms [40]. This finding supports the creation of more technological solutions that meet the needs of ~~mothers~~parents in their first year postpartum [40-42].

Parents living in remote areas or lacking the support of relatives or friends are often reliant on telemedicine as a substitute for physical meetings [8]. As the COVID-19 pandemic has heightened concerns regarding infection, this has led to early hospital discharge and parental hesitancy towards in-person hospital or health centre visits and being physically seen by a healthcare professional, increasing the need for virtual consultations [15, 43, 44]. These have proven especially vital during lockdowns and in instances where parents have COVID-19 [45]. It was shown that the expertise obtained from existing telemedicine facilities operating in remote and rural areas has helped to shape digital follow-up after postnatal discharge [46].

Reddy et al. review has shown that most new parents have digital literacy, likely driven by the internet and the rapid increase in information and communication technology [47]. Around the world, digital health interventions are increasingly being adopted to address various public health issues. However, for users to understand, process, and act on health-related information, six literacy skills are necessary (according to the eHealth competence model): traditional, health, information, science, media, and computer literacy [48]. In the studies we reviewed, despite parents' willingness to support healthcare professionals in using digital solutions, healthcare professionals nevertheless required training in this new method of patient

communication. Results from a study by Kuek and Hakkennes [49] indicate that healthcare professionals who had low digital literacy levels experienced anxiety using digital technology. This situation affected the quality of care the patients were offered. Thus, any new digital solutions should first be tested in properly designed trials to assess the level of training needed both by the healthcare professionals and the beneficiary [49].

The present review also shows that while digital solutions can empower parents newly discharged from the hospital, they can also change how care is provided. A previous study discusses how the concept of care changes when, for instance, digital support is introduced as part of the care-giving process [50, 51]. Although the digital solutions explored in this review are mainly limited to apps and videoconferencing. It seems that digital solutions may represent a compromise between having a home visit from a healthcare professional and receiving no help whatsoever. However, the parents' autonomy, and whether they are empowered by digital tools, should be contextualised [52].

Our review has shown that digital solutions seems to benefit the follow-up of new parents after postnatal discharge, including by giving them 24-hour support. The digital solutions examined were mainly mobile applications, such as the mobile health app (mHealth) for postpartum care and other telemedicine solutions (e.g., videoconferencing) [53]. In addition, mHealth interventions have been shown to be effective in improving maternal and neonatal service utilisation [41, 54]. These digital solutions appear to serve their purpose when care receivers wish to retrieve information by themselves or need to get in touch with healthcare professionals. In addition, the solutions seem free of potential ethical dilemmas, such as those entailed by videoconferencing solutions which can be experienced as an intrusion of privacy due to the use of cameras.

Upon discharge from hospital, new parents – even those with high digital literacy – may find it challenging or overwhelming to use digital tools whilst also caring for a newborn. Indeed, telemedicine has proven to be important for women with low socioeconomic status, particularly in relation to addressing postnatal mental health [55].

In the future, more advanced platforms or technical solutions will be available, such as social and assistive robots. Robots could be used to assist with practical tasks within the home (e.g., cleaning, and collecting and transporting items within the home) and for eHealth purposes (e.g., getting in touch with a healthcare professional or giving advice to new parents). Such robots have been tested with older adults [56-58] or other Social and Assistive Robots (SARs) [59]. However, using SARs to support new parents following discharge remains understudied and represents an area for further research. Newly discharged parents often need help with practical tasks around the home so they can focus on their newborn; some of these can potentially already be allocated to robots [50] as they do not involve direct, intimate parent–robot interaction. On the other hand, tasks that involve more interaction between the robot and the newly discharged parents, like moving and bringing items, giving advice, and helping parents get in touch with healthcare professionals, may be interesting to investigate further. However, privacy, safety, and security issues may arise depending on how advanced the robot is and what equipment is used (e.g., cameras and sensors) [60]. Rigorous pilot testing and proper clinical trials are therefore needed before these digital applications are offered to new parents discharged early from hospital.

Implications for practice

This study's results are based on included studies with only four primary authors. This review indicates that telemedicine support for new parents has some potential worth investigating in more extensive RCT studies.~~After early home discharge, new parents should have~~

Conclusion

Personal counselling is an important component of care in the postpartum period, when new parents have issues specific to the child or mother (including postpartum mental health). A range of digital solutions are suitable in this situation, but a key feature of all such solutions is 24-hour access to information. In a home environment, digital communication gives parents a sense of security and emotional satisfaction; in contrast, problems can arise if parents lack access to digital resources after early discharge. The digital follow-up may be a great asset for new parents, as it can help to reliably inform them and give them the necessary support.

Declarations

Ethics approval and consent to participate

Not applicable

Consent for publication

Not applicable

Data availability statement

All data generated or analysed during this study are included in this published article and additional files including articles included in the analysis which are cited in the reference list.

Competing interests

The authors have declared that no competing interests exist.

Funding

Not applicable

Author Contributions

Conceptualisation: Zada Pajalic

Registration and final reporting in PROSPERO: Zada Pajalic

Searching: Zada Pajalic and Pinar Ekitz

Methodology: Zada Pajalic.

Data analysis: Zada Pajalic and Babak Asadi-Azarbaijani

Writing – original draft: Zada Pajalic, Babak Asadi-Azarbaijani, Alona Rauckiene, Grethe Savosnick, Irena Bartels, Jean Calleja Agius, Diana Saplacan, Sigríður Sía Jónsdóttir

Writing – review & editing: Zada Pajalic, Babak Asadi-Azarbaijani, Alona Rauckiene, Grethe Savosnick, Irena Bartels, Jean Calleja Agius, Diana Saplacan, Sigríður Sía Jónsdóttir

Corresponding authors: Zada Pajalic and Babak Asadi-Azarbaijani

References

1. Campbell OM, Cegolon L, Macleod D, Benova L. Length of stay after childbirth in 92 countries and associated factors in 30 low-and middle-income countries: compilation of reported data and a cross-sectional analysis from nationally representative surveys. PLoS medicine. 2016;13(3):e1001972.
2. Nilsson I, Danbjørg DB, Aagaard H, Strandberg-Larsen K, Clemensen J, Kronborg H. Parental experiences of early postnatal discharge: A meta-synthesis. Midwifery. 2015;31(10):926-34.
3. Persson EK, Dykes A-K. Parents' experience of early discharge from hospital after birth in Sweden. Midwifery. 2002;18(1):53-60.
4. Jones E, Stewart F, Taylor B, Davis PG, Brown SJ. Early postnatal discharge from hospital for healthy mothers and term infants. Cochrane Database of Systematic Reviews. 2021;(6).
5. Pajalic Z, Pajalic O, Saplacan D. Women's education and profession midwifery in Nordic countries. Journal of Health Sciences. 2019;9(3):127-35.
6. Roisné J, Delattre M, Rousseau S, Bourlet A, Charkaluk M-L. Newborn follow-up after discharge from the maternity unit: Compliance with national guidelines. Archives de Pédiatrie. 2018;25(2):95-9.
7. Filchev R, Pavlova D, Dimova R, Dovramadjiev T, editors. Healthcare System Sustainability by Application of Advanced Technologies in Telemedicine and eHealth. Human Interaction, Emerging Technologies and Future Systems V: Proceedings of the 5th International Virtual Conference on Human Interaction and Emerging Technologies, IHIET 2021, August 27-29, 2021 and the 6th IHIET: Future Systems (IHIET-FS 2021), October 28-30, 2021, France; 2022: Springer.
8. Lemyre B, Jefferies AL, O'Flaherty P. Facilitating discharge from hospital of the healthy term infant. Paediatrics & child health. 2018;23(8):515-22.
9. Wootton R, Craig J, Patterson V. Introduction to telemedicine: CRC Press; 2017.
10. Williatte-Pellitteri L. New Technologies, Telemedicine, eHealth, Data... What Are You Talking About? The Lawyer's Point of View. Digital Medicine. 2019:93-102.
11. Barnard-Kelly K. Utilizing eHealth and telemedicine technologies to enhance access and quality of consultations: it's not what you say, it's the way you say it. Diabetes Technology & Therapeutics. 2019;21(S2):S2-41-S2-7.
12. Benahmed N, San Miguel L, Devos C, Fairon N, Christiaens W. Vaginal delivery: how does early hospital discharge affect mother and child outcomes? A systematic literature review. BMC pregnancy and childbirth. 2017;17(1):1-14.
13. Kurth E, Krähenbühl K, Eicher M, Rodmann S, Fölmli L, Conzelmann C, et al. Safe start at home: what parents of newborns need after early discharge from hospital–a focus group study. BMC health services research. 2016;16(1):1-14.
14. Askelsdottir B, Lam-de Jonge W, Edman G, Wiklund I. Home care after early discharge: Impact on healthy mothers and newborns. Midwifery. 2013;29(8):927-34.
15. Barimani M, Vikström A. Successful early postpartum support linked to management, informational, and relational continuity. Midwifery. 2015;31(8):811-7.
16. Crowther S, MacIver E, Lau A. Policy, evidence and practice for post-birth care plans: a scoping review. BMC Pregnancy and Childbirth. 2019;19(1):1-16.
17. Olds DL, Kitzman H, Anson E, Smith JA, Knudtson MD, Miller T, et al. Prenatal and infancy nurse home visiting effects on mothers: 18-year follow-up of a randomized trial. Pediatrics. 2019;144(6).

18. Van Den Heuvel JF, Groenhof TK, Veerbeek JH, Van Solinge WW, Lely AT, Franx A, et al. eHealth as the next-generation perinatal care: an overview of the literature. Journal of medical Internet research. 2018;20(6):e202.
19. Johansson M, Thies-Lagergren L, Wells MB. Mothers experiences in relation to a new Swedish postnatal home-based model of midwifery care–A cross-sectional study. Midwifery. 2019;78:140-9.
20. Page MJ, McKenzie JE, Bossuyt PM, Boutron I, Hoffmann TC, Mulrow CD, et al. The PRISMA 2020 statement: an updated guideline for reporting systematic reviews. Bmj. 2021;372:n71. Epub 2021/03/31. doi: 10.1136/bmj.n71. PubMed PMID: 33782057.
21. Hupe M. EndNote X9. Journal of Electronic Resources in Medical Libraries. 2019;16(3-4):117-9.
22. Kellermeyer L, Harnke B, Knight S. Covidence and rayyan. Journal of the Medical Library Association: JMLA. 2018;106(4):580.
23. Birks M, Mills J. Quality in qualitative research. Qualitative methodology: A practical guide. 2014:221-36.
24. Danbjørg DB, Wagner L, Clemensen J. Do families after early postnatal discharge need new ways to communicate with the hospital? A feasibilility study. Midwifery. 2014;30(6):725-32.
25. Danbjørg DB, Wagner L, Clemensen J. Designing, developing, and testing an app for parents being discharged early postnatally. The Journal for Nurse Practitioners. 2014;10(10):794-802.
26. Danbjørg DB, Wagner L, Kristensen BR, Clemensen J. Intervention among new parents followed up by an interview study exploring their experiences of telemedicine after early postnatal discharge. Midwifery. 2015;31(6):574-81.
27. Garne Holm K, Brødsgaard A, Zachariassen G, Smith AC, Clemensen J. Parent perspectives of neonatal tele-homecare: A qualitative study. J Telemed Telecare. 2019;25(4):221-9. Epub 2018/05/25. doi: 10.1177/1357633x18765059. PubMed PMID: 29792079.
28. Garne K, Brødsgaard A, Zachariassen G, Clemensen J. Telemedicine in neonatal home care: identifying parental needs through participatory design. JMIR research protocols. 2016;5(3):e100.
29. Lindberg I, Christensson K, Öhrling K. Parents' experiences of using videoconferencing as a support in early discharge after childbirth. Midwifery. 2009;25(4):357-65.
30. Shorey S, Ng ED. Evaluation of mothers' perceptions of a technology-based supportive educational parenting program (part 2): Qualitative study. Journal of medical Internet research. 2019;21(2):e11065.
31. Shorey S, Yang YY, Dennis C-L. A mobile health app–based postnatal educational program (Home-but not alone): descriptive qualitative study. Journal of medical Internet research. 2018;20(4):e119.
32. Braun V, Clarke V. Reflecting on reflexive thematic analysis. Qualitative research in sport, exercise and health. 2019;11(4):589-97.
33. Wilson D, Sheikh A, Görgens M, Ward K. Technology and Universal Health Coverage: Examining the role of digital health. J Glob Health. 2021;11:16006. Epub 2021/12/17. doi: 10.7189/jogh.11.16006. PubMed PMID: 34912559; PubMed Central PMCID: PMCPMC8645240 (available upon request from the corresponding author), and declare no conflicts of interest.

34. Awad A, Trenfield SJ, Pollard TD, Ong JJ, Elbadawi M, McCoubrey LE, et al. Connected healthcare: Improving patient care using digital health technologies. Advanced Drug Delivery Reviews. 2021;178:113958.
35. Kern-Goldberger AR, Srinivas SK. Telemedicine in obstetrics. Clinics in Perinatology. 2020;47(4):743-57.
36. Alsem MW, Verhoef M, Braakman J, van Meeteren KM, Siebes RC, Jongmans MJ, et al. Parental empowerment in paediatric rehabilitation: exploring the role of a digital tool to help parents prepare for consultation with a physician. Child: Care, Health and Development. 2019;45(5):623-36.
37. Salonen AH, Kaunonen M, Åstedt-Kurki P, Järvenpää AL, Tarkka MT. Development of an internet-based intervention for parents of infants. Journal of Advanced Nursing. 2008;64(1):60-72.
38. Salonen AH, Kaunonen M, Åstedt-Kurki P, Järvenpää A-L, Isoaho H, Tarkka M-T. Effectiveness of an internet-based intervention enhancing Finnish parents' parenting satisfaction and parenting self-efficacy during the postpartum period. Midwifery. 2011;27(6):832-41.
39. Salonen AH, Pridham KF, Brown RL, Kaunonen M. Impact of an internet-based intervention on Finnish mothers' perceptions of parenting satisfaction, infant centrality and depressive symptoms during the postpartum year. Midwifery. 2014;30(1):112-22.
40. Slomian J, Emonts P, Vigneron L, Acconcia A, Reginster J-Y, Oumourgh M, et al. Meeting the needs of mothers during the postpartum period: using co-creation workshops to find technological solutions. JMIR Research Protocols. 2017;6(5):e6831.
41. Sondaal SFV, Browne JL, Amoakoh-Coleman M, Borgstein A, Miltenburg AS, Verwijs M, et al. Assessing the effect of mHealth interventions in improving maternal and neonatal care in low-and middle-income countries: a systematic review. PloS one. 2016;11(5):e0154664.
42. Uscher-Pines L, Ghosh-Dastidar B, Bogen DL, Ray KN, Demirci JR, Mehrotra A, et al. Feasibility and effectiveness of telelactation among rural breastfeeding women. Academic pediatrics. 2020;20(5):652-9.
43. Rasmussen SA, Jamieson DJ. Pregnancy, postpartum care, and COVID-19 vaccination in 2021. Jama. 2021;325(11):1099-100.
44. Gutiérrez MM-C, Durán-Vila A, Ruiz-Labarta J, Payá-Martínez P, Pintado Recarte P, Bujan J, et al. A new multiplatform model for outpatient prenatal and postpartum care in a cohort of covid-19-affected obstetric patients. International Journal of Environmental Research and Public Health. 2021;18(10):5144.
45. Rasmussen SA, Jamieson DJ. Caring for women who are planning a pregnancy, pregnant, or postpartum during the COVID-19 pandemic. Jama. 2020;324(2):190-1.
46. Brown HL, DeNicola N. Telehealth in maternity care. Obstetrics and Gynecology Clinics. 2020;47(3):497-502.
47. Reddy P, Sharma B, Chaudhary K. Digital literacy: A review of literature. International Journal of Technoethics (IJT). 2020;11(2):65-94.
48. Norman CD, Skinner HA. eHealth literacy: essential skills for consumer health in a networked world. Journal of medical Internet research. 2006;8(2):e506.
49. Kuek A, Hakkennes S. Healthcare staff digital literacy levels and their attitudes towards information systems. Health informatics journal. 2020;26(1):592-612.
50. Saplacan D. Situated Abilities: Understanding Everyday Use of ICTs. 2020.

51. Saplacan D, Khaksar W, Torresen J, editors. On Ethical Challenges Raised by Care Robots: A Review of the Existing Regulatory-, Theoretical-, and Research Gaps. 2021 IEEE International Conference on Advanced Robotics and Its Social Impacts (ARSO); 2021: IEEE.
52. Bratteteig T, Saplacan D, Soma R, Svanes Oskarsen J, editors. Strengthening human autonomy in the era of autonomous technology. Contemporary perspectives on interaction with 'autonomous things'. Proceedings of the 11th Nordic Conference on Human-Computer Interaction: Shaping Experiences, Shaping Society; 2020.
53. Lim S, Tan A, Madden S, Hill B. Health professionals' and postpartum women's perspectives on digital health interventions for lifestyle management in the postpartum period: A systematic review of qualitative studies. Frontiers in endocrinology. 2019;10:767.
54. Balakrishnan R, Gopichandran V, Chaturvedi S, Chatterjee R, Mahapatra T, Chaudhuri I. Continuum of Care Services for Maternal and Child Health using mobile technology–a health system strengthening strategy in low and middle income countries. BMC medical informatics and decision making. 2016;16:1-8.
55. Hussain-Shamsy N, Shah A, Vigod SN, Zaheer J, Seto E. Mobile health for perinatal depression and anxiety: scoping review. Journal of medical Internet research. 2020;22(4):e17011.
56. Casiddu N, Cesta A, Cortellessa G, Orlandini A, Porfirione C, Divano A, et al., editors. Robot interface design: The giraff telepresence robot for social interaction. Ambient Assisted Living: Italian Forum 2014; 2015: Springer.
57. Coradeschi S, Cesta A, Cortellessa G, Coraci L, Galindo C, Gonzalez J, et al. GiraffPlus: a system for monitoring activities and physiological parameters and promoting social interaction for elderly. Human-Computer Systems Interaction: Backgrounds and Applications 3. 2014:261-71.
58. Orlandini A, Kristoffersson A, Almquist L, Björkman P, Cesta A, Cortellessa G, et al. Excite project: A review of forty-two months of robotic telepresence technology evolution. Presence: Teleoperators and Virtual Environments. 2016;25(3):204-21.
59. Pages J, Marchionni L, Ferro F, editors. Tiago: the modular robot that adapts to different research needs. International workshop on robot modularity, IROS; 2016.
60. Ake-Kob A, Blazeviciene A, Colonna L, Cartolovni A, Dantas C, Fedosov A, et al. State of the art on ethical, legal, and social issues linked to audio-and video-based AAL solutions. 2021.

Table 1. CASP assessment of included studies*

**Study**	**Q1**	**Q2**	**Q3**	**Q4**	**Q5**	**Q6**	**Q7**	**Q8**	**Q9**	**Q10**	**Sum**
Danbjørg et al. 2014 [24]	1	1	1	1	1	1	1	1	1	1	10
Danbjørg et al. 2014 [25]	1	1	1	1	1	1	1	1	1	1	10
Danbjørg et al. 2015	1	1	1	1	1	1	1	1	1	1	10
Garne Holm et al. 2019											10

Garne et al. 2016	1	1	1	1	1	1	1	1	1	1	10
Lindberg et al. 2009	1	1	1	1	1	1	1	1	1	1	10
Shorey et al. 2018	1	1	1	1	1	1	1	1	1	1	10
Shorey et al. 2019	1	1	1	1	1	1	1	1	1	1	10

*CASP Checklist (Yes=1; Can't tell=X; No=0)

10 questions to help you make sense of qualitative research: Q1 Was there a clear statement of the aims of the research? Q2 Is a qualitative methodology appropriate? Q3 Was the research design appropriate to address the aims of the research? Q4 Was the recruitment strategy appropriate to the aims of the research? Q5 Was the data collected in a way that addressed the research issue? Q6 Has the relationship between researcher and participants been adequately considered? Q7 Have ethical issues been taken into consideration? Q8 Was the data analysis sufficiently rigorous? Q9 Is there a clear statement of findings? Q10 How valuable is the research?

Table 2. Overview of included studies

**Author, year, country**	**Aim**	**Welfare technology used**	**Method design**	**Participants**	**Data collection**	**Data analysis**	**Results**
Danbjørg et al. 2015 Denmark	To explore how parents experienced the use of telemedicine following early postnatal discharge	Application	Participatory and qualitative design	27 ~~mothers~~parents & 11 ~~fathers~~partners	Interviews	Systematic text condensation	Parents were confident in using the application (app) and found it natural to seek information and communicate online; they did not experience any barriers in contacting the nurses using asynchronous communication. The functionalities of the app (i.e., chat, knowledge base and automated messages) met the needs of the new parents and induced a sense of security and parental self-efficacy.
Danbjørg et al. 2014 Denmark	To describe the process of the design, development, and testing of an application as a viable information technology solution	Application	Participatory design with in-depth consultation with users	9 ~~mothers~~parents & 1 father	Participant observation, field notes, individual telephone interviews	Systematic text condensation	The families found it natural to communicate online, and they did not feel any barriers. The app met the new families' needs for follow-up support. The testing suggests that the new families and the nurses found the app viable, but the app requires refinements and wider testing.
Danbjørg et al. 2014 Denmark	To identify the nursing support needs of new parents and their infants during the first seven days postpartum, by drawing on the experiences of all stakeholders in early postnatal discharge from hospital, and thereby gaining new knowledge to investigate further whether telemedicine is a viable option in providing the required support	Telemedicine	Participatory and qualitative design	19 parents	Participant observation, interviews, workshop	Systematic text condensation	Families that were discharged early requested more availability from the healthcare system to respond to their concerns and questions during the postnatal period. They asked for new ways to communicate that would meet their needs for more individualised and timely information and guidance. Study results indicate that it may be possible to meet the follow-up support needs of these families through new ways to communicate, such as online communication.
Garne et al. 2016 Denmark	To identify parents' needs when seeking to provide neonatal home care supported by telemedicine	Telemedicine	Participatory and qualitative design	19 parents	Observational studies, individual interviews, and focus group interviews	Systematic text condensation	This study supports the use of telemedicine for neonatal home care because it gives parents the feeling of being a family and promotes their self-efficacy. Telemedicine-provided home care gives parents the guidance they need from nurses when they do not require hands-on support. Parents also outlined the need for a technological 'bell cord', such as videoconferencing, timely e-mail communication, and a knowledge base of information regarding infant nutrition and breastfeeding.
Garne Holm et al. 2019 Denmark	To explore parents' experiences with neonatal tele-homecare (NTH)	Application	Qualitative design	49 parents	Interviews	Systematic text condensation	The results from the study identified that NTH could facilitate family-centred care (FCC). The telehealth service served as a personal lifeline to clinical expertise, which was easily accessed. Through the videoconference sessions, the parents felt acknowledged regarding their parenting skills and their observations of their infants. Study findings indicate that tele-homecare can be a supportive practice for parents.
Linberg et al. 2009 Sweden	To describe parents' experiences of using videoconferencing when discharged early from a maternity unit	Videoconferencing	Combination of quantitative and qualitative design	9 couples	Questionnaires and interviews	Thematic content analysis	Study results showed that parents were confident regarding using the technology, being face-to-face during the videoconference (VC), having control over their privacy, and feeling confident especially 'when worries and concerns were met, and answers were received'. Using VC as a support in cases of early discharge after childbirth can facilitate a meeting that enables new parents to be guided by the midwife in their transition to parenthood.
Shorey et al. 2018 Singapore	To explore the views of parents of newborns with regard to the content and delivery of a mobile health (mHealth) app-based postnatal educational programme	Application	Qualitative design	17 participants (5 couples, 4 ~~fathers~~partners, and 3 ~~mothers~~parents)	Interviews	Thematic content analysis	Study participants reported that mobile health app-based support was a good source of information that was tailored to the local context. The parents assessed the facilitator of the featured communication platform, a midwife, as providing trustworthy advice. Belonging to a virtual community beyond the hospital gave the parents the feeling that they were not alone and were supported by healthcare professionals.
Shorey et al. 2019 Singapore	To examine the experiences and perceptions of participants in a supportive education parenting programme intervention study	Application	Qualitative design	16 ~~mothers~~parents (6 control and 10 intervention)	Interviews	Thematic content analysis	~~Mothers~~Parents from the intervention group reported having good experiences with receiving sufficient mobile health support. The multifeatured, technology-based intervention was effective in improving parental outcomes and was well received by parents.

Figure 1 Example search in PubMed

01.03.21

Search 1: (((((((((((((((("new mother")) OR ("new mothersparents")) OR (birth)) OR (Childbirth)) OR (primiparas)) OR (multiparas)) OR (parent)) OR ("new parent")) OR ("new parents")) OR ("home based postnatal care")) OR (postpartum)) OR ("postnatal care")) OR ("managed care")) OR ("Ambulatory care")) OR ("After care")) OR ("home visit")) OR ("home visits")

Number: 1 032 717

Search 2: ((((((((("health system") OR (telemedicine)) OR ("Health services")) OR ("mobile health")) OR ("Health, mobile")) OR (mhealth)) OR (telehealth)) OR (ehealth)) OR (telecommunication)) OR (telenursing)

Number: 687 119

Search 3: ((((("early postnatal discharge") OR ("early discharge")) OR ("length of stay")) OR ("very early discharge")) OR ("early postpartum discharge")) OR ("early patient discharge") **Number: 127 601**

Search 4: ((((("semistructured interview") OR ("semistructured interviews")) OR ("cross sectional")) OR ("content analysis")) OR (qualitative)) OR ("grounded theory")

Number: 833 989

Search 5: #1 AND #2 AND #3

(((((((((((((((((("new mother")) OR ("new mothersparents")) OR (birth)) OR (Childbirth)) OR (primiparas)) OR (multiparas)) OR (parent)) OR ("new parent")) OR ("new parents")) OR ("home based postnatal care")) OR (postpartum)) OR ("postnatal care")) OR ("managed care")) OR ("Ambulatory care")) OR ("After care")) OR ("home visit")) OR ("home visits")) AND (((((((((("health system") OR (telemedicine)) OR ("Health services")) OR ("mobile health")) OR ("Health, mobile")) OR (mhealth)) OR (telehealth)) OR (ehealth)) OR (telecommunication)) OR (telenursing))) AND (((((("early postnatal discharge") OR ("early discharge")) OR ("length of stay")) OR ("very early discharge")) OR ("early postpartum discharge")) OR ("early patient discharge"))) AND (((((("semistructured interview") OR ("semistructured interviews")) OR ("cross sectional")) OR ("content analysis")) OR (qualitative)) OR ("grounded theory"))

Number: 98

~~Search 6: #5 AND English AND from 2015/1/1 - 2021/12/31~~

~~(((((((((((((((((((("new mother")) OR ("new mothers~~parents~~")) OR (birth)) OR (Childbirth)) OR (primiparas)) OR (multiparas)) OR (parent)) OR ("new parent")) OR ("new parents")) OR ("home based postnatal care")) OR (postpartum)) OR ("postnatal care")) OR ("managed care")) OR ("Ambulatory care")) OR ("After care")) OR ("home visit")) OR ("home visits")) AND (((((((((("health system") OR (telemedicine)) OR ("Health services")) OR ("mobile health")) OR ("Health, mobile")) OR (mhealth)) OR (telehealth)) OR (ehealth)) OR (telecommunication)) OR (telenursing))) AND (((((("early postnatal discharge") OR ("early discharge")) OR ("length of stay")) OR ("very early discharge")) OR ("early postpartum discharge")) OR ("early patient discharge"))) AND (((((("semistructured interview") OR ("semistructured interviews")) OR ("cross sectional")) OR ("content analysis")) OR (qualitative)) OR ("grounded theory")) Filters: English, from 2015/1/1 - 2021/12/31~~

PRISMA flowchart

~~PRISMA flowchart of study selection procedure~~

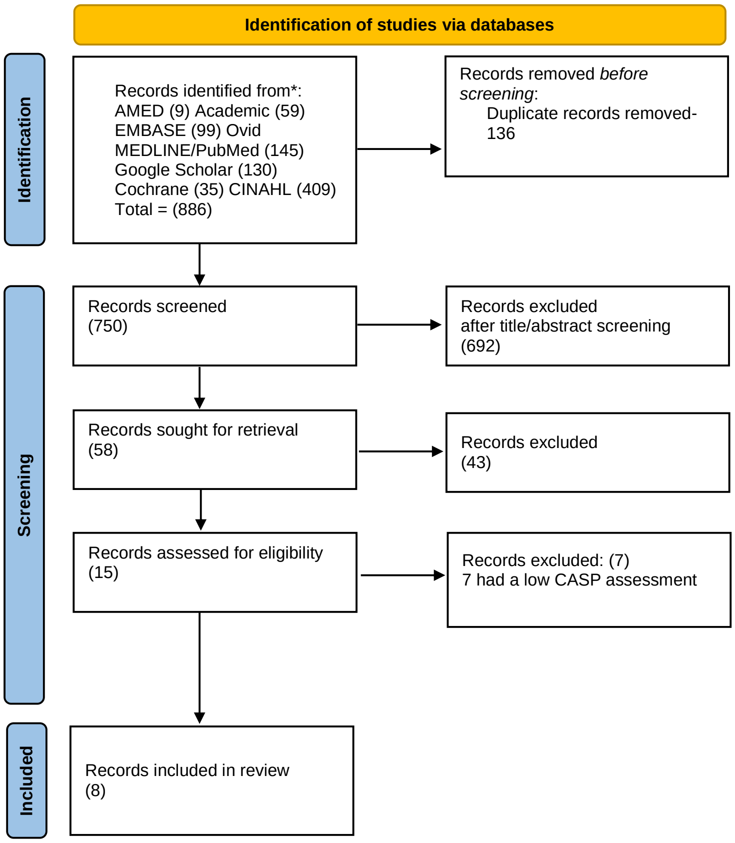

Table 3 Overview of themes and codes

**Theme**	**Flexible digital nursing support**	**Digital literacy**	**Safe parenting with digital support**	**Adequate substitute for physical meetings**
**Codes**	available asynchronous information<br>online chat is preferred<br>good alternative to refreshing old knowledge<br>reassuring to have access to the information one needs<br>digital/app literacy essential for successful use<br>availability of information<br>opportunity to get information repeated<br>opportunity to portion out information based on current needs<br>interactive communication and tutoring<br>new method of communicating with parents<br>internet infrastructure essential for adequate remote nursing<br>written information is preferred<br>greater opportunity for individualized information<br>feeling of closeness<br>to get full attention<br>to get the time they need<br>confirmatory<br>Timesaving<br>support weighs more than fear of private disturbances	Being surprised with early discharge<br>equal need for support regardless of primipara or multipara<br>technology integrated into everyday life<br>accustomed to using technology<br>digital supervision increases the sense of manageability<br>asymmetry in digital literacy between healthcare professionals and parents<br>Healthcare professionals do not have as good digital skills as their parents<br>liberating<br>interactive collaboration with the profession<br>becomes more confident in their own capacity and competence<br>have skills to use the app<br>teach health professionals digital skills<br>to be engaged<br>empowered parents<br>mutual knowledge exchange<br>feeling of being in control<br>feeling of being prepared and strong in their new role<br>uncertain in his new role<br>need for frequent support<br>do not want to be discharged early<br>do not feel welcome<br>Own networks as alternative support Seek information on your own<br>feeling of insufficient support	covers the need for emotional support<br>feeling of safety<br>it turned out better than feared<br>nice to know that you can handle yourself<br>positive to be able to communicate directly with the midwife<br>to be relaxed and natural in front of the camera<br>follows own routines and rules at home<br>feeling of safety<br>relaxed atmosphere<br>relaxed home environment<br>important with regular access to information<br>sense of control<br>easier to absorb information in a quiet home environment<br>digital lifeline<br>emotional support<br>direct and personalized communication<br>home environment	Shared experiences regarding restriction in the private area<br>good substitute for physical meeting<br>longer use of the technology desirable<br>camera makes it possible to compose oral communication<br>very good alternative to hospital stays<br>technology is the lifeline to professional support around the clock<br>brilliant lifeline with professional support<br>efficiently with automated responses<br>practical solutions that prevent unnecessary hospital visits<br>photos and videos replace hospital visits<br>more and more easily accessible health personnel via the app<br>feel free to contact if needed<br>dare to ask more questions via the app<br>modern technology: can use as oral communication, with videos, with pictures, with written questions<br>24 hours useful<br>good alternative to stay in hospital

Formatted: Left: 0.32", Right: 0.32", Top: 0.29", Bottom: 0.29", Width: 11", Height: 8.5"

	new communication method<br><br>information can be made available via SMS or chat<br><br>a page with frequently asked questions<br><br>useful and easy App<br><br>positive feedback important<br><br>app as advice source most credible<br><br>round-the-clock support<br><br>available support if needed<br><br>easy to absorb information<br><br>available information<br><br>to get positive confirmation<br><br>availability of necessary personal information, up-to-date, asynchronous, reminder many times<br><br>feedback and attention<br><br>available support and timesaving new communication method	questioning of advice given<br><br>comparing information<br><br>need for personalized information<br><br>knowledge asymmetry in ICT between new parents and care staff<br><br>Integration of App in daily life<br><br>Feeling of control and support<br><br>Flexibility<br><br>Less stressful /calm<br><br>Feel insecure<br><br>strengthens self-confidence<br><br>experience of self-confidence<br><br>can better understand the baby's behaviour<br><br>dare easier asked<br><br>share their experiences with other parents<br><br>more confident<br><br>help to find out your own problem-solving strategies<br><br>support with interactive collaboration<br><br>share experiences<br><br>empower self confidence<br><br>bonding and strengthening family members		

Figure 3 Overview themes a sub-theme

**Theme**	**Flexible digital support**	**Digital literacy**	**Safe parenting with digital**	**Adequate substitute for physical meetings**
**Sub-theme**	Availability of necessary personal information<br><br>Up-to-date, asynchronous reminders<br><br>Feedback and attention<br><br>Available support<br><br>Timesaving by the new communication method	Support with interactive collaboration (app etc)<br><br>Sharing experiences<br><br>Empowering self-confidence<br><br>Bonding and strengthening family members	Emotional support<br><br>Direct and personalised communication<br><br>Home environment	Modern communication technology<br><br>Communication complemented with videos, pictures, and text<br><br>24-hour accessible alternative support

List of search strategies for all databases

Søkehistorikk pubmed

01.03.21

Søk 1: ((((((((((((((("new mother")) OR ("new parents")) OR (birth)) OR (Childbirth)) OR (primiparas)) OR (multiparas)) OR (parent)) OR ("new parent")) OR ("new parents")) OR ("home based postnatal care")) OR (postpartum)) OR ("postnatal care")) OR ("managed care")) OR ("Ambulatory care")) OR ("After care")) OR ("home visit")) OR ("home visits")

Antall: 1 032 717

Søk 2: ((((((((("health system") OR (telemedicine)) OR ("Health services")) OR ("mobile health")) OR ("Health, mobile")) OR (mhealth)) OR (telehealth)) OR (ehealth)) OR (telecommunication)) OR (telenursing)

Antall: 687 119

Søk 3: ((((("early postnatal discharge") OR ("early discharge")) OR ("length of stay")) OR ("very early discharge")) OR ("early postpartum discharge")) OR ("early patient discharge")

Antall: 127 601

Søk 4: ((((("semistructured interview") OR ("semistructured interviews")) OR ("cross sectional")) OR ("content analysis")) OR (qualitative)) OR ("grounded theory")

Antall: 833 989

Søk 5: #1 AND #2 AND #3

(((((((((((((((((("new mother")) OR ("new parents")) OR (birth)) OR (Childbirth)) OR (primiparas)) OR (multiparas)) OR (parent)) OR ("new parent")) OR ("new parents")) OR ("home based postnatal care")) OR (postpartum)) OR ("postnatal care")) OR ("managed care")) OR ("Ambulatory care")) OR ("After care")) OR ("home visit")) OR ("home visits")) AND (((((((((("health system") OR (telemedicine)) OR ("Health services")) OR ("mobile health")) OR ("Health, mobile")) OR (mhealth)) OR (telehealth)) OR (ehealth)) OR (telecommunication)) OR (telenursing))) AND (((((("early postnatal discharge") OR ("early discharge")) OR ("length of stay")) OR ("very early discharge")) OR ("early postpartum discharge")) OR ("early patient discharge"))) AND (((((("semistructured interview") OR ("semistructured interviews")) OR ("cross sectional")) OR ("content analysis")) OR (qualitative)) OR ("grounded theory"))

Antall: 98

Søk 6: #5 AND English AND from 2015/1/1 - 2021/12/31

((((((((((((((((((("new mother")) OR ("new parents")) OR (birth)) OR (Childbirth)) OR (primiparas)) OR (multiparas)) OR (parent)) OR ("new parent")) OR ("new parents")) OR ("home based postnatal care")) OR (postpartum)) OR ("postnatal care")) OR ("managed care")) OR ("Ambulatory care")) OR ("After care")) OR ("home visit")) OR ("home visits")) AND (((((((("health system") OR (telemedicine)) OR ("Health services")) OR ("mobile health")) OR ("Health, mobile")) OR (mhealth)) OR (telehealth)) OR (ehealth)) OR (telecommunication)) OR (telenursing))) AND (((((("early postnatal discharge") OR ("early discharge")) OR ("length of stay")) OR ("very early discharge")) OR ("early postpartum discharge")) OR ("early patient discharge"))) AND (((((("semistructured interview") OR ("semistructured interviews")) OR ("cross sectional")) OR ("content analysis")) OR (qualitative)) OR ("grounded theory")) Filters: English, from 2015/1/1 - 2021/12/31

Antall 47

Konsepttabell:

Nybakte foreldre/barselperiode	Telemedisin	Tidlig utskriving	Design + andre avgrensninger
«New mother»<br>«New parents»	«health system*»	«Early postnatal discharge»	«Semistructured interview»<br>«Semistructured interviews»
~~Patient*~~	**Telemedicine**	«Early discharge»	**Cross-sectional**
Birth<br>**Childbirth**	**health services**	«**Length of stay**»<br>Forslag: «~~Reduce length of stay~~»	Qualitative
**Primiparas**	«mobile health»	«very early discharge»	«content analysis»
	Health, mobile		Grounded theory
**Multiparas**	Mhealth	«Early postpartum discharge»	~~Nordic countries~~

**Postpartum**	**Telehealth**	«Early patient discharge»	5 siste årene
**Parent**	ehealth		
«New parent»<br>«New parents»	**Telecommunication**		
«home-based postnatal care»	**Telenursing**		
**«postnatal care»**			
**«managed care»**			
**«ambulatory care»**			
**«After care»**			
«home visit»<br>«home visits»			

AMED 04.03.21

Nybakte foreldre/barselperiode	Telemedisin	Tidlig utskriving	Design + andre avgrensninger
New mother*	~~health system*~~	~~«Early postnatal discharge»~~<br>~~0 treff~~	«Semistructured interview*»
~~Patient*~~	**Telemedicine**	~~«Early discharge»~~	Cross-sectional
Birth<br>Childbirth	~~«health service»~~	~~**«Length of stay»**~~	Qualitative

Formatted: English (United Kingdom)

		Forslag: «Reduce length of stay»	
Primiparas	«mobile health»	«very early discharge»	«content analysis»
	«Health, mobile»		«Grounded theory»
Multiparas	Mhealth	«Early postpartum discharge»<br><br>0 treff	Nordic countries
Postpartum	Telehealth	«Early patient discharge»	5 siste årene
Parent	ehealth		
New parent*	Telecommunication		
home-based postnatal care (0 treff)	Telenursing		
postnatal care			
managed care			
**ambulatory care**			
**After care**			
«home visit*»			

Må fjerne generelle søkeord. Får mye treff som omhandler andre temaer. Se de som er strøket over.

Formatted: English (United Kingdom)

Antall treff: 9

SØK 1 EMBASE 04.03.21
Search for: limit 32 to (english language and yr="2015 -Current")
Results: 11

Database: Embase <1980 to 2021 Week 08>
Search Strategy:
----
1 new mother*.mp. (2121)
2 birth.mp. or exp birth/ (454814)
3 exp childbirth/ or childbirth.mp. (69271)
4 primiparas.mp. or exp primipara/ (5300)
5 multiparas.mp. or exp multipara/ (8261)
6 postpartum.mp. (78472)
7 parent*.mp. or exp parent/ (722613)
8 new parent*.mp. (1269)
9 home-based postnatal care.mp. (13)
10 postnatal care.mp. or exp postnatal care/ (115501)
11 1 or 2 or 3 or 4 or 5 or 6 or 7 or 8 or 9 or 10 (1247455)
12 telemedicine.mp. or exp telemedicine/ (51648)
13 mobile health.mp. (6123)
14 health, mobile.mp. (137)
15 mhealth.mp. (5257)
16 telehealth.mp. or exp telehealth/ (55832)
17 ehealth.mp. or exp telehealth/ (56550)
18 telecommunication.mp. or exp telecommunication/ (81421)
19 telenursing.mp. or exp telenursing/ (427)
20 12 or 13 or 14 or 15 or 16 or 17 or 18 or 19 (92227)
21 exp semi structured interview/ or semistructured interview*.mp. (49045)
22 cross sectional.mp. or exp cross-sectional study/ (604192)
23 qualitative.mp. or qualitative research/ (344533)
24 content analysis.mp. or exp content analysis/ (38633)
25 grounded theory.mp. or exp grounded theory/ (15927)
26 21 or 22 or 23 or 24 or 25 (979109)
27 very early discharge.mp. (41)
28 exp hospital discharge/ (134931)
29 postnatal discharge.mp. (74)
30 length of stay.mp. or exp "length of stay"/ (217905)
31 27 or 28 or 29 or 30 (333334)

Formatted: English (United Kingdom)

32 11 and 20 and 26 and 31 (20)
33 limit 32 to (english language and yr="2015 -Current") (11)
34 new mother*.mp. (2121)
35 birth.mp. or exp birth/ (454814)
36 exp childbirth/ or childbirth.mp. (69271)
37 primiparas.mp. or exp primipara/ (5300)
38 multiparas.mp. or exp multipara/ (8261)
39 postpartum.mp. (78472)
40 parent*.mp. or exp parent/ (722613)
41 new parent*.mp. (1269)
42 home-based postnatal care.mp. (13)
43 postnatal care.mp. or exp postnatal care/ (115501)
44 34 or 35 or 36 or 37 or 38 or 39 or 40 or 41 or 42 or 43 (1247455)
45 telemedicine.mp. or exp telemedicine/ (51648)
46 mobile health.mp. (6123)
47 health, mobile.mp. (137)
48 mhealth.mp. (5257)
49 telehealth.mp. or exp telehealth/ (55832)
50 ehealth.mp. or exp telehealth/ (56550)
51 telecommunication.mp. or exp telecommunication/ (81421)
52 telenursing.mp. or exp telenursing/ (427)
53 45 or 46 or 47 or 48 or 49 or 50 or 51 or 52 (92227)
54 exp semi structured interview/ or semistructured interview*.mp. (49045)
55 cross sectional.mp. or exp cross-sectional study/ (604192)
56 qualitative.mp. or qualitative research/ (344533)
57 content analysis.mp. or exp content analysis/ (38633)
58 grounded theory.mp. or exp grounded theory/ (15927)
59 54 or 55 or 56 or 57 or 58 (979109)
60 very early discharge.mp. (41)
61 exp hospital discharge/ (134931)
62 postnatal discharge.mp. (74)
63 length of stay.mp. or exp "length of stay"/ (217905)
64 60 or 61 or 62 or 63 (333334)
65 44 and 53 and 59 and 64 (20)
66 limit 65 to (english language and yr="2015 -Current") (11)

****

SØK 2 i Embase 04.03.21
Search for: limit 24 to (english language and yr="2015 -Current")

Results: 88

Database: Embase <1980 to 2021 Week 08>

Search Strategy:

----

1 new mother*.mp. (2121)
2 primiparas.mp. or exp primipara/ (5300)
3 multiparas.mp. or exp multipara/ (8261)
4 postpartum.mp. (78472)
5 new parent*.mp. (1269)
6 home based postnatal care.mp. (13)
7 postnatal care.mp. or exp postnatal care/ (115501)
8 1 or 2 or 3 or 4 or 5 or 6 or 7 (178349)
9 telemedicine.mp. or exp telemedicine/ (51648)
10 "mobile health".mp. (6123)
11 health, mobile.mp. (137)
12 mhealth.mp. (5257)
13 telehealth.mp. or exp telehealth/ (55832)
14 ehealth.mp. (4778)
15 telecommunication.mp. or exp telecommunication/ (81421)
16 telenursing.mp. or exp telenursing/ (427)
17 9 or 10 or 11 or 12 or 13 or 14 or 15 or 16 (92227)
18 exp semi structured interview/ or semistructured interview*.mp. (49045)
19 cross sectional.mp. or exp cross-sectional study/ (604192)
20 qualitative.mp. or exp qualitative research/ (344815)
21 content analysis.mp. or exp content analysis/ (38633)
22 grounded theory.mp. or exp grounded theory/ (15927)
23 18 or 19 or 20 or 21 or 22 (979373)
24 8 and 17 and 23 (112)
25 limit 24 to (english language and yr="2015 -Current") (88)

Formatted: English (United Kingdom)

Friday, February 26, 2021 1:52:18 AM

#	Query	Limiters/Expanders	Last Run Via	Results
S43	S15 AND S32 AND S38 AND S41	Limiters - Published Date: 20150101-20211231; English Language; Peer Reviewed<br>Expanders - Apply equivalent subjects<br>Search modes - Boolean/Phrase	Interface - EBSCOhost Research Databases<br>Search Screen - Advanced Search<br>Database - CINAHL with Full Text	35
S42	S15 AND S32 AND S38 AND S41	Expanders - Apply equivalent subjects<br>Search modes - Boolean/Phrase	Interface - EBSCOhost Research Databases<br>Search Screen - Advanced Search<br>Database - CINAHL with Full Text	38
S41	S24 OR S39 OR S40	Expanders - Apply equivalent subjects<br>Search modes - Boolean/Phrase	Interface - EBSCOhost Research Databases<br>Search Screen - Advanced Search<br>Database - CINAHL with Full Text	123,018
S40	"health service*"	Limiters - Published Date: 20150101-20211231; English Language; Peer Reviewed<br>Expanders - Apply equivalent subjects<br>Search modes - Boolean/Phrase	Interface - EBSCOhost Research Databases<br>Search Screen - Advanced Search<br>Database - CINAHL with Full Text	79,909

Formatted: English (United Kingdom)

S39	"health system*"	Limiters - Published Date: 20150101-20211231; English Language; Peer Reviewed<br>Expanders - Apply equivalent subjects<br>Search modes - Boolean/Phrase	Interface - EBSCOhost Research Databases<br>Search Screen - Advanced Search<br>Database - CINAHL with Full Text	16,240

S38	S33 OR S34 OR S35 OR S36 OR S37	Expanders - Apply equivalent subjects<br>Search modes - Boolean/Phrase	Interface - EBSCOhost Research Databases<br>Search Screen - Advanced Search<br>Database - CINAHL with Full Text	440,777
S37	"grounded theor*"	Expanders - Apply equivalent subjects<br>Search modes - Boolean/Phrase	Interface - EBSCOhost Research Databases<br>Search Screen - Advanced Search<br>Database - CINAHL with Full Text	19,434
S36	"content analys*"	Expanders - Apply equivalent subjects<br>Search modes - Boolean/Phrase	Interface - EBSCOhost Research Databases<br>Search Screen - Advanced Search<br>Database - CINAHL with Full Text	45,885
S35	qualitative	Expanders - Apply equivalent subjects<br>Search modes - Boolean/Phrase	Interface - EBSCOhost Research Databases<br>Search Screen - Advanced Search<br>Database - CINAHL with Full Text	178,984
S34	"cross sectional"	Expanders - Apply equivalent subjects<br>Search modes - Boolean/Phrase	Interface - EBSCOhost Research Databases<br>Search Screen - Advanced Search<br>Database - CINAHL with Full Text	241,548

S33	semistructured interview*"	Expanders - Apply equivalent subjects<br>Search modes - Boolean/Phrase	Interface - EBSCOhost Research Databases<br>Search Screen - Advanced Search<br>Database - CINAHL with Full Text	7,764
S32	S25 OR S26 OR S27 OR S28 OR S29 OR S30 OR S31	Expanders - Apply equivalent subjects<br>Search modes - Boolean/Phrase	Interface - EBSCOhost Research Databases<br>Search Screen - Advanced Search<br>Database - CINAHL with Full Text	57,677

S31	"Early patient discharge"	Expanders - Apply equivalent subjects<br>Search modes - Boolean/Phrase	Interface - EBSCOhost Research Databases<br>Search Screen - Advanced Search<br>Database - CINAHL with Full Text	1,012
S30	"Early postpartum discharge"	Expanders - Apply equivalent subjects<br>Search modes - Boolean/Phrase	Interface - EBSCOhost Research Databases<br>Search Screen - Advanced Search<br>Database - CINAHL with Full Text	42
S29	"very early discharge"	Expanders - Apply equivalent subjects<br>Search modes - Boolean/Phrase	Interface - EBSCOhost Research Databases<br>Search Screen - Advanced Search<br>Database - CINAHL with Full Text	8
S28	"Reduce length of stay"	Expanders - Apply equivalent subjects<br>Search modes - Boolean/Phrase	Interface - EBSCOhost Research Databases<br>Search Screen - Advanced Search<br>Database - CINAHL with Full Text	217
S27	"length of stay"	Expanders - Apply equivalent subjects<br>Search modes - Boolean/Phrase	Interface - EBSCOhost Research Databases<br>Search Screen - Advanced Search<br>Database - CINAHL with Full Text	56,269

S26	"early discharge"	Expanders - Apply equivalent subjects<br>Search modes - Boolean/Phrase	Interface - EBSCOhost Research Databases<br>Search Screen - Advanced Search<br>Database - CINAHL with Full Text	1,693
S25	"early postnatal discharge"	Expanders - Apply equivalent subjects<br>Search modes - Boolean/Phrase	Interface - EBSCOhost Research Databases<br>Search Screen - Advanced Search<br>Database - CINAHL with Full Text	27

S24	S16 OR S17 OR S18 OR S19 OR S20 OR S21 OR S22 OR S23	Expanders - Apply equivalent subjects<br>Search modes - Boolean/Phrase	Interface - EBSCOhost Research Databases<br>Search Screen - Advanced Search<br>Database - CINAHL with Full Text	34,390
S23	Telenursing	Expanders - Apply equivalent subjects<br>Search modes - Boolean/Phrase	Interface - EBSCOhost Research Databases<br>Search Screen - Advanced Search<br>Database - CINAHL with Full Text	2,188
S22	telecommun*	Expanders - Apply equivalent subjects<br>Search modes - Boolean/Phrase	Interface - EBSCOhost Research Databases<br>Search Screen - Advanced Search<br>Database - CINAHL with Full Text	3,295
S21	ehealth	Expanders - Apply equivalent subjects<br>Search modes - Boolean/Phrase	Interface - EBSCOhost Research Databases<br>Search Screen - Advanced Search<br>Database - CINAHL with Full Text	17,212
S20	telehealth	Expanders - Apply equivalent subjects<br>Search modes - Boolean/Phrase	Interface - EBSCOhost Research Databases<br>Search Screen - Advanced Search<br>Database - CINAHL with Full Text	20,686

S19	mhealth	Expanders - Apply equivalent subjects<br>Search modes - Boolean/Phrase	Interface - EBSCOhost Research Databases<br>Search Screen - Advanced Search<br>Database - CINAHL with Full Text	16,640
S18	"Health, mobile"	Expanders - Apply equivalent subjects<br>Search modes - Boolean/Phrase	Interface - EBSCOhost Research Databases<br>Search Screen - Advanced Search<br>Database - CINAHL with Full Text	67

S17	"mobile health"	Expanders - Apply equivalent subjects<br>Search modes - Boolean/Phrase	Interface - EBSCOhost Research Databases<br>Search Screen - Advanced Search<br>Database - CINAHL with Full Text	18,886
S16	Telemedicine	Expanders - Apply equivalent subjects<br>Search modes - Boolean/Phrase	Interface - EBSCOhost Research Databases<br>Search Screen - Advanced Search<br>Database - CINAHL with Full Text	20,685
S15	S1 OR S2 OR S3 OR S4 OR S5 OR S6 OR S7 OR S8 OR S9 OR S10 OR S11 OR S12 OR S13 OR S14	Expanders - Apply equivalent subjects<br>Search modes - Boolean/Phrase	Interface - EBSCOhost Research Databases<br>Search Screen - Advanced Search<br>Database - CINAHL with Full Text	390,678
S14	"home visit*"	Expanders - Apply equivalent subjects<br>Search modes - Boolean/Phrase	Interface - EBSCOhost Research Databases<br>Search Screen - Advanced Search<br>Database - CINAHL with Full Text	9,884
S13	"after care"	Expanders - Apply equivalent subjects<br>Search modes - Boolean/Phrase	Interface - EBSCOhost Research Databases<br>Search Screen - Advanced Search<br>Database - CINAHL with Full Text	15,681

S12	"ambulatory care"	Expanders - Apply equivalent subjects<br>Search modes - Boolean/Phrase	Interface - EBSCOhost Research Databases<br>Search Screen - Advanced Search<br>Database - CINAHL with Full Text	23,994
S11	"managed care"	Expanders - Apply equivalent subjects<br>Search modes - Boolean/Phrase	Interface - EBSCOhost Research Databases<br>Search Screen - Advanced Search<br>Database - CINAHL with Full Text	16,183

S10	"postnatal care"	Expanders - Apply equivalent subjects<br>Search modes - Boolean/Phrase	Interface - EBSCOhost Research Databases<br>Search Screen - Advanced Search<br>Database - CINAHL with Full Text	6,251
S9	"home based postnatal care"	Expanders - Apply equivalent subjects<br>Search modes - Boolean/Phrase	Interface - EBSCOhost Research Databases<br>Search Screen - Advanced Search<br>Database - CINAHL with Full Text	11
S8	"new parent*"	Expanders - Apply equivalent subjects<br>Search modes - Boolean/Phrase	Interface - EBSCOhost Research Databases<br>Search Screen - Advanced Search<br>Database - CINAHL with Full Text	694
S7	parent*	Expanders - Apply equivalent subjects<br>Search modes - Boolean/Phrase	Interface - EBSCOhost Research Databases<br>Search Screen - Advanced Search<br>Database - CINAHL with Full Text	186,263
S6	postpartum	Expanders - Apply equivalent subjects<br>Search modes - Boolean/Phrase	Interface - EBSCOhost Research Databases<br>Search Screen - Advanced Search<br>Database - CINAHL with Full Text	28,956

S5	multipar*	Expanders - Apply equivalent subjects<br>Search modes - Boolean/Phrase	Interface - EBSCOhost Research Databases<br>Search Screen - Advanced Search<br>Database - CINAHL with Full Text	4,846
S4	primipara*	Expanders - Apply equivalent subjects<br>Search modes - Boolean/Phrase	Interface - EBSCOhost Research Databases<br>Search Screen - Advanced Search<br>Database - CINAHL with Full Text	2,097

S3	Childbirth	Expanders - Apply equivalent subjects Search modes - Boolean/Phrase	Interface - EBSCOhost Research Databases Search Screen - Advanced Search Database - CINAHL with Full	36,233
S2	Birth	Expanders - Apply equivalent subjects Search modes - Boolean/Phrase	Interface - EBSCOhost Research Databases Search Screen - Advanced Search Database - CINAHL with Full	117,861
S1	"new mother*"	Expanders - Apply equivalent subjects Search modes - Boolean/Phrase	Interface - EBSCOhost Research Databases Search Screen - Advanced Search Database - CINAHL with Full	1,705

Formatted: English (United Kingdom)

Formatted: English (United Kingdom)

Formatted: English (United Kingdom)

Formatted: English (United Kingdom)

Formatted: English (United Kingdom)

Formatted: English (United Kingdom)

Wednesday, March 03, 2021 8:02:15 AM

#	Query	Limiters/Expanders	Last Run Via	Results
S42	S15 AND S26 AND S34 AND S40	Limiters - Published Date: 20150101-20211231; Scholarly (Peer Reviewed) Journals; Language: English<br>Expanders - Apply equivalent subjects<br>Search modes - Boolean/Phrase	Interface - EBSCOhost Research Databases<br>Search Screen - Advanced Search<br>Database - Academic Search Elite	59
S41	S15 AND S26 AND S34 AND S40	Expanders - Apply equivalent subjects<br>Search modes - Boolean/Phrase	Interface - EBSCOhost Research Databases<br>Search Screen - Advanced Search<br>Database - Academic Search Elite	91
S40	S35 OR S36 OR S37 OR S38 OR S39	Expanders - Apply equivalent subjects<br>Search modes - Boolean/Phrase	Interface - EBSCOhost Research Databases<br>Search Screen - Advanced Search<br>Database - Academic Search Elite	606,999
S39	"grounded theory"	Expanders - Apply equivalent subjects<br>Search modes - Boolean/Phrase	Interface - EBSCOhost Research Databases<br>Search Screen - Advanced Search<br>Database - Academic Search Elite	15,273

S38	"content analysis"	Expanders - Apply equivalent subjects<br>Search modes - Boolean/Phrase	Interface - EBSCOhost Research Databases<br>Search Screen - Advanced Search<br>Database - Academic Search Elite	46,633
S37	qualitative	Expanders - Apply equivalent subjects	Interface - EBSCOhost Research Databases<br>Search Screen - Advanced	307,491

		Search modes - Boolean/Phrase	Search Database - Academic Search Elite	
S36	"cross sectional"	Expanders - Apply equivalent subjects Search modes - Boolean/Phrase	Interface - EBSCOhost Research Databases Search Screen - Advanced Search Database - Academic Search Elite	267,280
S35	"semistructured interview*"	Expanders - Apply equivalent subjects Search modes - Boolean/Phrase	Interface - EBSCOhost Research Databases Search Screen - Advanced Search Database - Academic Search Elite	10,433
S34	S27 OR S28 OR S29 OR S30 OR S31 OR S32 OR S33	Expanders - Apply equivalent subjects Search modes - Boolean/Phrase	Interface - EBSCOhost Research Databases Search Screen - Advanced Search Database - Academic Search Elite	38,693
S33	"Early patient discharge"	Expanders - Apply equivalent subjects Search modes - Boolean/Phrase	Interface - EBSCOhost Research Databases Search Screen - Advanced Search Database - Academic Search Elite	14

Formatted: English (United Kingdom)

S32	"Early postpartum discharge"	Expanders - Apply equivalent subjects<br>Search modes - Boolean/Phrase	Interface - EBSCOhost Research Databases<br>Search Screen - Advanced Search<br>Database - Academic Search Elite	31
S31	"very early discharge"	Expanders - Apply equivalent subjects<br>Search modes - Boolean/Phrase	Interface - EBSCOhost Research Databases<br>Search Screen - Advanced Search<br>Database - Academic Search Elite	10
S30	"Reduce length of stay"	Expanders - Apply equivalent subjects	Interface - EBSCOhost Research Databases<br>Search Screen - Advanced	156

		Search modes - Boolean/Phrase	Search Database - Academic Search Elite	
S29	"length of stay"	Expanders - Apply equivalent subjects Search modes - Boolean/Phrase	Interface - EBSCOhost Research Databases Search Screen - Advanced Search Database - Academic Search Elite	37,570
S28	"early discharge"	Expanders - Apply equivalent subjects Search modes - Boolean/Phrase	Interface - EBSCOhost Research Databases Search Screen - Advanced Search Database - Academic Search Elite	1,376
S27	"early postnatal discharge"	Expanders - Apply equivalent subjects Search modes - Boolean/Phrase	Interface - EBSCOhost Research Databases Search Screen - Advanced Search Database - Academic Search Elite	18
S26	S16 OR S17 OR S18 OR S19 OR S20 OR S21 OR S22 OR S23 OR S24 OR S25	Expanders - Apply equivalent subjects Search modes - Boolean/Phrase	Interface - EBSCOhost Research Databases Search Screen - Advanced Search Database - Academic Search Elite	1,036,777

Formatted: English (United Kingdom)

S25	telenursing	Expanders - Apply equivalent subjects<br>Search modes - Boolean/Phrase	Interface - EBSCOhost Research Databases<br>Search Screen - Advanced Search<br>Database - Academic Search Elite	337
S24	telecommunication	Expanders - Apply equivalent subjects<br>Search modes - Boolean/Phrase	Interface - EBSCOhost Research Databases<br>Search Screen - Advanced Search<br>Database - Academic Search Elite	370,174
S23	ehealth	Expanders - Apply equivalent subjects	Interface - EBSCOhost Research Databases<br>Search Screen - Advanced	15,739

		Search modes - Boolean/Phrase	Search Database - Academic Search Elite	
S22	telehealth	Expanders - Apply equivalent subjects Search modes - Boolean/Phrase	Interface - EBSCOhost Research Databases Search Screen - Advanced Search Database - Academic Search Elite	15,607
S21	mhealth	Expanders - Apply equivalent subjects Search modes - Boolean/Phrase	Interface - EBSCOhost Research Databases Search Screen - Advanced Search Database - Academic Search Elite	15,801
S20	"health, mobile"	Expanders - Apply equivalent subjects Search modes - Boolean/Phrase	Interface - EBSCOhost Research Databases Search Screen - Advanced Search Database - Academic Search Elite	84
S19	"mobile health"	Expanders - Apply equivalent subjects Search modes - Boolean/Phrase	Interface - EBSCOhost Research Databases Search Screen - Advanced Search Database - Academic Search Elite	15,910

Formatted: English (United Kingdom)

S18	Telemedicine	Expanders - Apply equivalent subjects<br>Search modes - Boolean/Phrase	Interface - EBSCOhost Research Databases<br>Search Screen - Advanced Search<br>Database - Academic Search Elite	20,104
S17	"health service*"	Expanders - Apply equivalent subjects<br>Search modes - Boolean/Phrase	Interface - EBSCOhost Research Databases<br>Search Screen - Advanced Search<br>Database - Academic Search Elite	582,890
S16	"health system*"	Expanders - Apply equivalent subjects	Interface - EBSCOhost Research Databases<br>Search Screen - Advanced	110,251

		Search modes - Boolean/Phrase	Search Database - Academic Search Elite	
S15	S1 OR S2 OR S3 OR S4 OR S5 OR S6 OR S7 OR S8 OR S9 OR S10 OR S11 OR S12 OR S13 OR S14	Expanders - Apply equivalent subjects Search modes - Boolean/Phrase	Interface - EBSCOhost Research Databases Search Screen - Advanced Search Database - Academic Search Elite	847,395
S14	"home visit*"	Expanders - Apply equivalent subjects Search modes - Boolean/Phrase	Interface - EBSCOhost Research Databases Search Screen - Advanced Search Database - Academic Search Elite	15,677
S13	aftercare	Expanders - Apply equivalent subjects Search modes - Boolean/Phrase	Interface - EBSCOhost Research Databases Search Screen - Advanced Search Database - Academic Search Elite	6,914
S12	"ambulatory care"	Expanders - Apply equivalent subjects Search modes - Boolean/Phrase	Interface - EBSCOhost Research Databases Search Screen - Advanced Search Database - Academic Search Elite	20,405

Formatted: English (United Kingdom)

S11	"managed care"	Expanders - Apply equivalent subjects<br>Search modes - Boolean/Phrase	Interface - EBSCOhost Research Databases<br>Search Screen - Advanced Search<br>Database - Academic Search Elite	18,632
S10	"postnatal care"	Expanders - Apply equivalent subjects<br>Search modes - Boolean/Phrase	Interface - EBSCOhost Research Databases<br>Search Screen - Advanced Search<br>Database - Academic Search Elite	5,141
S9	"home based postnatal care"	Expanders - Apply equivalent subjects	Interface - EBSCOhost Research Databases<br>Search Screen - Advanced	8

		Search modes - Boolean/Phrase	Search Database - Academic Search Elite	
S8	"new parent*"	Expanders - Apply equivalent subjects Search modes - Boolean/Phrase	Interface - EBSCOhost Research Databases Search Screen - Advanced Search Database - Academic Search Elite	1,186
S7	Parent*	Expanders - Apply equivalent subjects Search modes - Boolean/Phrase	Interface - EBSCOhost Research Databases Search Screen - Advanced Search Database - Academic Search Elite	482,962
S6	postpartum	Expanders - Apply equivalent subjects Search modes - Boolean/Phrase	Interface - EBSCOhost Research Databases Search Screen - Advanced Search Database - Academic Search Elite	39,044
S5	multiparas	Expanders - Apply equivalent subjects Search modes - Boolean/Phrase	Interface - EBSCOhost Research Databases Search Screen - Advanced Search Database - Academic Search Elite	1,355

Formatted: English (United Kingdom)

S4	primiparas	Expanders - Apply equivalent subjects<br>Search modes - Boolean/Phrase	Interface - EBSCOhost Research Databases<br>Search Screen - Advanced Search<br>Database - Academic Search Elite	1,283
S3	childbirth	Expanders - Apply equivalent subjects<br>Search modes - Boolean/Phrase	Interface - EBSCOhost Research Databases<br>Search Screen - Advanced Search<br>Database - Academic Search Elite	47,721
S2	birth	Expanders - Apply equivalent subjects	Interface - EBSCOhost Research Databases<br>Search Screen - Advanced	294,546

		Search modes - Boolean/Phrase	Search<br>Database - Academic Search Elite	
S1	"new mother*"	Expanders - Apply equivalent subjects<br>Search modes - Boolean/Phrase	Interface - EBSCOhost Research Databases<br>Search Screen - Advanced Search<br>Database - Academic Search	1,956

Formatted: English (United Kingdom)

Formatted: English (United Kingdom)

Formatted: English (United Kingdom)
