# Supplementary material for: Digital solutions to follow up on discharged new parents—A systematic literature review
Source: PLOS Digit Health. 2023 Aug 23;2(8):e0000317. doi: 10.1371/journal.pdig.0000317 (PMC10446181; doi:10.1371/journal.pdig.0000317)
Supplement: Attachment — Submitted filename: PLOS review.docx [file pdig.0000317.s002.docx]

**Abstract-**

New parents or new mothers? The authors refer to these interchangeably.

**Introduction-**

Sentence is unclear. Suggestion:

*Parity, birth-related factors, and psychosocial ~~predictors~~ factors indicate when it is appropriate for a new mother to be discharged ~~and involved in the organisation of~~ to postnatal care*

Define ‘affinity’.

Only fathers? Or partners of any gender?

Repetition: *There is an international trend in welfare countries with social welfare policies*

Informal tone: ‘…*but is about two days or fewer’* (Suggest- approximately)

*‘…the provision of continuous care’ –* define continuous and the context

*‘perceived sense of security’ –* this seems too subjective – suggest: *perceptions of support*

**Inclusion criteria-**

Refers to ALL types of telemedicine but no reference is made to the exclusion of terms including e-health, m-health or social media

Abstract/introduction refer to follow up including breastfeeding and infant feeding, and to midwives: please justify why these are then excluded from the search terms.

Please justify and discuss implications of your included studies having only four lead authors. This is a very limited insight which means claims are very bold e.g. the wider literature does not support that digital support is adequate without physical postnatal care.

**Results-**

Please define and describe each theme clearly in the opening sentence for each.

Also identify which study contributed to each theme within each discussion – it is not clear where there are common findings and how the review synthesises these. The content of your Overview of themes and codes is much clearer about the differentiations than your writing. (NB This is listed as a figure when it is a table).

For example which study do you refer to here, and is it an isolated finding or a common one?: *In one of the studies, the visual contact established via videoconference facilitated a warm relationship between parents and healthcare professionals.*

Be specific about the platforms identified here: *‘Various digital platforms provided a combination of asynchronous and synchronous methods to facilitate communication with parents out of hospital.’*

All themes need tighter description/names and linking to the supporting studies within the explanations for each. Content overlaps between each theme. E.g.

*Flexibility and convenience of digital support* is a clearer name for theme 1

*Digital literacy* – please remove the references to security and safety here – there is too much crossover with safety themes. Fully explain the implications of digital literacy on accessing this support if this theme has been identified.

*Safe parenting* – implies parenting IS safe rather than parents feeling safe. Suggest this theme is renamed to reflect this, and the inclusion of the significance of being at home as a finding.

*Adequate substitute for physical meetings –* this finding is not explored adequately or linked to the studies and is not supported by the wider literature on postnatal care/ ehealth. Please justify with the evidence form the included studies.

**Discussion-**

*Our findings are supported by Wilson et al.’s study –* formalise sentence structure and place reference within the sentence not at the end throughout. Also expand – how does this evidence support your findings and why is it not included in your review?

Please clarify the relevance of this: *In medical logistics, drones can deliver*

*necessary treatments to remote areas, collect samples, and even provide emergency assistance*

Do not start a sentence with ‘And’ : *And in maternity care, telemedicine platforms have been shown to be clinically effective* *and to improve patient satisfaction [35].*

Specifically, clinically effective how?

This sentence is unclear:

*The present study indicated that new parents were empowered when they had more knowledge of and facility with digital platforms than the health professionals*

The present study? Do you mean this review?

What does more knowledge and facility mean? It implies parents felt empowered if they were better at using digital platforms than professionals?

Please link your claims by referencing the studies you reviewed.

*This observation increases patient autonomy, which in turn affects the care*

*relationship [40].* – How? In what ways? Please be specific and synthesise these findings with the conclusions you draw from your review.

*Parents living in remote areas or lacking the support of relatives or friends are often reliant on telemedicine as a substitute for physical meetings. -*Please reference all such points and link to your findings.

*The expertise obtained from existing telemedicine facilities operating in remote and rural areas has helped to shape digital follow-up after postnatal discharge [46]. –* This is a paragraph which needs expanding and linking to the specific findings of your review – how does this evidence impact that you have reviewed?

Your discussion appears to focus more on other studies than those you have reviewed, and no links are made to these finidngs.

*This review has shown that most new parents have digital literacy, likely driven by the internet and the rapid increase in information and communication technology –* this is an overly bold statement based on very few studies with even fewer authors. Consider ‘many new parents may….’

*according to the eHealth competence model) –* please define and expand on this and its use in exploring your findings.

*how the concept of care changes when, for instance, robots are introduced as part of*

*the care-giving process –* define and expand – what concept of care? How does it change it? How does this impact parents/ the potential of online support?

*it should be noted that the care received after discharge may be experienced differently than a home visit from a healthcare professional.* – on what ways may it be experienced differently? What impact may it have (other reviews have noted the importance of concurrent support across settings)

*Following a recent study describing autonomy when interacting or using digital tools as relational and situated [52], autonomy needs to be seen in context and in relation to the users’ situated abilities –* define and explain these terms and the relevance of them to parents.

*However, one aspect that has not been addressed is whether new parents experience the use of digital solutions differently.* Differently to whom?

*Upon discharge from hospital, new parents – even those with high digital literacy – may find it challenging or overwhelming to use digital tools whilst also caring for a newborn.* Reference your sources.

*Indeed, telemedicine has proven to be important for women with low socioeconomic status, particularly in relation to addressing postnatal mental health [55].* How does this relate to the previous point?

*~~In the future, more advanced platforms or technical solutions will be available, such as social~~*

*~~and assistive robots. Robots could be used to assist with practical tasks within the home (e.g.,cleaning, and collecting and transporting items within the home) and for eHealth purposes (e.g., getting in touch with a healthcare professional or giving advice to new parents). Such robots have been tested with older adults – see for instance, the use of the Giraff robot [56-58] or other social and assistive robots (SARs) [59]. However, using SARs to support new parents following discharge remains understudied and represents an area for further research. Newly discharged parents often need help with practical tasks around the home so they can focus on their newborn; some of these can potentially already be allocated to robots [50] as they do not involve direct, intimate parent–robot interaction. On the other hand, tasks that involve more 16 interaction between the robot and the newly discharged parents, like moving and bringing items, giving advice, and helping parents get in touch with healthcare professionals, may be interesting to investigate further. However, privacy, safety, and security issues may arise depending on how advanced the robot is and what equipment is used (e.g., cameras and sensors) [60]. Rigorous pilot testing and proper clinical trials are therefore needed before these digital applications are offered to new parents discharged early from hospital.~~*

The above seems irrelevant to your findings and the scope of your review. You need to properly define the parameters of ‘postnatal care/support’. You have also not explained the terms discussed e.g Giraff robot and what they do. Please remove or clearly explain the links to your findings.

**Implications for practice**

*After early home discharge, new parents should have reliable, 24-hour access to information*

*related to newborn care, breastfeeding, and parenthood. -* You have not clearly identified how and which of your themes have demonstrated this.

*This is especially important when supporting breastfeeding while also ensuring security and cultural appropriateness. -* Other than in your abstract you have not referred anywhere to breastfeeding support, nor included this in your search terms – this is therefore not a claim supported b your review.

*Today’s new parents tend to be digitally literate and are thus very likely to make use of telemedicine.* – again there are no figures or references to the literature to justify this claim. I suggest Statista.

Conclusion

*in contrast, problems can arise if parents lack access to digital resources after early discharge. –* You have not demonstrated this to be the case with your findings or links to wider evidence.

*Finally, digital literacy is important for both healthcare professionals and patients receiving care*

*through telemedicine. –* You have not provided sources or reference to digital literacy amongst professionals elsewhere

*This area needs to be developed in the healthcare system, especially among midwives, who mainly provide this support in the context of postnatal follow-up care.* Again you need to explore the literature on midwives and digital support to claim this. See Morse & Brown, 2021 and 2022.

**Additionally:** you have provided no limitations to your review of which there are several, including the limited number of included papers, the search terms which do not include breastfeeding or social media or midwives. Please add a limitations section.
