# Supplementary material for: Digital solutions to follow up on discharged new parents—A systematic literature review
Source: PLOS Digit Health. 2023 Aug 23;2(8):e0000317. doi: 10.1371/journal.pdig.0000317 (PMC10446181; doi:10.1371/journal.pdig.0000317)
Supplement: Attachment — Submitted filename: 03-05-2023 Response to Reviewers .docx [file pdig.0000317.s003.docx]

# **Response to reviewers**

# **[PDIG-D-23-00068] - [EMID:ea1d41519e19641e] Digital solutions to follow up on discharged new parents a systematic literature review**

We are very grateful for your willingness to consider our manuscript for publication in PLOS Digital Health. We would like to thank the editor and reviewers for their constructive and thoughtful feedback and have revised the manuscript according to the reviewers’ concerns below. Changes in the manuscript are highlighted in colour in a separate attached file.

| Editor Comments | Response to the Editor |
| --- | --- |
| Please choose appropriate keywords based on the MeSH terms. | After checking in MeSH NCBI, the keywords are updated as followed:  Ambient-Assisted Living, eHealth, telehealth, parents, postpartum period, patient discharge (lines 44-45) |
| Please merge the aim of the study with the rest of the introduction section. Moreover, please justify why conducting a SLR was necessary | Aim is merged.  We added followed text (lines 91-95)  With this review, we want to increase our knowledge of new parents' subjective perceptions, evaluations and interpretations regarding digital follow-up from the care provider's side after discharge from the maternity ward. The new understanding will give us insight into what is perceived to be working well and what challenges remain. |
| Please make sure that the structure of your manuscript (headings and subheadings) has been organized based on the PRISMA checklist. Please upload the completed check list along with your revised manuscript | Thanks for the comment; this is now under control and subheadings are named as in the PRISMA checklist |
| In the methods section, please justify why only qualitative studies were included | Added lines 111-112 |
| Please explain why databases such as Scopus and Web of Knowledge were not used while these are the main databases for conducting a SLR | We included databases that mainly have research in nursing and health areas, but at your suggestion, we carried out searches in Scopus and have not found any new studies that answer the purpose of the study.  Web of Knowledge we do not have access to via our library |
| Is there any reason for choosing the timeline between 2015 and 2022 | Anledningen till att vi valde denna tidsperiod är baserad på att hitta alla de senaste studierna som svarar på syftet. |
| Please add a list of search strategies for all databases as an Appendix | We attached text from Word documents, and searches generated as PDF are uploaded separately |
| In Figure 1, excluding papers after reading the full text, the authors excluded 7 papers indicating that “7 had a low CASP assessment”. This needs to be expanded in the methods section and please support it with a reference. Moreover, in this box, reasons other than the CASP score need to be reported | Added lines 152- 153 CASP assessment ranges from 0 -10. Ten points mean that the study is of high quality. See the attachment in the manus after section references Table 1. CASP assessment of included studies |
| Table 3 is a bit difficult to follow. Could you please remove or summarize it |  |
| Please re-check the captions of the Figures and Tables. In my opinion, e.g. Figure 3 is a Table not a Figure | Removed, but attached as an attachment to the request (above) to show all searches/strategies in all databases |
| Reviewer #1 | Response to Reviewer #1 |
| 1) Objectives: “This systematic review aims to integrate available research results that describe parents’ perceived sense of security when health and care providers used telemedicine as a platform for follow-up.” The main objective of the study is not parallel to the results and discussion. Sense of security is too vague and does not reflect the themes stated in the introduction. | Thanks for the important comment, we adjusted the purpose more clearly now  This systematic review aims to integrate available research results that describe parents’ experiences when health and care providers used telemedicine as a platform for follow-up |
| 2) Methodology: How was “sense of security” assessed/measured? Was it uniform among the studies that were selected? | This cannot be measured in a qualitative study, only described as an experience. The experiences are what is common to included studies |
| 3) Whole Results section - Please put specific in-text citations or supporting references for the sentences in the Results section. The references were only placed as a range “[24-31]” at the start of every section. It would be better to place the specific reference/s after every statement or so. This would help us readers check the references faster in relation to the claims in the results. | All results from selected studies constituted the unit of analysis. The analysis went across the entire analysis unit to find themes that answered the purpose of the study. Therefore, all included articles were referred to under the respective theme. |
| 4) Results - Flexible Digital Support: “Various digital platforms provided a combination of asynchronous and synchronous methods to facilitate communication with parents out of hospital. Asynchronous communication offered an accessible way to obtain help after early discharge from maternity units.” Maybe you can discuss and expound more on the asynchronous and synchronous methods used in the studies. What asynchronous methods were used? What synchronous methods were used? Are all of them effective and useful for follow ups? | Explanation added at lines 175-176 |
| 5) Discussion: The discussion was well-written but it would be better to align it to the objective and results. Emphasize the answer to your objective. Emphasize on discussing your results. | thanks for the comment, we've aligned the most important results with aim of the study this on lines 282-283 |
| 1) Typographical error: Please add a period at the end of the sentence in the objective “This systematic review aims to integrate available research results that describe parents’ perceived sense of security when health and care providers used telemedicine as a platform for follow-up” | Added |
| Reviewer #2 | Response to Reviewer #2 |
| Abstract, New parents or new mothers? The authors refer to these interchangeably. | thanks for this comment, we have now corrected this throughout the text |
| Introduction- Sentence is unclear. Suggestion: Parity, birth-related factors, and psychosocial predictors factors indicate when it is appropriate for a new mother to be discharged and involved in the organisation of to postnatal care | Thanks for the generous suggestion, we accept it and put it in text lines 48-50 |
| Define ‘affinity’. | thanks for the comment, it was completely the wrong word, it should say family bond see line 53 |
| Only fathers? Or partners of any gender? | thanks for comment, it should say partners |
| Repetition: There is an international trend in welfare countries with social welfare policies | we could not find duplicates but we shortened the text a little line 72 |
| Informal tone: ‘…but is about two days or fewer’ (Suggest- approximately) | thanks, accepted proposal line 75 |
| ‘…the provision of continuous care’ – define continuous and the context | should it be written Continuity of care is concerned with the **quality of care over time line 94** |
| ‘perceived sense of security’ – this seems too subjective – suggest: perceptions of support | thanks for the comment. These concepts are gone and replaced with experience |
| Inclusion criteria- Refers to ALL types of telemedicine but no reference is made to the exclusion of terms including e-health, m-health or social media | thanks for the comment, this is now revised line 129-130 |
| Abstract/introduction refer to follow up including breastfeeding and infant feeding, and to midwives: please justify why these are then excluded from the search terms. | the introduction describes how and who follows up new parents, this has nothing to do with inclusion criteria or search terms |
| Please justify and discuss implications of your included studies having only four lead authors. This is a very limited insight which means claims are very bold e.g. the wider literature does not support that digital support is adequate without physical postnatal care. | Thanks för the comment, we have clarified that on lines 382-386 |
| Results- Please define and describe each theme clearly in the opening sentence for each.  Also identify which study contributed to each theme within each discussion – it is not clear where there are common findings and how the review synthesises these. The content of your Overview of themes and codes is much clearer about the differentiations than your writing. (NB This is listed as a figure when it is a table). | All results from selected studies constituted the unit of analysis. The analysis went across the entire analysis unit to find themes that answered the purpose of the study. Therefore, all included articles were referred to under the respective theme. The overview of themes and codes illustrates how the analysis has been carried out and can be seen as a complement to the method description and stops there. Under results, each theme is described separately without again reproducing how they came about. |
| Be specific about the platforms identified here: ‘Various digital platforms provided a combination of asynchronous and synchronous methods to facilitate communication with parents out of hospital.’ | Explanation added at lines 175-176 |
| All themes need tighter description/names and linking to the supporting studies within the explanations for each. Content overlaps between each theme. E.g. | All results from selected studies constituted the unit of analysis. The analysis went across the entire analysis unit to find themes that answered the purpose of the study. Therefore, all included articles were referred to under the respective theme. The overview of themes and codes illustrates how the analysis has been carried out and can be seen as a complement to the method description and stops there. Under results, each theme is described separately without again reproducing how they came about. |
| Flexibility and convenience of digital support is a clearer name for theme 1  Digital literacy – please remove the references to security and safety here – there is too much crossover with safety themes. Fully explain the implications of digital literacy on accessing this support if this theme has been identified. | Thanks for the suggestion; we accept it. Line 180  As for the references, let's have them here since the themes are the result of included articles. The themes are the result of qualitative analysis of the results of included articles. This means that we describe the interpreted themes, and it does not work if we follow the method of giving a full explanation that is requested. . Thematization means a higher degree of organization and level of abstraction of the material. |
| Safe parenting – implies parenting IS safe rather than parents feeling safe. Suggest this theme is renamed to reflect this, and the inclusion of the significance of being at home as a finding. | Thanks for the suggestion; we accept it. Line 249 |
| adequate substitute for physical meetings – this finding is not explored adequately or linked to the studies and is not supported by the wider literature on postnatal care/ ehealth. Please justify with the evidence form the included studies. | Thanks for the comment, but we disagree with the reviewer. As explained above, themes result from qualitative analysis and cannot be explained as requested. Thematization means a higher degree of organization and level of abstraction of the material. |
| Discussion-Our findings are supported by Wilson et al.’s study – formalise sentence structure and place reference within the sentence not at the end throughout. Also expand – how does this evidence support your findings and why is it not included in your review? | Thanks for pointing out. We have revised the text on lines 291-295. Wilson et al. did not meet the inclusion criteria and could not be included in this study's results. But we have chosen to reflect the results of our study with it. |
| Please clarify the relevance of this: In medical logistics, drones can deliver necessary treatments to remote areas, collect samples, and even provide emergency assistance | We have clarified the meaning of lines 303- 304 |
| Do not start a sentence with ‘And’ : And in maternity care, telemedicine platforms have been shown to be clinically effective and to improve patient satisfaction [35].  Specifically, clinically effective how? | Corrected line 306 |
| This sentence is unclear: The present study indicated that new parents were empowered when they had more knowledge of and facility with digital platforms than the health professionals | Corrected line 309 |
| The present study? Do you mean this review?  What does more knowledge and facility mean? It implies parents felt empowered if they were better at using digital platforms than professionals?  Please link your claims by referencing the studies you reviewed. | Correct  This introduction summary of the themes Digital literacy, referenced |
| This observation increases patient autonomy, which in turn affects the care relationship [40]. – How? In what ways? Please be specific and synthesise these findings with the conclusions you draw from your review. | Thanks for this comment, criticism is on, place and we have removed the text |
| Parents living in remote areas or lacking the support of relatives or friends are often reliant on telemedicine as a substitute for physical meetings. -Please reference all such points and link to your findings. | corrected |
| The expertise obtained from existing telemedicine facilities operating in remote and rural areas has helped to shape digital follow-up after postnatal discharge [46]. – This is a paragraph which needs expanding and linking to the specific findings of your review – how does this evidence impact that you have reviewed? | clarified on lines 326-328 |
| Your discussion appears to focus more on other studies than those you have reviewed, and no links are made to these findings. | Discussion is structured in this way: summary of the most important findings in our review and then mirrored with studies that either confirm or say otherwise. |
| This review has shown that most new parents have digital literacy, likely driven by the internet and the rapid increase in information and communication technology – this is an overly bold statement based on very few studies with even fewer authors. Consider ‘many new parents may….’  according to the eHealth competence model) – please define and expand on this and its use in exploring your findings. | Thanks for the comment; Reference should be made to Reddy et al. see line 329 |
| how the concept of care changes when, for instance, robots are introduced as part of the care-giving process – define and expand – what concept of care? How does it change it? How does this impact parents/ the potential of online support? | Thanks for this comment, criticism is on, place and we have revised text line 343-344 |
| it should be noted that the care received after discharge may be experienced differently than a home visit from a healthcare professional. – on what ways may it be experienced differently? What impact may it have (other reviews have noted the importance of concurrent support across settings) | Thanks for this comment, criticism is on, place and we have removed the text |
| Following a recent study describing autonomy when interacting or using digital tools as relational and situated [52], autonomy needs to be seen in context and in relation to the users’ situated abilities – define and explain these terms and the relevance of them to parents. | Thanks for this comment, criticism is on, place and we have removed the text |
| However, one aspect that has not been addressed is whether new parents experience the use of digital solutions differently. Differently to whom? | Thanks for this comment, criticism is on, place and we have removed the text |
| Upon discharge from hospital, new parents – even those with high digital literacy – may find it challenging or overwhelming to use digital tools whilst also caring for a newborn. Reference your sources. | This is the reviewer's conclusion. We base our conclusion on an analysis of included articles |
| Indeed, telemedicine has proven to be important for women with low socioeconomic status, particularly in relation to addressing postnatal mental health [55]. How does this relate to the previous point?  In the future, more advanced platforms or technical solutions will be available, such as social and assistive robots. Robots could be used to assist with practical tasks within the home (e.g.,cleaning, and collecting and transporting items within the home) and for eHealth purposes (e.g., getting in touch with a healthcare professional or giving advice to new parents). Such robots have been tested with older adults – see for instance, the use of the Giraff robot [56-58] or other social and assistive robots (SARs) [59]. However, using SARs to support new parents following discharge remains understudied and represents an area for further research. Newly discharged parents often need help with practical tasks around the home so they can focus on their newborn; some of these can potentially already be allocated to robots [50] as they do not involve direct, intimate parent–robot interaction. On the other hand, tasks that involve more 16 interaction between the robot and the newly discharged parents, like moving and bringing items, giving advice, and helping parents get in touch with healthcare professionals, may be interesting to investigate further. However, privacy, safety, and security issues may arise depending on how advanced the robot is and what equipment is used (e.g., cameras and sensors) [60]. Rigorous pilot testing and proper clinical trials are therefore needed before these digital applications are offered to new parents discharged early from hospital.  The above seems irrelevant to your findings and the scope of your review. You need to properly define the parameters of ‘postnatal care/support’. You have also not explained the terms discussed e.g Giraff robot and what they do. Please remove or clearly explain the links to your findings. | Thanks for this comment, criticism is on, place and we have removed the text |
| Implications for practice  After early home discharge, new parents should have reliable, 24-hour access to information related to newborn care, breastfeeding, and parenthood. - You have not clearly identified how and which of your themes have demonstrated this. | Thanks for this comment, criticism is on, place and we have revised the headline lines 388-390 |
| This is especially important when supporting breastfeeding while also ensuring security and cultural appropriateness. - Other than in your abstract you have not referred anywhere to breastfeeding support, nor included this in your search terms – this is therefore not a claim supported b your review. | Thanks for this comment, criticism is on, place and we have revised the headline lines 388-390 |
| oday’s new parents tend to be digitally literate and are thus very likely to make use of telemedicine. – again there are no figures or references to the literature to justify this claim. I suggest Statista. | Thanks for this comment, criticism is on, place and we have revised the headline lines 388-390 |
| Conclusion  in contrast, problems can arise if parents lack access to digital resources after early discharge. – You have not demonstrated this to be the case with your findings or links to wider evidence.  Finally, digital literacy is important for both healthcare professionals and patients receiving care through telemedicine. – You have not provided sources or reference to digital literacy amongst professionals elsewhere | Thanks for this comment, criticism is on, place and we have revised the headline 393-399 |
| This area needs to be developed in the healthcare system, especially among midwives, who mainly provide this support in the context of postnatal follow-up care. Again you need to explore the literature on midwives and digital support to claim this. See Morse & Brown, 2021 and 2022.  Additionally: you have provided no limitations to your review of which there are several, including the limited number of included papers, the search terms which do not include breastfeeding or social media or midwives. Please add a limitations section. | Thanks for this comment, criticism is on, place and we have revised the headline lines 393-399 |
