# Supplementary material for: Digital solutions to follow up on discharged new parents—A systematic literature review
Source: PLOS Digit Health. 2023 Aug 23;2(8):e0000317. doi: 10.1371/journal.pdig.0000317 (PMC10446181; doi:10.1371/journal.pdig.0000317)
Supplement: Attachment — Submitted filename: 31-05-2023 second Response to Reviewers .docx [file pdig.0000317.s005.docx]

# **Second Response to reviewers**

# **PLOS Digital Health Decision: Revision required [PDIG-D-23-00068R1] - [EMID:de4ec2188cd5bb38]**

**PDIG-D-23-00068R1**

**Digital solutions to follow up on discharged new parents a systematic literature review PLOS Digital Health**

We are very grateful for your willingness to consider our manuscript for publication in PLOS Digital Health. We would like to thank the editor and reviewers for their constructive and thoughtful feedback and have revised the manuscript according to the reviewers’ concerns below. Changes in the manuscript are highlighted in colour in a separate attached file.

| Editor Comments | Response to the Editor |
| --- | --- |
| In all tables, please report the studies chronologically. | we usually present included studies in alphabetical order but have now presented them chronologically based on year of publication |
| Please make sure that all of the submitted materials are in English. | we have gone through appendices that illustrate different searches in databases and translated some words from Norwegian to English |
| Regarding the search strategies, I think it is enough if you please provide a table and put the name of each database along with the final search strategy for that database in the table. | Thanks for the suggestions; we have saved all search strategies in the database. We have checked through and translated Norwegian words into English. At the request of for review, we upload all saved searches. They can be used as extra attachments to illustrate the search. |
| Reviewer | Response to Reviewer |
| Reviewer #2: There are some comments which have not been addressed - please see attached document. The main revision required as also stated by the other reviewer is to cite the specific study each claim relates to under each theme, so the reader can identify how the reviewed work relates to the findings. I suggest looking at similar reviews using TA if how this can be done is not clear for example  Lines 178, 212-214, 247, 272, As previously - you should allocate individual references to your claims so the reader can identify which if these findings are from which study. Unless the implication is every study had the same identical findings which suggests your review is too narrow | The reviewer's proposal aims at a simpler overview grouping of the results. Regarding the thematic analysis that we chose and carried out, the text data analysis unit is built from the output from all included articles. The text from the results is analyzed based on the purpose of the study. Based on the entire text, themes have been generated and summarized narratively. Therefore, all included articles are referred to as a group and not a single article. |
| Upon discharge from hospital, new parents – even those with high digital literacy – may find it challenging or overwhelming to use digital tools whilst also caring for a newborn. Reference your sources. | Unfortunately, we cannot find this text in the revised script. Most likely this text has been removed. |
| Lines 12-122 This is unclear. Does it mean - All digital delivery formats including web and app based? | Thanks for a very good suggestion, we accept it in full |
| Line 288 . Also reference Wilson et al correctly. | corrected |
| Line 322 The review by Reddy et al [ ] | corrected |
| Line 375-378 Please expand with detail - why was this? And what are the implications (small sample and limited generalisability) | thanks for comment, text removed |
