# Supplementary material for: Digital solutions to follow up on discharged new parents—A systematic literature review
Source: PLOS Digit Health. 2023 Aug 23;2(8):e0000317. doi: 10.1371/journal.pdig.0000317 (PMC10446181; doi:10.1371/journal.pdig.0000317)
Supplement: Attachment — Submitted filename: 07-02-2023 third Response to Reviewers and Editor.docx [file pdig.0000317.s006.docx]

# **Response to Reviewers and Editor**

PDIG-D-23-00068R2

Digital solutions to follow up on discharged new parentsa systematic literature review PLOS Digital Health

We are very grateful for your willingness to consider our manuscript for publication in PLOS Digital Health. We would like to thank the editor and reviewers for their constructive and thoughtful feedback and have revised the manuscript according to the reviewers’ concerns below. Changes in the manuscript are highlighted in colour in a separate attached file.

| **Comments** | **Response to the Editor** |
| --- | --- |
| The title has not been edited in the submission system. Please edit it. | Revised and added |
| After Table 1, there is still another table with empty cells. Please remove it. | Removed |
| Tables 3 shows “Overview of themes and codes”. In the systematic reviews, we usually do not present codes. | Removed |
| Figure 3, actually it is not a Figure. It is a table which shows themes and subthemes. Again, in the systematic reviews, we usually do not present such a table, but the results section can be divided based on the themes and subthemes | Removed |
| In terms of the search strategies, just one final search strategy for each database is enough. Search strategies for different databases can be presented in one table to be submitted as a supplementary file | Revised and added |
